# Interim safety and immunogenicity results from an NDV-based COVID-19 vaccine phase I trial in Mexico

**DOI:** 10.1038/s41541-023-00662-6

**Published:** 2023-05-10

**Authors:** Samuel Ponce-de-León, Martha Torres, Luis Enrique Soto-Ramírez, Juan José Calva, Patricio Santillán-Doherty, Dora Eugenia Carranza-Salazar, Juan Manuel Carreño, Claudia Carranza, Esmeralda Juárez, Laura E. Carreto-Binaghi, Luis Ramírez-Martínez, Georgina Paz De la Rosa, Rosalía Vigueras-Moreno, Alejandro Ortiz-Stern, Yolanda López-Vidal, Alejandro E. Macías, Jesús Torres-Flores, Oscar Rojas-Martínez, Alejandro Suárez-Martínez, Gustavo Peralta-Sánchez, Hisaaki Kawabata, Irene González-Domínguez, José Luis Martínez-Guevara, Weina Sun, David Sarfati-Mizrahi, Ernesto Soto-Priante, Héctor Elías Chagoya-Cortés, Constantino López-Macías, Felipa Castro-Peralta, Peter Palese, Adolfo García-Sastre, Florian Krammer, Bernardo Lozano-Dubernard

**Affiliations:** 1 Programa Universitario de Investigación en Salud (PUIS), Facultad de Medicina, Universidad Nacional Autónoma de México (UNAM), Edif. de los Programas Universitarios, Planta Alta. Circuito de la Investigación Científica S/N Ciudad Universitaria, Ciudad de México, C.P. 04510 México; 2 Laboratorio de Inmunobiología de la tuberculosis, Instituto Nacional de Enfermedades Respiratorias (INER), Ismael Cossio Villegas, Calzada de Tlalpan 4502, Sección XVI, CP 14080 Tlalpan, México; 3 Department of Infectious Diseases, Instituto Nacional de Ciencias Médicas y Nutrición “Salvador Zubirán”, Vasco de Quiroga 15, Belisario Dominguez, Sección XVI, 14080 Tlalpan, México; 4 Departamento de Infectología y Vigilancia Epidemiológica, Hospital Médica Sur, S.A.B. de C. V., Puente de Piedra 150, Toriello Guerra, 14050 Tlalpan, México; 5 Instituto Nacional de Enfermedades Respiratorias (INER), Ismael Cossio Villegas, Calzada de Tlalpan 4502, Sección XVI, CP 14080 Tlalpan, México; 6 ProcliniQ Investigación Clínica, S. A. de C. V., Renato Leduc 155 (Xontepec 91), Toriello Guerra, 14050 Tlalpan, México; 7 Department of Microbiology, Icahn School of Medicine at Mount Sinai, 1 Gustave L. Levy Pl, New York, NY 10029 USA; 8 Departamento de Investigación en Microbiología, Instituto Nacional de Enfermedades Respiratorias (INER), Ismael Cossio Villegas, Calzada de Tlalpan 4502, Sección XVI, CP 14080 Tlalpan, México; 9 Laboratorio Avi-Mex, S. A. de C. V. (Avimex), Maíz 18, Granjas Esmeralda, CP 09810 Iztapalapa, CDMX Mexico; 10 iLS Clinical Research, S. C. (iLS), Matias Romero 102 - 205 Del Valle, Benito Juárez, CP 03100 CDMX México; 11 Programa de Inmunología Molecular Microbiana, Departamento de Microbiología y Parasitología, Facultad de Medicina, Universidad Nacional Autónoma de México (UNAM), Av. Universidad 3000, Circuito Interior S/N. Ciudad Universitaria, Coyoacán, CP.04510 México; 12 Departamento de Medicina, Universidad de Guanajuato, 20 de Enero 929, C.P 37000 León Guanajuato, México; 13 Dirección Adjunta de Desarrollo Tecnológico, Vinculación e Innovación, Consejo Nacional de Ciencia y Tecnología (CONACYT), Insurgentes Sur 1582, Crédito Constructor, CP 03940 Benito Juárez, CDMX México; 14 Consultora Mextrategy, S.A.S. de C. V. (Mextrategy), Insurgentes Sur 1079 P7-127, Nochebuena, CP 03720 CDMX Mexico; 15 Unidad de Investigación Médica en Inmunoquímica. Hospital de Especialidades del Centro Médico Nacional Siglo XXI. Instituto Mexicano del Seguro Social (IMSS), Av. Cuauhtémoc 330, Doctores, C.P. 06720 Benito Juárez, CDMX México; 16 Department of Medicine, Icahn School of Medicine at Mount Sinai, 1 Gustave L. Levy Pl, New York, NY 10029 USA; 17 Global Health and Emerging Pathogens Institute, Icahn School of Medicine at Mount Sinai, 1 Gustave L. Levy Pl, New York, NY 10029 USA; 18 The Tisch Cancer Institute, Icahn School of Medicine at Mount Sinai, 1 Gustave L. Levy Pl, New York, NY 10029 USA; 19 Department of Pathology, Molecular and Cell-based Medicine, Icahn School of Medicine at Mount Sinai, 1 Gustave L. Levy Pl, New York, NY 10029 USA

**Keywords:** Live attenuated vaccines, Live attenuated vaccines

## Abstract

There is still a need for safe, efficient, and low-cost coronavirus disease 2019 (COVID-19) vaccines that can stop transmission of severe acute respiratory syndrome coronavirus 2 (SARS-CoV-2). Here we evaluated a vaccine candidate based on a live recombinant Newcastle disease virus (NDV) that expresses a stable version of the spike protein in infected cells as well as on the surface of the viral particle (AVX/COVID-12-HEXAPRO, also known as NDV-HXP-S). This vaccine candidate can be grown in embryonated eggs at a low cost, similar to influenza virus vaccines, and it can also be administered intranasally, potentially to induce mucosal immunity. We evaluated this vaccine candidate in prime-boost regimens via intramuscular, intranasal, or intranasal followed by intramuscular routes in an open-label non-randomized non-placebo-controlled phase I clinical trial in Mexico in 91 volunteers. The primary objective of the trial was to assess vaccine safety, and the secondary objective was to determine the immunogenicity of the different vaccine regimens. In the interim analysis reported here, the vaccine was found to be safe, and the higher doses tested were found to be immunogenic when given intramuscularly or intranasally followed by intramuscular administration, providing the basis for further clinical development of the vaccine candidate. The study is registered under ClinicalTrials.gov identifier NCT04871737.

## Introduction

Severe acute respiratory syndrome coronavirus 2 (SARS-CoV-2) emerged in China in late 2019 and has since then caused the coronavirus disease 2019 (COVID-19) pandemic^1,2^. Vaccines against SARS-CoV-2 were rapidly developed and have been shown to be safe and efficacious^3^. However, in many low- and middle-income countries (LMICs), access to vaccines is still limited. In addition, mRNA-based COVID-19 vaccines require frozen storage and transportation—severely restricting their usability in LMICs. Furthermore, the production of many of the available COVID-19 vaccines is costly, affecting the price per dose. In addition, all currently approved COVID-19 vaccines are injected intramuscularly, leading to strong systemic but absent or weak mucosal immunity^4^, which is thought to be critical for achieving sterilizing immunity against SARS-CoV-2. Moreover, for more infectious variants like B.1.617.2 (Delta), the rate of breakthrough infections has increased^5^ and has now peaked with the emergence of B.1.1.529 (Omicron)^6–13^. These breakthrough infections are often asymptomatic or mild if symptomatic, and protection from severe disease remains high^14,15^. However, the fact that they occur is likely a consequence of the absence of persistent mucosal immunity, which can neutralize the virus right at its entry point into the body on mucosal surfaces of the upper respiratory tract. Vaccines that potentially induce mucosal immunity may be better suited to induce sterilizing immunity and block transmission of a virus^16–18^.

To address the issues raised above, we have developed a live Newcastle disease virus (NDV)-based SARS-CoV-2 vaccine. NDV is an avian paramyxovirus that is highly attenuated in mammals and has been tested in humans as an oncolytic virus and in preclinical models as a live vaccine vector^19–27^. We engineered the LaSota vaccine strain of NDV to express the spike protein of SARS-CoV-2^28–30^. The version of the spike protein used is based on an enhanced immunogen design, which includes six proline mutations and a deletion of the polybasic cleavage site keeping the spike in a stable pre-fusion conformation^31^. In addition, the ectodomain of the spike protein was grafted onto the transmembrane domain and cytoplasmic domain of the NDV fusion protein to ensure optimal incorporation into the Newcastle disease virion. The vaccine vector, therefore, carries the spike on its surface and expresses it in cells that it infects.

NDV is an avian virus, and it can be grown in embryonated chicken eggs to very high titers. Embryonated eggs are used for the production of the majority of influenza virus vaccines used, and therefore, production capacity for this NDV-vectored vaccine already exists in high-income and LMICs^32^. This also allows the vaccine to be produced at a very low cost.

We have previously shown that an inactivated, as well as a live version of this NDV-vectored vaccine, is safe, well tolerated, highly immunogenic, and protective in animal models, including in a swine model using different routes of administration. These data contributed to the design of the phase I protocol reported herein^28–30,33–35^. Inactivated versions of the vaccine are currently in clinical development in Vietnam (NCT04830800), Brazil (NCT04993209), and Thailand (NCT04764422). Interim results from Thailand show that the inactivated formulation—which is injected intramuscularly—is safe and highly immunogenic^34^. Here, we tested a live version of the vaccine, AVX/COVID-12-HEXAPRO (Patria, also known as NDV-HXP-S), in an open-label, non-randomized non-placebo-controlled phase I trial in 91 healthy volunteers. The vaccine was administered either via an intramuscular prime-boost regimen or, for optimal induction of mucosal immunity, via an intranasal prime-boost regimen. In addition, intranasal immunization followed by an intramuscular administration was also tested. Below, we report the interim safety and immunogenicity results from this trial in Mexico (NCT04871737).

## Results

### Participant disposition

**Fig. 1:**
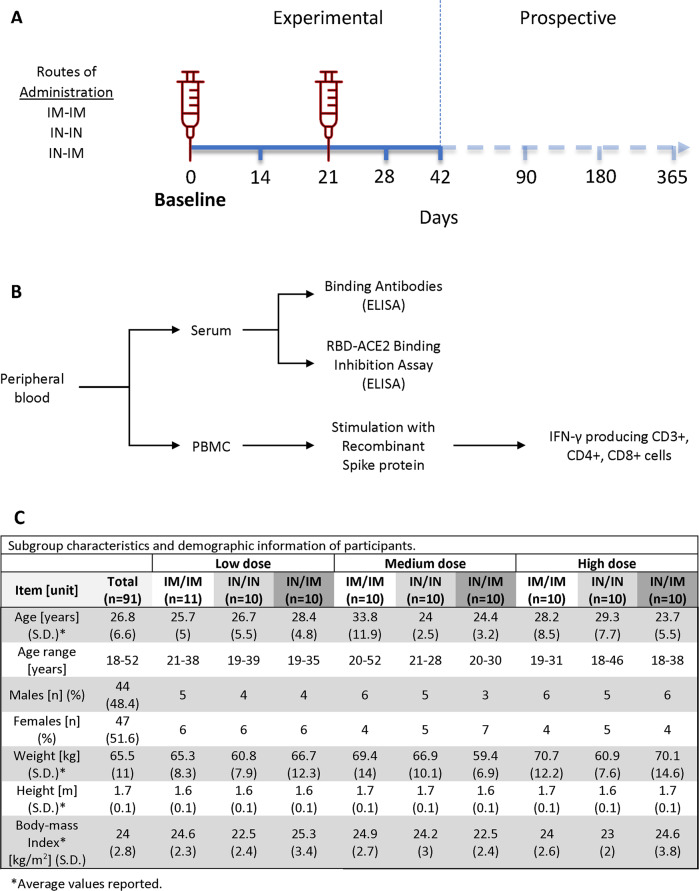
Study design and group distribution. **A** Schematic representation of the study timeline, indicating routes of administration, vaccination time points, and sample collection for immunogenicity analyses. The three different vaccination regiments tested; intramuscular (IM) followed by intramuscular (IM), intranasal (IN) followed by intranasal (IN), and intranasal (IN) followed by intramuscular (IM) administration are shown on the left. Time points of sample collection (0, 14, 21, 28, 42, 90, 180, and 365 days after the first vaccine dose administration) and time points of vaccine administration (indicated by the red syringe) are shown on the right. **B** Diagram depicting specimen types collected to assess immunogenicity. **C** Subgroup characteristics and demographic information of participants of the trial (*n* = 91).

**Fig. 2:**
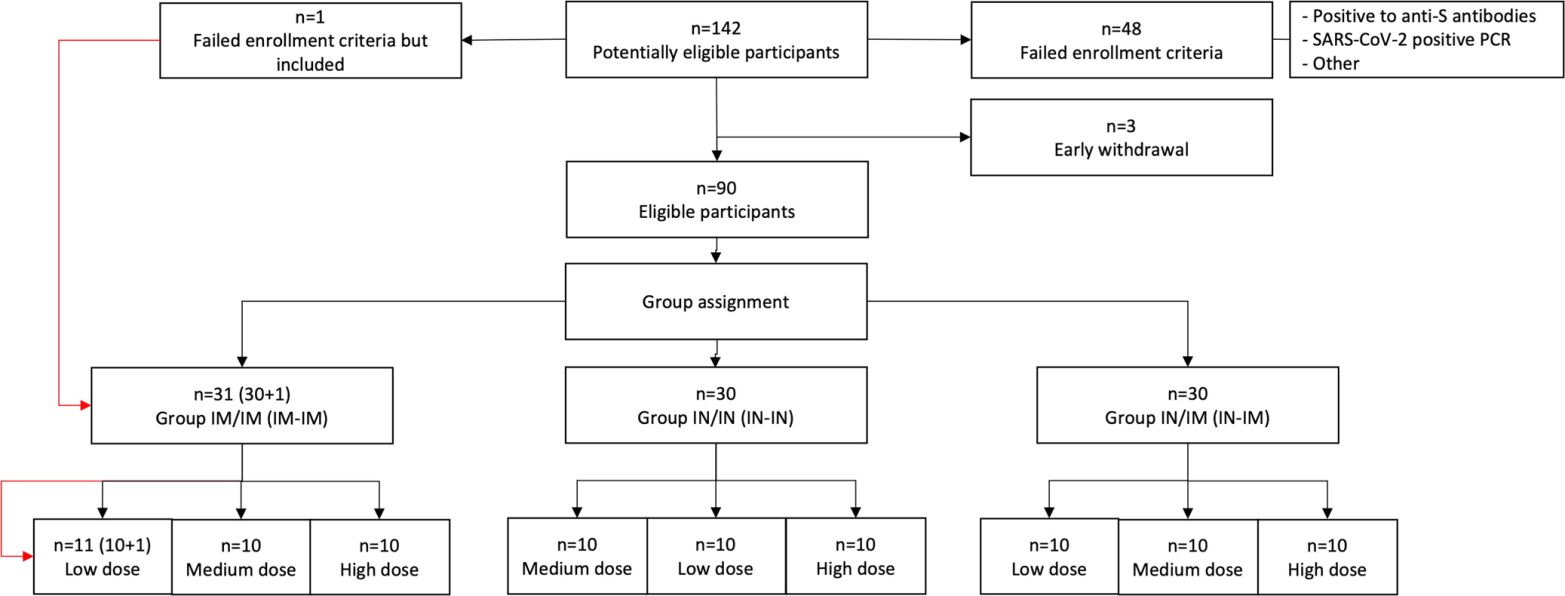
Enrollment and sub-randomization. Diagram representing the number of participants initially screened (*n* = 142), failed enrollment criteria (*n* = 48), early withdrawals (*n* = 3), and eligible participants (*n* = 90) that were included in the trial and assigned to any of the three vaccination regimens (*n* = 30, per group) and dose (low *n* = 10, medium *n* = 10, high *n* = 10). A participant that initially was considered eligible and received an IM-IM regimen but subsequently failed study criteria due to SARS-CoV-2 antibody positivity is indicated on the left. A detailed description of enrollment failures can be found in Supplementary Table 3.

From May 24, 2021, to August 20, 2021, 142 volunteers were assessed. Three subjects voluntarily withdrew from the study before group assignment, while 48 were excluded as they did not meet eligibility criteria (Supplementary Table 3). Ninety eligible volunteers were enrolled into nine different groups and either dosed twice IM (IM-IM groups), dosed sequentially IN followed by IM (IN-IM groups), or received two IN vaccinations (IN-IN groups) in a 3-week interval (Figs. 1 and 2, Tables 1–3). Three different dose levels, low dose (LD), medium dose (MD), and high dose (HD), were evaluated. The distribution of participants by gender between the safety population groups did not show statistically significant differences according to the dose/route of administration. All the participants identified themselves as Mestizo. Regarding the distribution of patients according to age, there were no significant differences either between groups that received low, medium, or high doses by any administration routes. Average ages, the age range of participants, gender distribution, weight, height, and body mass index in each study group are indicated in Fig. 1C. Up to day 45 after the first vaccination, none of the enrolled individuals were excluded from the study for safety evaluation, but one subject had to be excluded from the immunogenicity evaluation due to a positive baseline titer, and several subjects had to be excluded due to SARS-CoV-2 infections (Table 4).

**Table 1:** Distribution of subjects in groups per dose and administration route/regimen.

Dose	Administration route 1st dose/2nd dose		
	IM/IM	IN/IN	IN/IM
10^7.0^ EID_50%_/dose (LD)	10	10	10
10^7.5^ EID _50%_/dose (MD)	10	10	10
10^8.0^ EID _50%_/dose (HD)	10	10	10

*IM* intramuscular, *IN* intranasal.

**Table 2:** Incremental dose administered per route/regimen for the first 18 subjects as a sentinel group for safety monitoring.

1st dose	Low dose (LD) (10^7.0^ EID_50%_/dose)			Medium dose (MD) (10^7.5^ EID _50%_/dose)			High dose (HD) (10^8.0^ EID _50%_/dose)		
	Day1	Day 2	Day3	Day 4	Day 5	Day 6	Day 7	Day 8	Day 9
IM	S1	S3	S5	S7	S9	S11	S13	S15	S17
IN	S2	S4	S6	S8	S10	S12	S14	S16	S18

Evaluation by an Independent Safety Committee 7 days after the last HD vaccination.

*IM* intramuscular, *IN* intranasal, *S* subject.

**Table 3:** Assignment of the first dose to subjects enrolled after the sentinel group.

1st dose	Low dose (LD) (10^7.0^ EID_50%_/dose)	Medium dose (MD) (10^7.5^ EID _50%_/dose)	High dose (HD) (10^8.0^ EID _50%_/dose)
IM	S19, S21, S23, S28, S30, S32, S37	S43, S45, S47, S52, S54, S56, S61	S67, S69, S71, S76, S78, S80, S85
IN	S20, S22, S24, S25, S26, S27, S29, S31, S33, S34, S35, S36, S38, S39, S40, S41, S42	S44, S46, S48, S49, S50, S51, S53, S55, S57, S58, S59, S60, S62, S63, S64, S65, S66	S68, S70, S72, S73, S74, S75, S77, S79, S81, S82, S83, S84, S86, S87, S88, S89, S90

Evaluation by an Independent Safety Committee 7 days after the last HD vaccination.

*IM* intramuscular, *IN* intranasal, *S* subject.

**Table 4:** Subjects infected by SARS-CoV-2 per group.

Dose	Route/regimen infected after 1st dose			Route/regimen infected after 2nd dose			Total per dose level
	IM/IM	IN/IN	IN/IM	IM/IM	IN/IN	IN/IM	
10^7.0^ EID_50%_/dose (LD)	1	0	0	0	0	0	1
10^7.5^ EID _50%_/dose (MD)	0	2	0	0	3	1	6
10^8.0^ EID _50%_/dose (HD)	1	1	0	0	0	1	4
Subtotal per route and administered doses	2	3	0	0	3	2	

*IM* intramuscular, *IN* intranasal.

### Safety of AVX/COVID-12-HEXAPRO

**Fig. 3:**
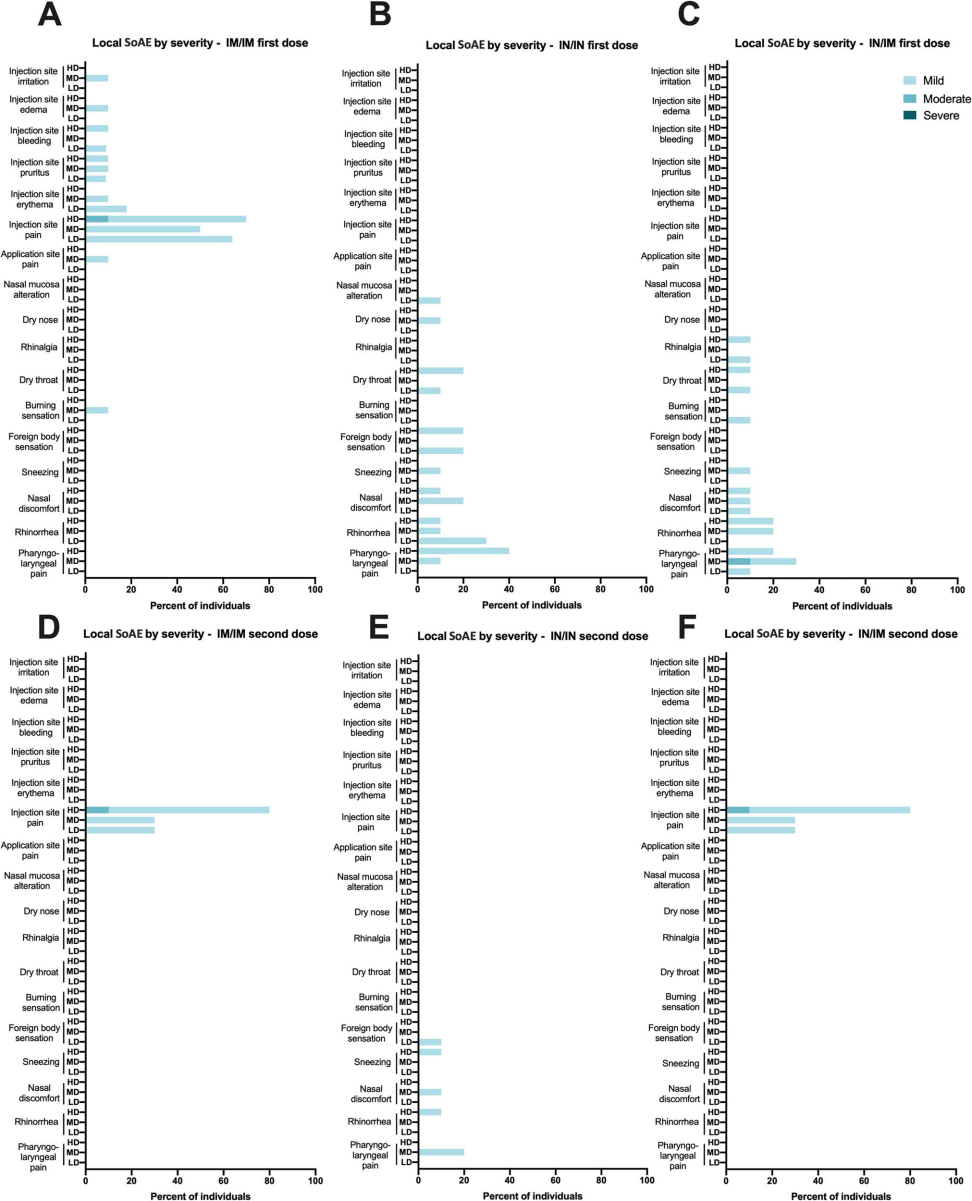
Overview of local Solicited Adverse Events. Frequency of local Solicited Adverse Events (SoAE) after vaccine application by route (IN or IM) for all study groups either after the first (**A**–**C**) or second (**D**–**F**) dose. LD low dose, MD medium dose, HD high dose.

**Fig. 4:**
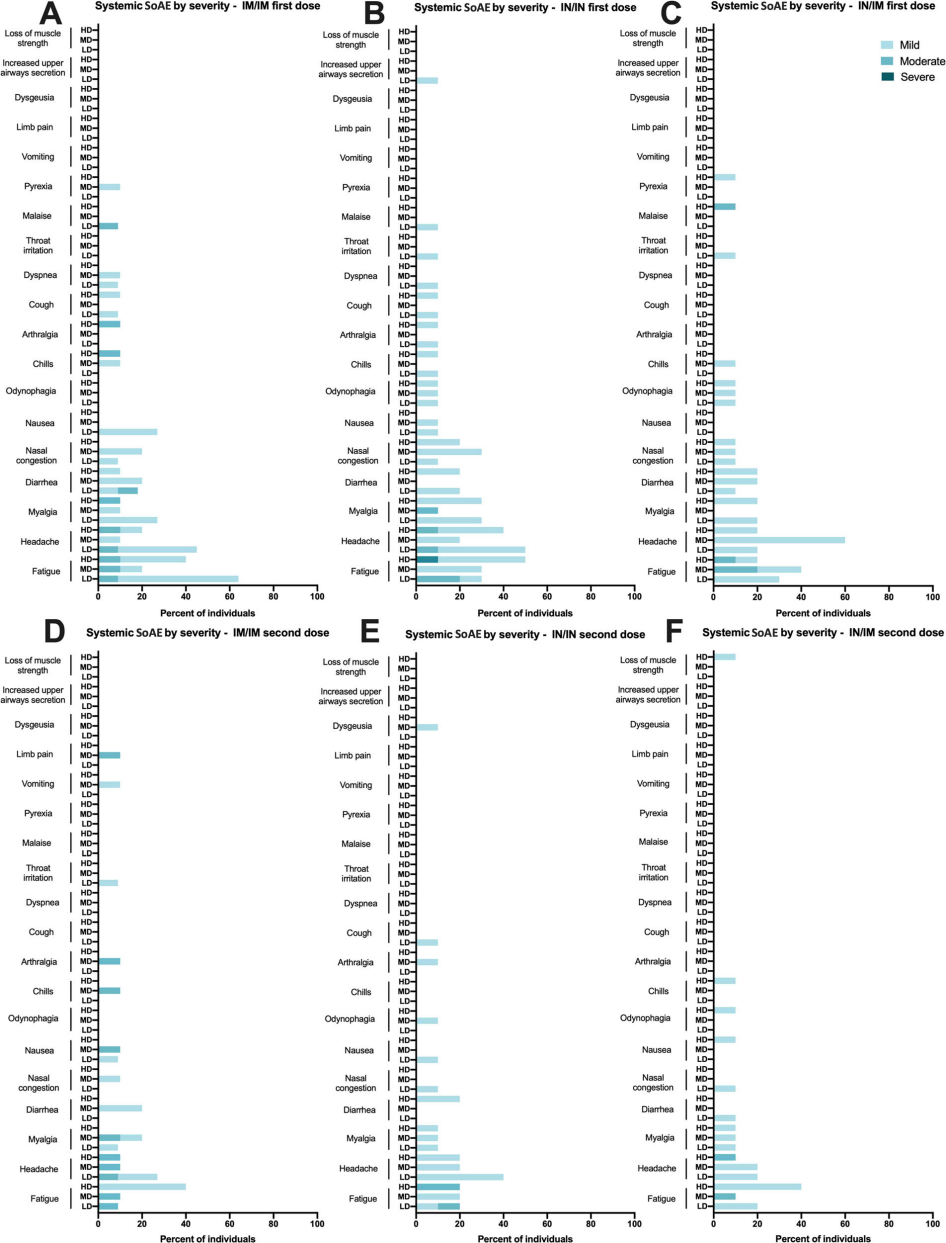
Overview of systemic Solicited Adverse Events. Frequency of systemic Solicited Adverse Events (SoAE) after vaccine application by route (IN or IM) for all study groups either after the first (**A**–**C**) or second (**D**–**F**) dose. LD low dose, MD medium dose, HD high dose.

In general, all formulations were well tolerated with little reactogenicity detected (Figs. 3 and 4, Supplementary Fig. 1). Up to day 45 after the first vaccination of the latest enrollment of a subject, there had been 625 adverse events (AEs in total, of which 319 occurred within the period considered for Solicited Adverse Events (SoAE, within 7 days after either of the two administrations). Of these 319 SoAE within the solicited period, 66 were considered local and 253 systemic. In general, the distribution of SoAEs among the different groups was not significantly different in the number of individuals or severity except for those of the IN route, who did not show the injection-related SoAEs, and those who received the vaccine IM who did not show nasal-related SoAEs. Also, no trend was detected regarding the type of systemic events per route or dose. As shown in Figs. 3 and 4, headache, fatigue, and myalgia were the most frequent mild SoAEs, and the type of systemic events did not change according to the route of administration and dose level, although a slightly lower number of AEs and SoAEs was observed in most groups after the second dose (Supplementary Fig. 1). None of the routes of administration or doses evaluated were associated with serious adverse events. Laboratory abnormalities accounted for approximately 1% of all registered adverse events.

Out of the 625 AEs, 552 (88.3%) were of mild intensity, 68 (10.9%) moderate, and only 5 (0.8%) severe intensity events were recorded (no severe SoAEs were recorded). The severe adverse events shown in Supplementary Fig. 1 occurred in a single individual for the IM/IM high dose, two individuals for the IN/IN high dose, and one individual for the IN/IM medium dose, all of them after the first dose. The events included dysmenorrhea, lethargy, abdominal pain, fatigue, and drowsiness. As mentioned above, no deaths or significant/serious adverse events were reported, and no alterations of vital signs or clinically significant events were reported.

### Immunogenicity of AVX/COVID-12-HEXAPRO

**Fig. 5:**
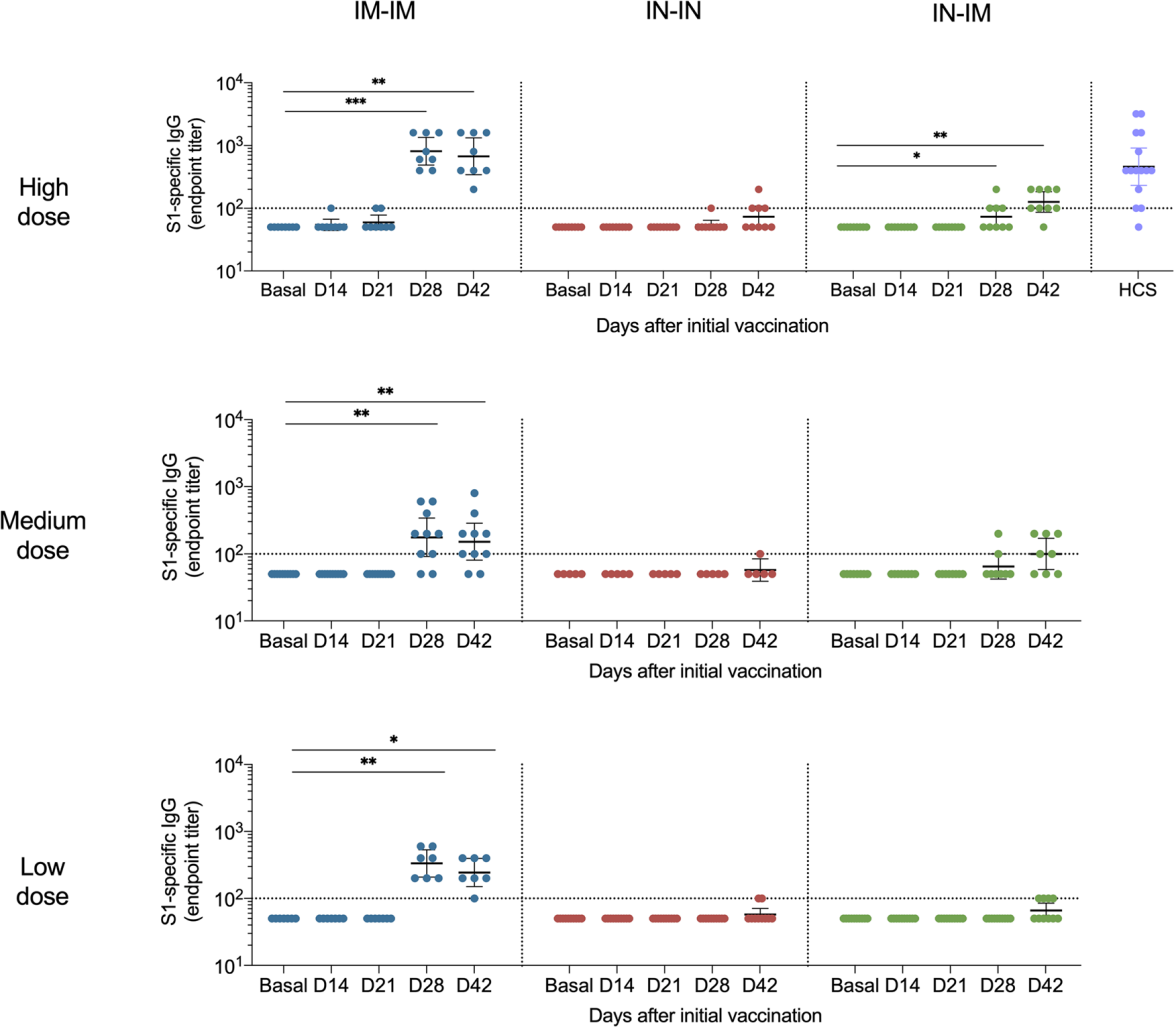
Spike-reactive antibody levels in sera from vaccinated volunteers. Antibodies against the S1 subunit of the spike protein (which contains the receptor binding domain (RBD)) were measured in vaccinees’ sera by ELISA at baseline and 14, 21, 28, and 42 days after the first vaccine dose administration. Individuals receiving the IM-IM regimen (left column), IN-IN regimen (middle column), or IN-IM (right column) with a high dose (top row), medium dose (middle row), or low dose (bottom row) of the vaccine are shown. Human convalescent serum (HCS) samples were added as additional controls. The limit of detection (LoD) is indicated by the horizontal dotted line. Bars show the geometric mean, and the error shows the 95% confidence intervals. Negative values are indicated as half of the LoD. Statistical significance is indicated as follows: *P < 0.05, **P < 0.01, ***P < 0.001, Friedman’s ANOVA followed by Dunn’s multiple comparison test.

To determine the immunogenicity of the vaccine at different dose levels and through different vaccination routes, we first performed enzyme-linked immunosorbent assays (ELISAs) against the S1 domain of the spike protein (Fig. 5). S1 was chosen as the target because this subdomain of the spike includes the N-terminal domain and the RBD, which hosts most of the neutralizing epitopes. In addition, a reliable commercial ELISA focusing on that target was available locally. For the IM-IM vaccination regimen, little induction of anti-S1 antibody was observed after the first dose. However, the second dose boosted titers to high reactivity in the HD group and somewhat lower reactivity in the MD and LD groups. As expected, the response after IN vaccination was lower and substantial reactivity was only detected in the HD group post-boost, with 56% of the individuals in the group having detectable titers. Finally, in the IN-IM regimen, the reactivity was similar to the IM-IM regimen, with an 89% response rate after the boost. Antibody titers induced by the HD IM-IM regimen were, in general, comparable to or higher than the titers of convalescent individuals.

**Fig. 6:**
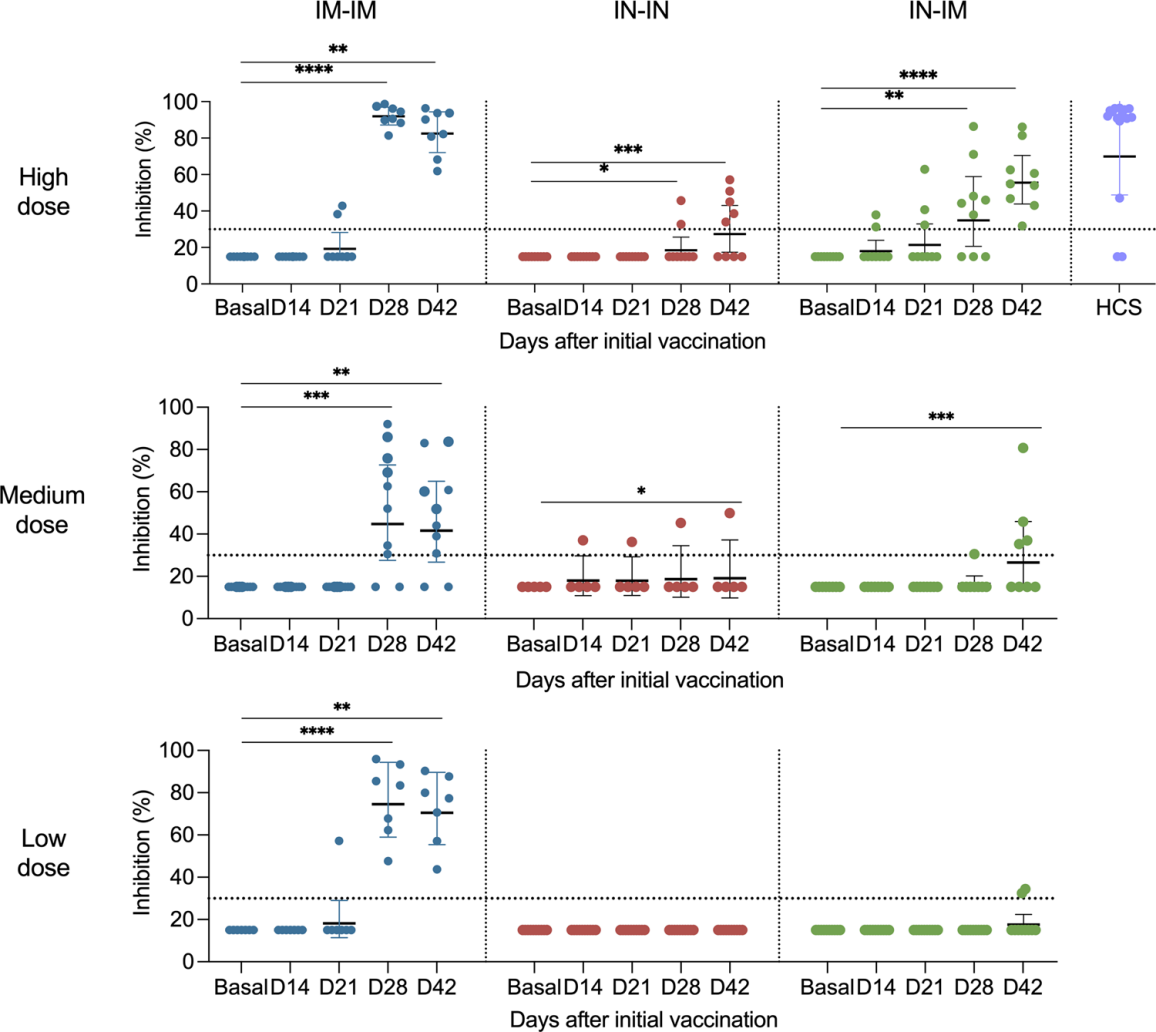
RBD–ACE2 interaction inhibiting antibodies in sera from vaccinated volunteers. Antibodies binding to the receptor binding domain (RBD) that inhibited its interaction with the angiotensin-converting enzyme 2 (ACE2) were assessed in vaccinees’ sera using an RBD–ACE2 interaction inhibition assay at baseline and 14, 21, 28, and 42 days after the first vaccine dose administration. Individuals receiving the IM-IM regimen (left column), IN-IN regimen (middle column), or IN-IM (right column) with a high dose (top row), medium dose (middle row), or low dose (bottom row) of the vaccine are shown. Human convalescent serum (HCS) samples were added as additional controls. The cutoff established for positivity (30%) in this assay is indicated by the horizontal dotted line. The cutoff was determined by the manufacturer, and that was validated with negative control and convalescent sera by INER. Bars show the geometric mean, and the error shows the 95% confidence intervals. Statistical significance is indicated as follows: *P < 0.05, **P < 0.01, ***P < 0.001, ****P < 0.0001, Friedman’s ANOVA followed by Dunn’s multiple comparison test.

While binding antibodies are important indicators of immunogenicity and have been correlated with protection^36,37^, we also wanted to determine functional antibody titers. A neutralization assay was unavailable for this interim analysis, but we performed a surrogate assay, which measures the inhibition of the interaction between the RBD and ACE2^38^. The titers detected in this assay do reflect results from the binding assay (Fig. 6). For the HD IM-IM group, the first vaccination increased the inhibitory titer just slightly in two subjects. However, strong inhibitory activity was observed at post-boost time points. This was also observed in the MD and LD groups, although more variability was detected there. For the IN-IN groups, little inhibitory activity was detected, and only in the HD group subjects. The IN-IM groups showed an intermediate phenotype, with all individuals in the HD group having post-boost inhibitory antibodies. The response rate in the MD group was lower, and only 20% of individuals in the LD group had activity above the limit of detection. Inhibition in the HD IM-IM regimen was, in general, comparable to inhibition of convalescent individuals. However, this assay does not allow us to determine differences between groups with very strong responses due to its limited dynamic range. It is therefore not possible to say if there were real differences between the HD IM-IM and the convalescent groups which had both titers at the upper limit of quantification. In summary, a high frequency of subjects responded with binding and inhibiting antibodies in the IM-IM regimens, while moderate to high frequencies were detected in the HD IN-IM group (Supplementary Fig. 2). As a caveat, the study was not designed to be powered to find differences between the groups, especially when the differences were small.

**Fig. 7:**
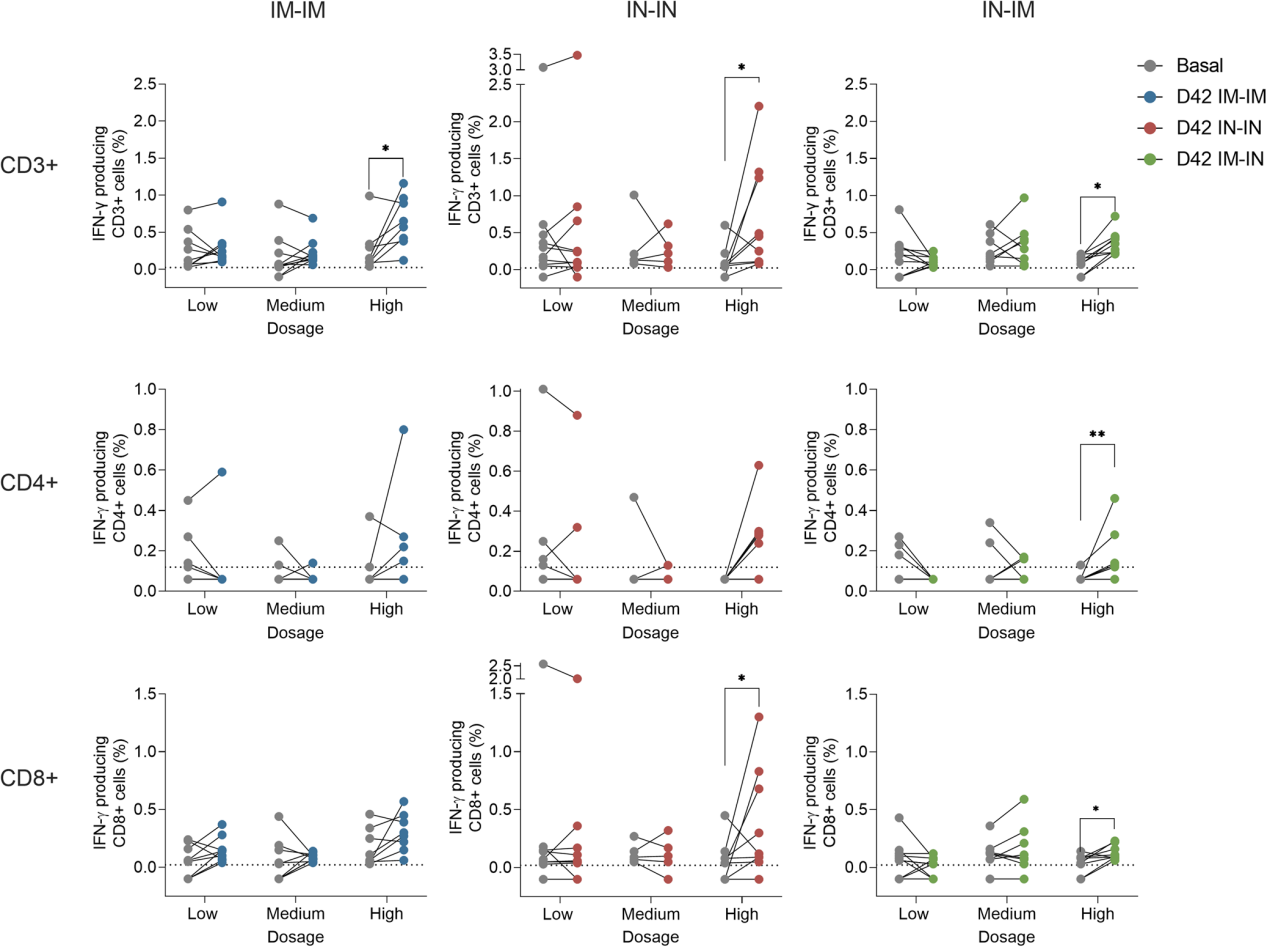
CD3+, CD4+, and CD8+ T-cell responses after vaccination. PBMCs were collected from vaccinees at baseline and 42 days after the first vaccine dose administration. Individuals receiving the IM-IM regimen (left column), IN-IN regimen (middle column), or IN-IM (right column) stratified by vaccine dose received (low, medium, or high) are shown. Activated CD3+ (top row), CD4+ (middle row), and CD8+ (bottom row) T-cells were determined by flow cytometry after 18-h incubation with recombinant spike protein. Frequencies of T-cells producing interferon-γ (IFN-γ) are presented. Statistical significance is indicated as follows: *P < 0.05, **P < 0.01, Wilcoxon’s signed rank test.

Cellular immune responses have been shown to be important for protection from SARS-CoV-2, especially when neutralizing antibody titers are low^39^. Here we assessed specific cellular immune responses by determining the percentage of CD3+, CD4+, and CD8+ cells that produced interferon-γ (IFN-γ) upon stimulation with the spike protein. Significant induction of IFN-γ-producing CD3+ cells was detected in all three HD vaccination regimens but not in the MD and LD groups when comparing day 42 with day 0 (Fig. 7). While a trend was seen for IFN-γ-producing CD4+ cells in the HD IM-IM and IN-IN groups, the increase was only statistically significant for the IN-IM HD group. No significant increases were found for CD4+ in the MD and LD groups. For CD8+ IFN-γ-producing cells, a trend was also observed for the IM-IM HD group, and the induction was significant for the HD IN-IN and IN-IM groups but not for any of the MD and LD groups. As a control of the specificity of the assay, a comparison of medium- vs spike-stimulated cells was performed (Supplementary Figs. 3, 4, and 5). Most of the participants had undetectable levels of activated CD3+, CD3+CD4+, and CD3+CD8+ T-cells upon stimulation with the medium.

As described above, breakthrough infections did happen during the first 42 days after vaccination. At day 42, there were 10 cases detected among groups, with no apparent trend dependent on dose or administration route (Table 4). The 10 cases were symptomatic, symptoms were mild, and none required hospitalization; 50% of the cases occurred before the second dose, and the other 50% of the cases occurred after the second dose.

Assessment of safety and immunogenicity will continue for 12 months, with sampling for immunogenicity planned at the 90-, 180-, and 365-day time points.

## Discussion

NDV-HXP-S can be produced at low cost and large scale using traditional egg-based influenza virus production processes. Influenza virus production capacity is available globally in high-income countries but also in LMICs^32^. In addition, veterinary vaccine producers often also have egg-based production capacity, which can be adapted for good manufacturing practice (GMP) production of human vaccines. The development of AVX/COVID-12-HEXAPRO could therefore alleviate the unmet global need for additional COVID-19 vaccine doses. Importantly, the superior spike antigen design of the NDV-vectored SARS-CoV-2 vaccine^31^ is an additional advantage. Furthermore, as demonstrated here, live NDV-vectored SARS-CoV-2 vaccine can be administered via the IN route. While assessment of mucosal immunity is not part of this interim report due to a lack of baseline samples and a lack of a properly qualified assay, intranasal vaccination is known to induce mucosal immunity that can potentially lead to sterilizing immunity and a complete block of transmission. Also, the favorable reactogenicity profile (as described here and in ref. ^34^), which is akin to that of influenza virus vaccines, makes the NDV-based vaccine likely more tolerable than mRNA or adenovirus-vectored vaccines^40–43^. As an example, for the mRNA-1273 vaccine^40^, a fever as high as 39.6 °C was reported after the second dose, whereas there were no reported cases for AVX/COVID-12-HEXAPRO. For mRNA-1273, moderate and severe systemic events reached 90%, at least for the medium and high doses after the second vaccination^40^ as compared to AVX/COVID-12-HEXAPRO where comparable systemic SoAEs were moderate only and observed at a maximum of 20%. Likewise, the reactivity of AVX/COVID-12-HEXAPRO is favorable when compared to ChAdOx1 nCoV-19^43^, where at least 50% of the systemic events were moderate or severe and fever of 38–39 °C was observed, including in individuals with paracetamol treatment.

Here we demonstrated that administration of live AVX/COVID-12-HEXAPRO is safe and well tolerated at all dose levels. However, only the HD vaccine regimen induced notable antibody and cellular immune responses when given via the IM-IM or IN-IM routes, comparable to those in convalescent individuals. Cellular immune responses were induced by the IN-IN route, but systemic antibody responses were not as robust. The low systemic response in naïve individuals after IN-IN administration was expected, and these results mirror those obtained with live AVX/COVID-12-HEXAPRO in the pig and rat models to some degree^33,35^. However, we would expect that IN administration as a booster dose in individuals who received regular vaccination regimens in the past would be more effective, and we would also expect that strong mucosal immune responses may, in fact, be highly efficacious in protecting against infection and transmission. Unfortunately, in this interim report, due to a lack of baseline samples and lack of a properly qualified assay for secretory IgA at the site, these mucosal IgA titers could not be assessed. Given the robust immunogenicity and the high tolerability of the HD IM-IM and IN-IM vaccination regimens, it is justified to further develop these two modalities in Phase II trials. The IN-IM regimen is especially attractive since it likely induces both systemic and mucosal immunity. However, its implementation may be more complex since two formulations have to be used and administered via two different routes. Nevertheless, it may be worth implementing this strategy in populations who are still naïve, e.g., in children. Importantly, given the high seroprevalence for SARS-CoV-2 in many regions globally and given the need for booster doses in a part of the population (elderly, immunosuppressed, health care workers, etc.), the HD vaccination regimens should certainly also be evaluated in individuals with pre-existing immunity, likely as single IM or IN administrations. Currently, a single-dose booster trial is ongoing in Mexico City based on the data reported here with one IM or IN HD vaccination, and a phase II/III study based on the HD IM scheme has also started in Mexico with the phase III part now being fully enrolled.

Our study has several limitations. Quantitative neutralization assays with authentic SARS-CoV-2 could not be performed at the study site at the time of analysis due to biosafety restrictions. In addition, in this interim analysis, neutralizing activity against variants of concern could not be assessed. Another aspect that was not assessed is viral shedding and stability of the S gene in vivo as well as anti-vector immunity induced. These aspects will be explored in a parallel, ongoing study in the US (NCT05181709). However, based on data from our experiments in pigs and non-human primate data in the literature, we expect minimal shedding and no transmission of the vaccine virus to others^33,44^. Furthermore, we were not able to directly compare immune responses induced by the live NDV-HXP-S to those induced by inactivated NDV-vectored SARS-CoV-2 vaccines^34^ or those observed following administration of other authorized/licensed COVID-19 vaccines. We expect to perform these additional assays and direct comparisons at later time points as soon as reagents and materials become available. So far, the assessment of mucosal antibodies has also not been possible. Finally, this was a non-randomized open-label study without a placebo control group, which is more prone to biases as compared to randomized and double-blinded study designs.

In conclusion, we show that the live AVX/COVID-12-HEXAPRO vaccine has a safety profile that is remarkably independent of the dose and administration route with low frequency and intensity. Furthermore, the HD IM-IM and IN-IM vaccination regimens showed strong evidence of immunogenicity, warranting further development of this vaccine candidate. Finally, it is important to note that the NDV vector technology is amenable to rapid changes in antigens expressed, allowing for strain changes to match emerging viral variants. Beta-, Delta- and Gamma-specific versions of NDV-HXP-S have already been tested pre-clinically^45^, and BA.1, BA.2, BA.5, BQ.1.1 and XBB.1.5 versions have also been developed.

## Methods

### Study design and participants

The phase I study (clinicaltrials.gov #NCT04871737) was designed to evaluate the safety and immunogenicity of NDV-HXP-S given via three different vaccination strategies: intramuscular vaccination on day 0 and day 21, intranasal vaccination on day 0 followed by intramuscular vaccination on day 21, and intranasal vaccination on day 0 and day 21. In addition, three different dose levels were tested, 10^7.0^–10^7.49^ 50% egg infectious doses (EID_50,_ low dose (LD)), 10^7.5^–10^7.99^ EID_50_ (medium dose, MD), and 10^8.0^–10^8.49^ EID_50_ (high dose, HD), resulting in nine groups with 10 participants each (Table 1). Female and male participants between 18 and 55 years of age without prior immunity to SARS-CoV-2 were enrolled. The protocol was designed by ProcliniQ Investigación Clínica, S. A. de C. V. with input from the Instituto Mexicano del Seguro Social (IMSS) and Laboratorio Avi-Mex, S. A. de C. V. (Avimex®), the latter as a sponsor with the statistical help of iLS Clinical Research, S. C. The study was approved by the Federal Commission for the Protection against Sanitary Risks (COFEPRIS) in Mexico under number 213300410A0063/2021, after approval by the Ethics, Biosafety and Research Committees of the clinical research site Hospital Medica Sur (03-2021-CI/CEI/CB-156) in full compliance of the Mexican regulation and under the principles of the Declaration of Helsinki and Good Clinical Practice. The samples for the immunological assays were processed at the National Institute for Respiratory Diseases (INER) in Mexico City.

The primary outcomes were to evaluate the safety of the three concentrations (viral titers) and three administration routes across nine groups. Immunogenicity measurements, including the induction of IgM and IgG, neutralizing antibodies, cellular responses, and induction of mucosal immunity (mucosal IgA, neutralizing IgA), were secondary outcomes.

The study inclusion criteria were: 18- to 55-year-old adults; no respiratory disease within the last 21 days prior to first dose administration; a body mass index between 18.0 and 29.0 kg/m^2^ (inclusive); negative RT-PCR for SARS-CoV-2 infection; negative test for anti-SARS-CoV-2 antibodies; O_2_ saturation ≥92% by pulse oximetry; normal CT scan of thorax; no symptoms from clinical history and normal physical exam at screening visit; laboratory test values within normal ranges for urinalysis, liver enzymes, renal function tests, cholesterol and triglycerides, fasting glucose, and hematology; negative test for HBsAg, anti-HCV and anti-HIV-1 antibodies; negative VDRL test; normal electrocardiogram; negative pregnancy test for women of childbearing potential; agreement of all sexually active volunteers to use highly effective contraceptives over the study period and up to 30 days after the last administration of the experimental vaccine; and commitment from all participants to keep social distancing, use of mask, and frequent hand washing with soap or antibacterial gel during the study period. Exceptions to limits in laboratory determinations were authorized as per the investigator’s judgment.

Exclusion criteria included a history of hypersensitivity or allergy to any ingredient of the vaccine; a history of severe anaphylactic reaction; a history of seizures; a history of chronic diseases or cancer; vaccination against SARS-CoV-2 with approved or experimental vaccines; participation in any other study with an experimental intervention within the last 3 months; administration of any other drug or herbal preparation within the last 30 days; any vaccine administered within the last 30 days, including influenza vaccine; fever at screening; blood transfusion or blood components transfusion within the last 4 months; regular activity related to work, social interaction, or entertainment that represents an exposure risk to SARS-CoV-2 higher than that of the general population, as per investigator judgment; drug and alcohol abuse; any medical or non-medical condition that could interfere with patient safety and study compliance or data interpretation, as per investigator judgment.

### Study groups

This phase I study was designed as a non-randomized open-label study without a placebo control group. Ninety volunteers were assigned to one of nine treatment groups in the order of enrollment according to Table 1. The first intervention of each treatment group was made sequentially to 18 sentinel subjects, according to Table 2. The first 18 subjects (S1–S18) received a dose incrementally from the lowest to the highest viral titer with no more than one subject per day, per dosage and route of administration. The safety data of the sentinel subjects were then evaluated by an independent Safety Data Monitoring Committee (SDMC) before authorizing the administration of the first vaccine dose to the rest of the subjects, who were then sequentially enrolled, according to Table 3. The SDMC also evaluated the safety data of the full cohort after the first dose before the administration of the second dose to the 19th subject enrolled (first outside the sentinel group) on day 21 after the first dose.

There was a deviation with one of the subjects who reported negative results in the PCR and IgG/IgM tests for SARS-CoV-2 at screening and who was therefore enrolled in the clinical trial and received the first intramuscular vaccine dose (Day 0) in the low dose (LD) group. However, a subsequent testing of anti-spike antibodies, post-administration of the vaccine, showed a low yet positive antibody level (148.8 AU/mL, Elecsys® Anti-SARS-CoV-2 S, Roche Diagnostics). The investigators reviewed the case and considered that it was in the best interest of the subject to remain in the study since the safety of the subject was not at risk and vaccination for the age group to which the subject belongs was at the time of the study not available under the Mexican national vaccination program. This decision would also be consistent with an ethical obligation to properly monitor the safety of the volunteer. The subject consented to continue participation in the study with the sponsor’s authorization. Safety data were included in the safety analysis, but immunogenicity data from this subject was not considered for immunogenicity assessment.

For those subjects who received the first dose intranasally (IN), the second dose was administered by alternating the administration route. The first subject was given the second dose via the IN route, followed by the second subject, who was dosed by the IM route. This alternation continued until the IN/IN and IN/IM groups were dosed at each dose level, according to Table 1. All subjects who received the first dose via IM also received the second dose via IM in order to complete the corresponding IM/IM groups.

As an additional circumstance around the protocol, it is important to stress that the study was conducted almost concurrently with a COVID-19 wave in Mexico driven by the emergence of the B.1.617.2 (Delta) variant in Mexico City^46^. This circumstance affected the clinical trial as some of the participants were infected either between the first and the second dose or after the administration of the second dose, as reported in Table 4.

According to the above, the total *N* for safety assessment was 91 participants, and for immunogenicity assessments, the *N* was variable per group since subjects who acquired an infection (see Table 4) were excluded from analyses, and their data are not included in the immune analysis figures.

### Procedures

As mentioned above, AVX/COVID-12-HEXAPRO (Patria) is a Newcastle disease virus (NDV)-based SARS-CoV-2 vaccine based on the LaSota vaccine strain of NDV^28–30^. It was engineered to express a version of the spike protein of SARS-CoV-2, which includes six proline mutations and a deletion of the polybasic cleavage site, keeping the spike in a stable pre-fusion conformation^31^. In addition, the ectodomain of the SARS-CoV-2 spike was grafted onto the transmembrane and cytoplasmic domains of the NDV fusion protein to ensure optimal incorporation into the Newcastle disease virion. The vaccine was obtained as reported^28–30,33^ and manufactured under Good Manufacturing Practices at the COFEPRIS-approved facilities of Laboratorio Avi-Mex, S. A. de C. V. in Mexico City. The vaccine was formulated without adjuvants in three different viral titers per dose (LD, MD, HD), as described above. For the intramuscular (IM) administration, it was formulated in single-dose vials with the corresponding viral titer contained in 0.5 mL for administration as a single injection into the deltoid muscle. In the case of the intranasal (IN) administration, it was formulated in single-dose vials as a 0.2 mL solution containing the corresponding viral titer for the administration of 0.1 mL in each nostril. The vaccines formulated as described were stored under refrigeration (4 °C).

The 0.5 mL intramuscular dose was administered through a regular syringe and needle, and for the 0.2 mL intranasal route, a nasal sprayer device coupled to the syringe (MAD Nasal™ Intranasal Mucosal Atomization Device) was used instead.

The study was conducted at Hospital Medica Sur in Mexico City. Written informed consent was obtained from each participant, as approved by COFEPRIS, to voluntarily participate in the study for 12 months, including 11 visits to the site plus at least six telephone follow-up calls scheduled according to the date of the first visit.

A screening visit was conducted 3 days before vaccination, where each participant underwent a full medical history and examination. A medical history was obtained, including a recording of all vaccines and medications received within the last 30 days and daily activities that posed a high risk of getting infected with SARS-CoV-2. The physical examination included measurement of vital signs (blood pressure, heart rate, respiratory rate, and temperature), oxygen saturation, weight, and height. At the screening visit, participants were also subject to urine and blood testing, hematology, blood cell count, kidney and liver function test, blood lipids, and testing for human immunodeficiency virus (HIV), hepatitis B and C virus and syphilis, pregnancy tests for women of childbearing potential, electrocardiogram, and thorax CT scan. In addition, the participants were subject to COVID-19 testing (nucleic acid-GeneFinder^TM^ COVID-19 Plus RealAmpKit, and antibody, as above) to exclude prior or active infection, as such infection was part of the exclusion criteria. Further details on eligibility are provided in the trial protocol (Supplementary Appendix 1).

Eligible subjects were enrolled and were administered the first vaccine dose corresponding to their group and given a patient diary at basal visit (D0). Vital signs were measured prior to the administration of each dose and at 90 min thereafter. Subjects were observed on-site during that period. Further daily telephone interviews were conducted from days 1 to 6 for collection of safety data, and participants returned to the site on day 7 (D7) after the first dose administration (D0), followed by scheduled visits on days 14, 21, 28, 42, 90, 180, and 365. All on-site visits included measurement of vital signs, weight, and determination of body mass index (BMI). Visits from days 7 to 90 included safety labs, and abnormalities were reported as adverse events according to their nature. Data for visits on days 90, 180, and 365 are not yet available since the trial is still ongoing.

Day 14, 21, 28, 42, 90, 180, and 365 visits include blood sampling for IgM–IgG–IgA antibodies, neutralizing antibodies, and T-cell responses. In addition, those subjects who received at least one IN dose also provided saliva and nasal swab samples on these same dates. According to the study protocol, basal samples of saliva and nasal fluids were not collected as there was no previous infection, and specific antibodies were likely negative.

A PCR test was also performed prior to the application of the second dose of AVX/COVID-12-HEXAPRO. As described above, participants positive for SARS-CoV-2 infection were considered for the safety assessment but excluded from the immunogenicity analysis.

Adverse events were documented based on standardized terms (MedDRA) and classified as Adverse Events (AE) and Serious Adverse Events (SAE), both as defined by ICH/E6R2 Good Clinical Practices definitions. Solicited Adverse Events (SoAE) were defined in the protocol as those that appeared within 7 days from vaccination and categorized as “local” when related to the injection or the intranasal administration (inflammation, redness, local increased temperature, itching, low-grade fever) or “systemic” or related to COVID-19 disease (fever, chills, cough, difficult breathing, muscular or articular pain, headache, anosmia, ageusia, odynophagia, nasal congestion or secretion, nausea or vomiting, diarrhea or fatigue).

The number and percentage of AEs, SAEs, and SoAEs were recorded after every vaccine administration. SoAEs were considered associated with vaccination and evaluated 7 days after each vaccination, while AEs were assessed after 21 days of vaccination. AE intensity was generally registered as low, mild, or severe according to the protocol. Clinically relevant abnormalities in laboratory tests or at the physical examination were recorded by groups and then correlated to the vaccine dose and administration route.

### Sample collection

Blood samples, nasal exudates, and saliva samples were obtained as described above, according to the group, at the clinical research site and transported to INER at room temperature. Blood samples were processed within two and a half hours of vein puncture. Biological samples were obtained before and 14, 21, 28, and 42 days after the first vaccination.

Venous blood was obtained using standard procedures and was collected into separator tubes (SST BD vacutainer tubes, Franklin Lakes, NJ, USA). Vacutainer tubes were centrifuged at 620×*g* (centrifuge: Rotanta 460R; Rotor: 5624, Hettich, Tuttlingen DE) for 10 min to separate serum. The serum was then removed from the upper portion of the tube, aliquoted, and stored at −20 °C until use.

Serum samples from convalescent individuals (*N* = 51, collected at a median of 41 days post onset (standard deviation 12 days, range 21–65 days)) with SARS-CoV-2 infection confirmed by real-time reverse transcription PCR (RT-PCR) were collected after recovery on the day they resumed regular activities to be used as positive controls for validation of the techniques and serum samples from healthy individuals obtained between 2014 and 2018 (prepandemic) were used as negative controls for validation of the techniques only (INER approved protocol number B20-21 and B22-12). Convalescent serum samples were collected from individuals with mild to moderate illness who had any of the various signs and symptoms of COVID-19 (fever, cough, sore throat, headache, muscle pain, loss of taste and smell, dyspnea or abnormal chest imaging) and evidence of lower respiratory disease during clinical assessment. Fifteen of the 51 samples of which enough volume was available were used for titer comparison.

All blood samples and blood products, nasal exudates, and saliva were handled in a BSL-2 laboratory with the use of appropriate personal protective equipment and safety precautions using processing protocols approved by the INER Institutional Biosafety Committee.

Venous blood was collected in sodium heparin tubes (BD vacutainer tubes, Franklin Lakes, NJ, USA) and diluted 1:1 within two and half hours for whole blood stimulation with 0.99 μg/mL of S1 subunit of the spike protein (RayBiotech, Peachtree Corners, GA) in the presence of anti-CD28/CD49d (BD, San Jose CA) for 18 h 20 min at 37 °C in 5% CO_2_.

### Enzyme-linked immunosorbent assay (ELISA)

S1-specific IgG in serum samples was measured using two commercial kits from EuroImmun, following the manufacturer’s instructions and using an analyzer (Euroimmun AG, Lübeck, Germany). Serum samples were diluted 1:100, and 100 μL of samples, calibrator, negative and positive control were added to each well and incubated at 37 °C for 60 min. This step was followed by three washes using 300 μL of washing buffer per well. Then, 100 μL of the anti-human IgG, labeled with peroxidase, was added and incubated at 37 °C for 30 min for IgG detection. The plates were subsequently washed before the addition of 100 μL of substrate solution. After incubation for 30 min at room temperature, 100 μL of stop solution was added, and the optical density (OD) was read at 450 nm in the analyzer (EuroImmun) within 30 min after adding the stop solution. The results were reported as the ratio between the extinction of the sample and the extinction of the calibrator, and a ratio of >1.1 was considered positive^47^. For serum analysis, twofold serial dilutions were processed as described above, and the endpoint titer was calculated as the most diluted serum concentration that gave a ratio >1.1. The limit of detection was 1:100; samples with activity below the limit of detection were assigned a titer of 1:50 for graphing purposes. Samples ran across multiple plates were calibrated using a manufacturer-provided calibrator solution.

### RBD–ACE2 interaction inhibition assay (RAIIA)

To determine the presence of antibodies that block the interaction between the spike receptor binding domain (RBD) and the angiotensin-converting enzyme 2 (ACE2) receptor, we used a receptor binding domain (RBD)–angiotensin-converting enzyme 2 (ACE2) interaction inhibition assay (RAIIA). The setup used was a commercial assay from GenScript, which is a protein-based surrogate neutralization assay^38^. Samples were analyzed following the manufacturer’s instructions (GenScript version RUO 3.0 update 01/02/2021). Briefly, samples and controls were diluted 1:10 in kit sample buffer and mixed 1:1 with horseradish peroxidase (HRP)-conjugated recombinant SARS-CoV-2 RBD fragment (HRP-RBD) and incubated at 37 °C for 30 min to allow binding of circulating antibodies to HRP-RBD. The mixture was then added to the capture plate, which was pre-coated with the human ACE2 protein. Unbound HRP-RBD, as well as any HRP-RBD bound to non-neutralizing antibody, was captured on the plate while circulating neutralization antibody-HRP-RBD complexes remained in the supernatant and get removed during washing. Then 3,3’,5,5’-tetramethylbenzidine (TMB) solution was added. By adding the stop solution, the reaction was quenched, and the plates were read at 450 nm using Analyzer 1 (EuroImmun). The absorbance of a sample is inversely correlated with blocking RBD–ACE2 interactions. The results are expressed as the percentage (%) of inhibition, and 30% inhibition was used as cutoff^38^.

### Intracellular cytokine staining assay

Whole blood diluted 1:1 was stimulated with Roswell Park Memorial Institute (RPMI) 1640 medium (Lonza, Walkersville, MD) with 0.99 μg/mL of S1 subunit of spike protein (RayBiotech, Peachtree Corners, GA) in the presence of anti-CD28/CD49d (BD, San Jose CA) for 18 h 20 min at 37 °C in 5% CO_2_. GolgiStop (BD, San José, CA) was added, and the samples were cultured additionally for 4 h. This recombinant S1 protein was chosen from three different spike proteins tested in preliminary experiments; overlapping peptides were not available at the local site. The medium was used as a negative control, and 10 μg/mL of phytohemagglutinin (PHA, Sigma-Aldrich) as a positive control. Samples were washed with phosphate-buffered saline (PBS) without Ca^2+^ and Mg^2+^ (Lonza) and stained with Live/Dead near-IR Dead Cell Stain Kit for 633 or 635 nm excitation (Invitrogen, Eugene, OR) for 15 min at room temperature in the dark. Then red blood cells (RBCs) were lysed with RBC lysis buffer (BD) for 10 min, followed by a washing step with staining buffer PBS without Ca^2^^+^ and Mg^2^^+^ supplemented with 1% fetal bovine serum (FBS) and 0.1% NaN_3_. Cell surface staining was performed using a cocktail of anti-human CD3, CD4, and CD8 antibodies in staining buffer for 15 min at room temperature in the dark. After an additional washing step with staining buffer, the cells were fixed and further permeabilized using BD Cytofix/Cytoperm following the manufacturer’s instructions. Intracellular staining was performed in Cytoperm using anti-human IFN-γ antibodies for 30 min at room temperature in the dark. Cells were washed with BD Perm/Wash buffer and further resuspended in PBS. Cells were kept at 4 °C in the dark until acquisition and analysis. Unstained and fluorescence minus one (FMO) controls were included. Details of the antibodies used in the flow cytometry assay are listed in Supplementary Table 1, and the rest of the reagents are listed in Supplementary Table 2. At least 200,000 events of the lymphocyte region in a forward scatter (FSC) vs side scatter (SSC) scatter plot were acquired in a fluorescence-activated cell sorter (FACS) Aria II (BD). Analysis was performed using FACS Diva 8.0. The gates applied for the identification of SARS-CoV-2 antigen-specific cytokine-producing CD3+, CD4+, or CD8+ cells were defined using the FMO controls and used for the limit of detection (LOD)^48^. A positive response in this assay is defined as a statistically significant difference between time points.

### Primary outcomes

Primary outcomes of the study were established as follows:

- To evaluate the safety of three concentrations (10^7.0–7.49^, 10^7.5–7.99^, 10^8.0–8.49^ EID_50%_/dose) of the recombinant vaccine against SARS-CoV-2 based on a Newcastle disease virus (rNDV) administered twice intramuscularly, twice intranasally, or intranasally followed by intramuscularly in healthy volunteers

### Secondary outcomes

- To evaluate the immunogenicity of three concentrations (10^7.0–7.49^, 10^7.5–7.99^, 10^8.0–8.49^ EID_50%_/dose) of the recombinant vaccine against SARS-CoV-2 based on a Newcastle disease virus (rNDV) administered twice intramuscularly, twice intranasally, or intranasally followed by intramuscularly in healthy volunteers
- To evaluate the nasal mucosal humoral immunity of three concentrations (10^7.0–7.49^, 10^7.5–7.99^, 10^8.0–8.49^ EID_50%_/dose) of the recombinant vaccine against SARS-CoV-2 based on a Newcastle disease virus (rNDV)

This manuscript describes an interim analysis that focuses on initial safety data and binding and ACE2/RBD interaction inhibiting antibodies and T-cell-based immunity. Other readouts will be described in future publications.

### Statistical analysis

Interim analyses were scheduled for days 21, 28, 42, and after 6 and 12 months (end of study). This report includes data obtained up to day 42. For continuous variables, one-way ANOVA and Student’s *t*-test were used, and non-parametric tests were used for discrete (count) variables. Safety endpoints were expressed as frequencies (%). In this report, all analyses are descriptive only, as samples are still pending further analyses, and the results reported here are preliminary in nature. IgG titers are reported per group as geometric mean titers (GMT) with a 95% CI at days 0 (basal), 14, 21, 28, and 42. For logarithmically transformed antibody titers, Friedman’s test or Wilcoxon’s rank-sum test were used for non-normally distributed data, and significances between groups were paired and differences assessed with a 95% CI. The proportion of subjects with a titer above a predetermined parameter for IgG, IgM, and IgA with 95% CI at days 14, 21, 28, 42, 90, and 180, as well as 12 months, will also be analyzed after completion of the study. Seroconversion rate with respect to the basal titer was also determined as the proportion of subjects with detected titers of specific antibodies for the spike protein of SARS-CoV-2 as determined by ELISA and for assessing the capacity of circulating antibodies to inhibit the interaction between RBD and ACE2. T-cell-mediated responses were assessed as a proportion of positive responders. The full statistical analysis details are provided in the trial protocol (Supplementary Appendix 1) and will be performed in full upon completion of all procedures.

### Role of funding source

The funding for the clinical study was provided by the National Council for Science and Technology (CONACYT, México), except for all the production and vaccine product supply, which was funded solely by Avimex. CONACYT did not participate in the trial design but did evaluate it and approved the project through their National Committee on Research, Development and Innovation on Public Health. Funding was managed by Avimex and used to pay for all laboratory tests, clinical sites, and clinical professionals. CONACYT also facilitated the identification, purchase, and importation of certain supplies and the communication with other entities of the Federal Mexican Government to facilitate the study.

### Sample availability

Samples tested in this study are unique clinical trial samples of limited volume and cannot be shared.

### Reporting summary

Further information on research design is available in the Nature Research Reporting Summary linked to this article.

## Supplementary information

Supplementary material

REPORTING SUMMARY

Supplementary Appendix 1

## Data Availability

The data that support the findings of this study are available from the corresponding author upon reasonable request.
