# Supplementary material for: Interim safety and immunogenicity results from an NDV-based COVID-19 vaccine phase I trial in Mexico
Source: NPJ Vaccines. 2023 May 10;8:67. doi: 10.1038/s41541-023-00662-6 (PMC10170424; doi:10.1038/s41541-023-00662-6)
Supplement: Supplementary file 1 — Supplementary material [file 41541_2023_662_MOESM1_ESM.pdf]

Supplementary Data for:

Interim safety and immunogenicity results from an NDV-based COVID-19 vaccine phase I trial in Mexico

Samuel Ponce-de-León[2], Martha Torres[3], Luis Enrique Soto-Ramírez[4,6], Juan José Calva[4], Patricio Santillán-Doherty[5], Dora Eugenia Carranza-Salazar[7], Juan Manuel Carreño[8], Claudia Carranza[3], Esmeralda Juárez[9], Laura E. Carreto-Binaghi[3], Luis Ramírez-Martínez[1], Georgina Paz-De la Rosa[1], Rosalía Vigueras-Moreno[1], Alejandro Ortiz-Stern[14], Yolanda López-Vidal[12], Alejandro E. Macías[11], Jesús Torres-Flores[10], Oscar Rojas-Martínez[1], Alejandro Suárez-Martínez[1], Gustavo Peralta-Sánchez[1], Hisaaki Kawabata[8], Irene González-Domínguez[8], José Luis Martínez-Guevara[8], Weina Sun[8], David Sarfati-Mizrahi[1], Ernesto Soto-Priante[1], Héctor Elías Chagoya-Cortés[15], Constantino López-Macías[13], Felipa Castro-Peralta[1], Peter Palese[8,16], Adolfo García-Sastre[8,16,17,18,19,#], Florian Krammer[8,19,#] and Bernardo Lozano-Dubernard[1,#]

[1]*Laboratorio Avi-Mex, S. A. de C. V. (Avimex), Maíz 18, Granjas Esmeralda, CP 09810, Iztapalapa, CDMX, Mexico.*
[2] *Programa Universitario de Investigación en Salud (PUIS)., Facultad de Medicina, Universidad Nacional Autónoma de México (UNAM), Edif. de los Programas Universitarios, Planta Alta. Circuito de la Investigación Científica S/N Ciudad Universitaria, Ciudad de México, C.P. 04510. México.*
[3]*Laboratorio de Inmunobiología de la tuberculosis, Instituto Nacional de Enfermedades Respiratorias (INER), Calzada de Tlalpan 4502, Sección XVI, CP 14080, Tlalpan, México.*
[4]*Department of Infectious Diseases, Instituto Nacional de Ciencias Médicas y Nutrición "Salvador Zubirán", Vasco de Quiroga 15, Belisario Dominguez, Sección XVI, 14080, Tlalpan, México*
[5]*Instituto Nacional de Enfermedades Respiratorias (INER), Calzada de Tlalpan 4502, Sección XVI, CP 14080, Tlalpan, México.*
[6]*Departamento de Infectología y Vigilancia Epidemiológica, Hospital Médica Sur, S.A.B. de C. V., Puente de Piedra 150, Toriello Guerra, 14050, Tlalpan, México.*
[7]*ProcliniQ Investigación Clínica, S. A. de C. V. , Renato Leduc 155 (Xontepec 91), Toriello Guerra, 14050, Tlalpan, México.*
[8]*Department of Microbiology, Icahn School of Medicine at Mount Sinai, 1 Gustave L. Levy Pl, New York, NY 10029, USA*
[9]*Departamento de Investigación en Microbiología, Instituto Nacional de Enfermedades Respiratorias (INER), Calzada de Tlalpan 4502, Sección XVI, CP 14080, Tlalpan, México..*
[10]*Dirección Adjunta de Desarrollo Tecnológico, Vinculación e Innovación, Consejo Nacional de Ciencia y Tecnología (CONACYT), Insurgentes Sur 1582, Crédito Constructor, CP 03940, Benito Juárez, CDMX*
[11]*Departamento de Medicina, Universidad de Guanajuato, 20 de Enero 929, C.P 37000, León Guanajuato. México*
[12]*Programa de Inmunología Molecular Microbiana, Departamento de Microbiología y Parasitología, Facultad de Medicina, Universidad Nacional Autónoma de México (UNAM), Av. Universidad 3000, Circuito Interior S/N. Ciudad Universitaria. Coyoacán. CP.04510. México.*
[13]*Unidad de Investigación Médica en Inmunoquímica. Hospital de Especialidades del Centro Médico Nacional Siglo XXI. Instituto Mexicano del Seguro Social (IMSS), Av. Cuauhtémoc 330, Doctores, C.P. 06720,CDMX, México.*
[14]*iLS Clinical Research, S. C. (iLS), Matias Romero 102 - 205 Del Valle, Benito Juárez, CP 03100, CDMX, México*
[15]*Consultora Mextrategy, S.A.S. de C. V. (Mextrategy), Insurgentes Sur 1079 P7-127, Nochebuena, CP 03720, CDMX, Mexico*
[16]*Department of Medicine, Icahn School of Medicine at Mount Sinai, 1 Gustave L. Levy Pl, New York, NY 10029, USA*
[17]*Global Health and Emerging Pathogens Institute, Icahn School of Medicine at Mount Sinai, 1 Gustave L. Levy Pl, New York, NY 10029, USA*
[18]*The Tisch Cancer Institute, Icahn School of Medicine at Mount Sinai, 1 Gustave L. Levy Pl, New York, NY 10029, USA*
[19]*Department of Pathology, Molecular and Cell based Medicine, Icahn School of Medicine at Mount Sinai, 1 Gustave L. Levy Pl, New York, NY 10029, USA*

#To whom correspondence should be addressed: florian.krammer@mssm.edu, adolfo.garcia-sastre@mssm.edu *and* lozano@avimex.com.mx

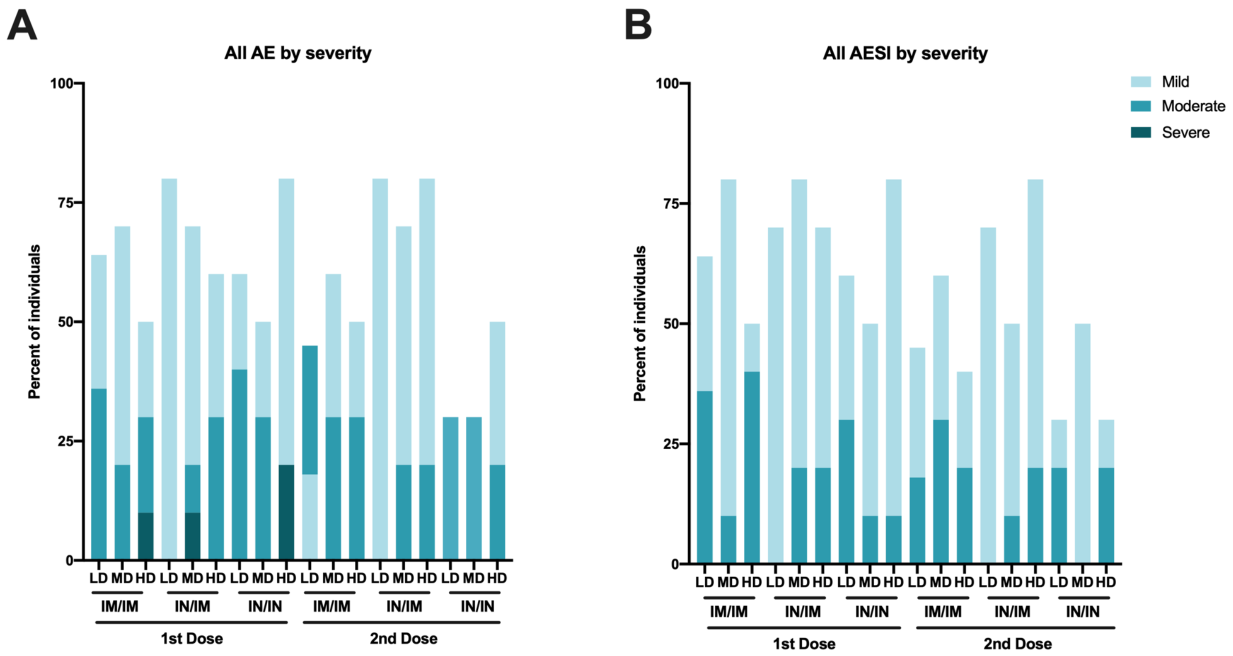

Supplementary Figure 1. **Frequency of all systemic and local adverse events. A** shows the frequency of all registered systemic and local adverse events (AE) up to day 42 after first and second doses for all study groups. **B** shows only solicited adverse events (SoAE). There were less adverse events registered after 2nd dose as compared to 1st dose for all groups. (IM=Intramuscular, IN=Intranasal)

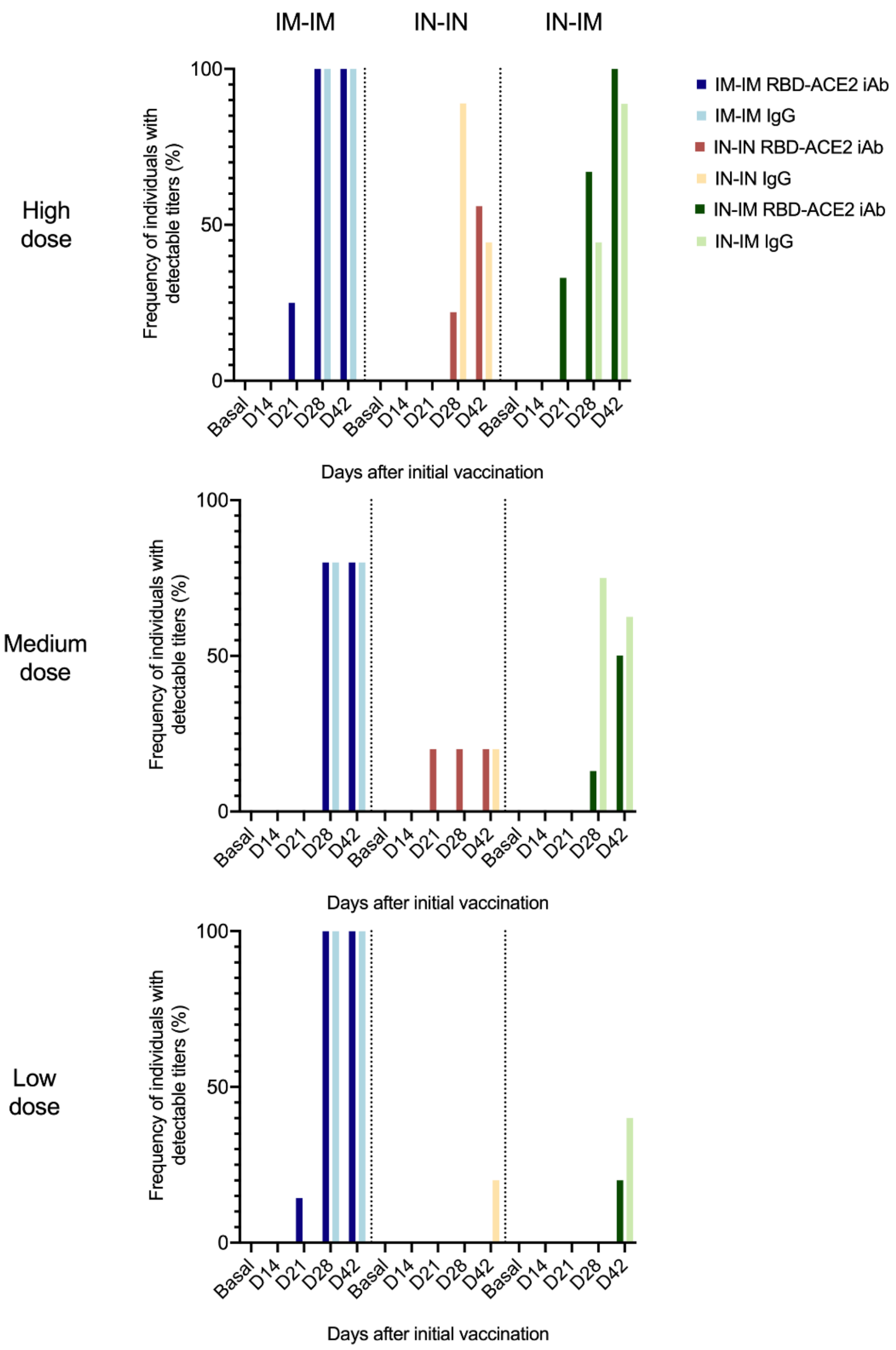

Supplementary Figure 2. Frequency of individuals with detectable spike-reactive and RBD-inhibiting antibody titers. Antibodies against the S1 subunit of the spike protein (which contains the receptor binding domain (RBD)) and antibodies binding to the RBD that inhibited its interaction with the angiotensin-converting enzyme 2 (ACE2) were assessed in vaccinees' sera at baseline and 14, 21, 28, and 42 days after the first vaccine dose administration. Individuals receiving the IM-IM regimen (left column), IN-IN regimen (middle column), or IN-IM (right column), with a high dose (top), medium dose (middle), or low dose (bottom) of the vaccine are shown. S1-IgG = antibodies binding to the S1 spike subunit; iAb = antibodies inhibiting RBD-ACE2 interactions.

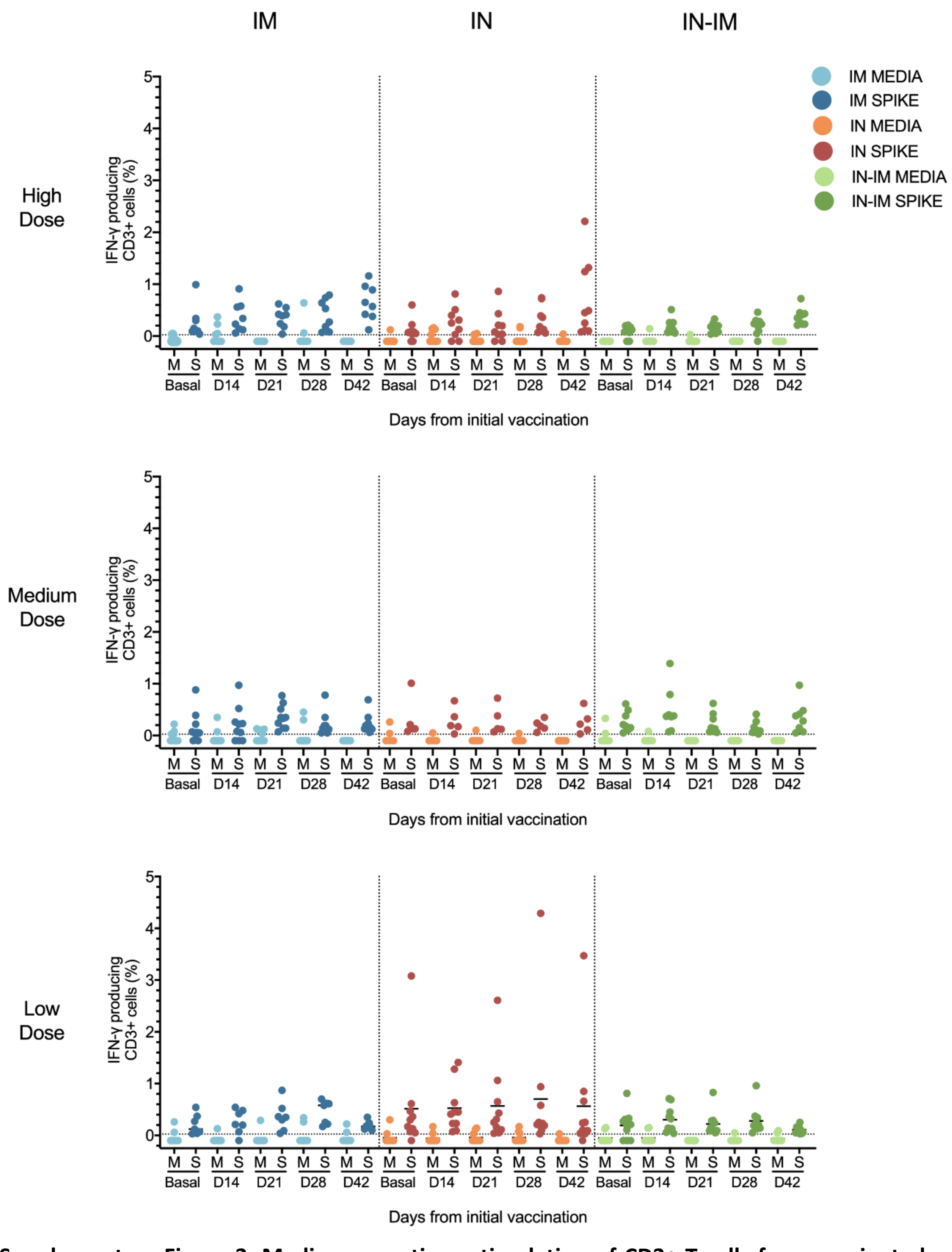

Supplementary Figure 3. Medium vs antigen stimulation of CD3+ T cells from vaccinated volunteers. PBMCs were collected from vaccinees at baseline, and 14, 21, 28 and 42 days after the first vaccine dose administration. Individuals receiving the IM-IM regimen (left column), IN-IN regimen (middle column), or IN-IM (right column) stratified by the vaccine dose received (high, medium, or low) are shown. Activated CD3+ T cells were determined by flow cytometry after 18 h incubation with the recombinant spike protein or with medium only. Frequencies of T cells producing interferon gamma (IFN-γ) are presented.

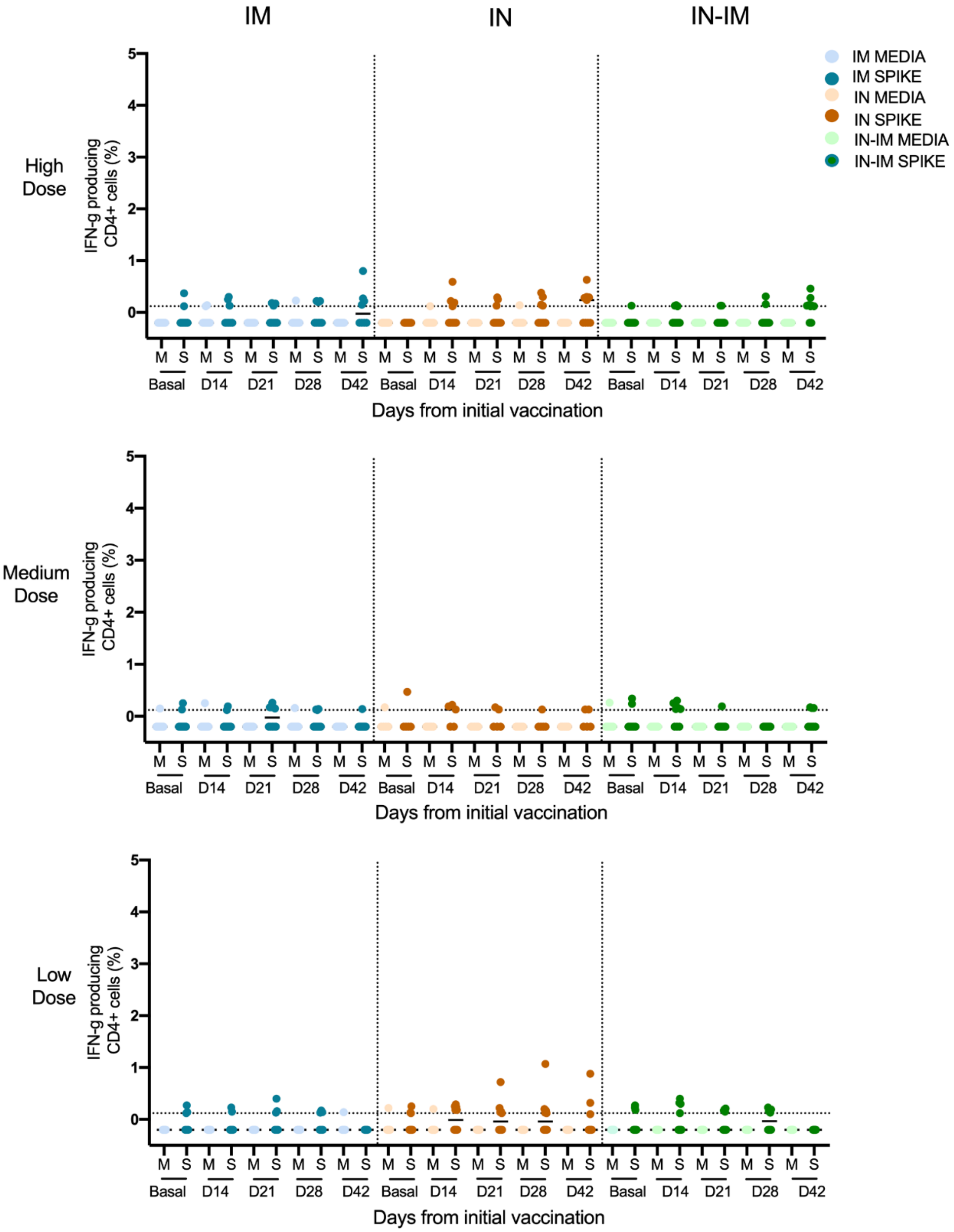

Supplementary Figure 4. Medium vs antigen stimulation of CD3+CD4+ T cells from vaccinated volunteers. PBMCs were collected from vaccinees at baseline, and 14, 21, 28 and 42 days after the first vaccine dose administration. Individuals receiving the IM-IM regimen (left column), IN-IN regimen (middle column), or IN-IM (right column) stratified by the vaccine dose received (high, medium, or low) are shown. Activated CD3+CD4+ T cells were determined by flow cytometry after 18 h incubation with the recombinant spike protein or with medium only. Frequencies of T cells producing interferon gamma (IFN-γ) are presented.

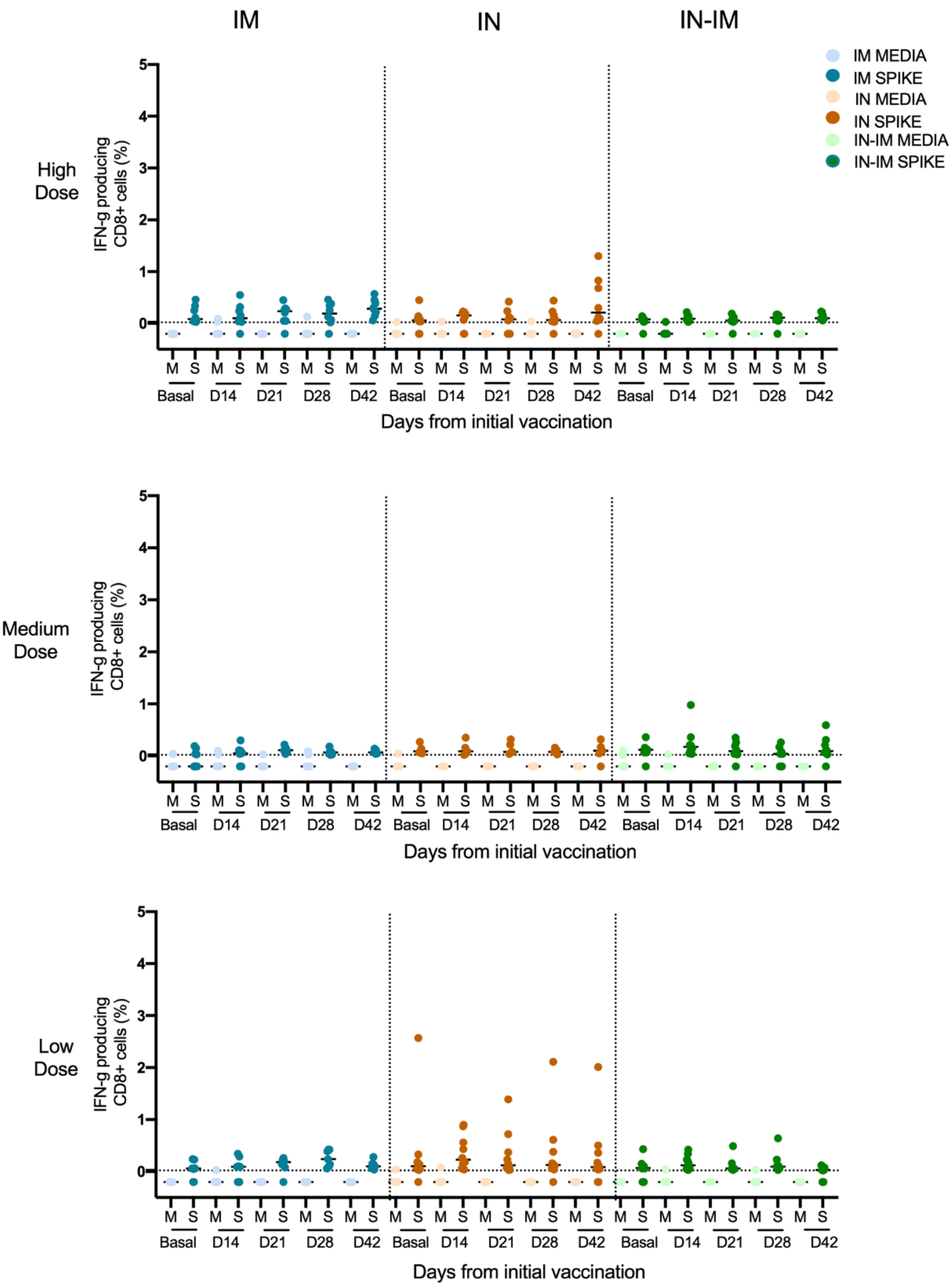

Supplementary Figure 5. Medium vs antigen stimulation of CD3+CD8+ T cells from vaccinated volunteers. PBMCs were collected from vaccinees at baseline, and 14, 21, 28 and 42 days after the first vaccine dose administration. Individuals receiving the IM-IM regimen (left column), IN-IN regimen (middle column), or IN-IM (right column) stratified by the vaccine dose received (high, medium, or low) are shown. Activated CD3+CD8+ T cells were determined by flow cytometry after 18 h incubation with the recombinant spike protein or with medium only. Frequencies of T cells producing interferon gamma (IFN-γ) are presented.

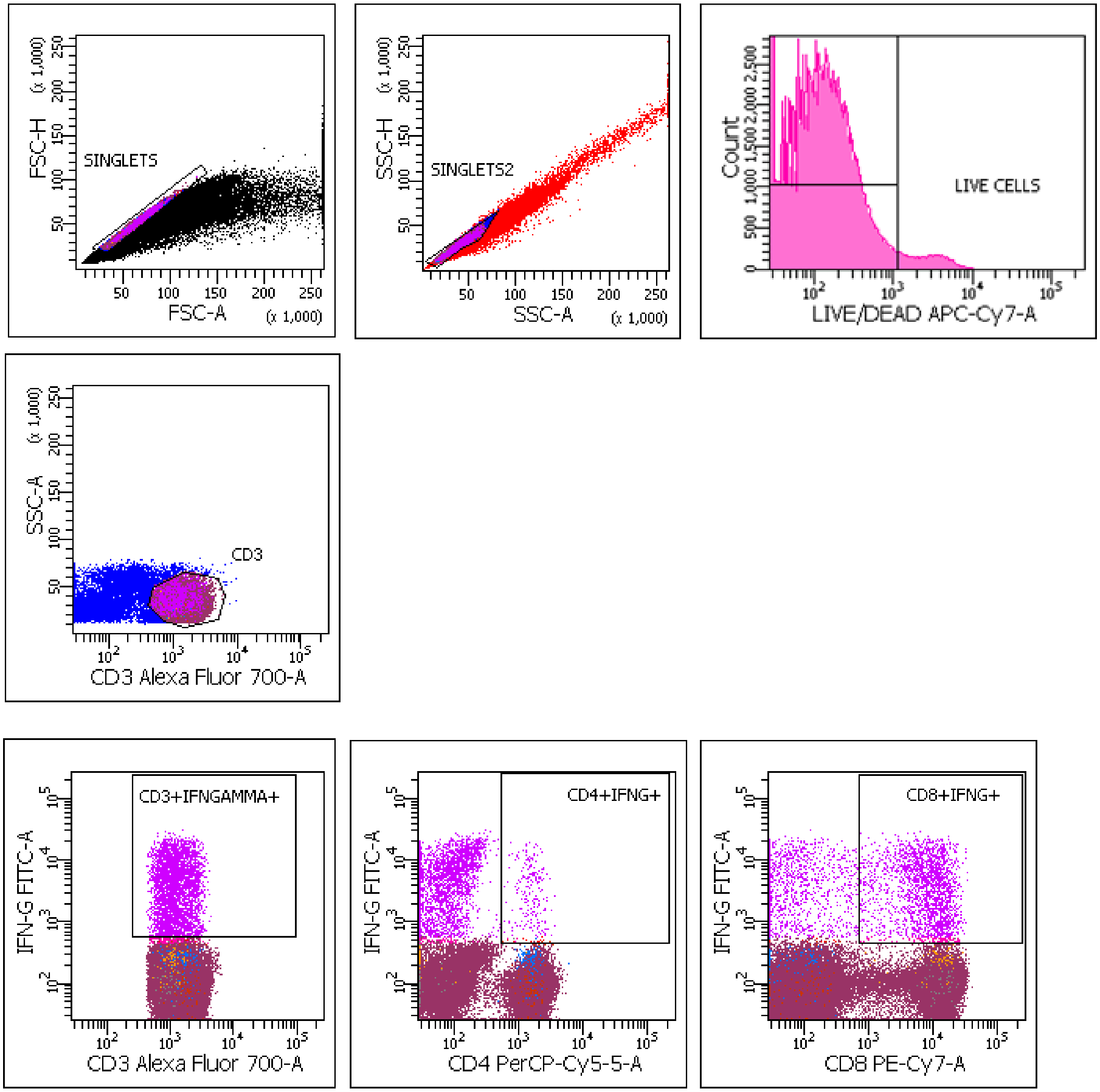

Supplementary Figure 6. Gating strategy for T-cell analysis as shown in Figure 7 and Supplementary Figures 3-5.

Supplementary Table 1. Flow Cytometry antibodies.

Antibody	Fluorochrome	Clone	Dilution	Brand	Catalog Number
Anti-human IFN-⍰	FITC	4S.B3	1:200	BD	554551
Anti-human CD3	Alexa Fluor© 700	SK7	1:33	BioLegend	344822
Anti-human CD4	PerCP/Cy5.5	RPA-T4	1:100	BioLegend	300530
Anti-human CD8	PE/Cy7	53-6.7	1:100	BioLegend	980910

Supplementary Table 2. Flow Cytometry reagents.

Reagent	Brand	Catalog Number
Live/Dead Fixable near 633 or 635nm (work dilution 1:1000)	Invitrogen	L34976A
Anti-CD28/CD49d	BD	347690
Phytohemagglutinin (PHA)	Sigma	
Recombinant Spike Protein, Subunit 1	Raybiotech	230-011101-500
RBC Lysis Buffer	Biolegend	420302
Golgi Stop	BD	554715
Cytofix/Cytoperm	BD	51-2090KZ
Perm/Wash	BD	51-2091KZ
PBS	Lonza	17-516Q
RPMI 1640	Lonza	12-16Q

Supplementary Table 3. Failed enrollment summary.

<table>
<tr><td rowspan="6">Failed Screening</td><td rowspan="6">47</td><td rowspan="2">Previous or ongoing SARS-CoV2 infection</td><td rowspan="2">40 (85%)</td><td>Positive to Anti S antibodies</td><td>39</td></tr>
<tr><td>SARS-CoV-2 positive PCR</td><td>1</td></tr>
<tr><td rowspan="4">Other</td><td rowspan="4">7 (15%)</td><td>Abnormal ECG</td><td>4</td></tr>
<tr><td>Pregnancy</td><td>1</td></tr>
<tr><td>Positive HIV</td><td>1</td></tr>
<tr><td>Diabetes</td><td>1</td></tr>
<tr><td>Retired Consent</td><td colspan="5">2</td></tr>
<tr><td>COVID-19 direct exposure</td><td colspan="5">2</td></tr>
<tr><td>Total</td><td colspan="5">51</td></tr>
</table>
