# Supplementary material for: Interim safety and immunogenicity results from an NDV-based COVID-19 vaccine phase I trial in Mexico
Source: NPJ Vaccines. 2023 May 10;8:67. doi: 10.1038/s41541-023-00662-6 (PMC10170424; doi:10.1038/s41541-023-00662-6)
Supplement: Supplementary file 2 — REPORTING SUMMARY [file 41541_2023_662_MOESM2_ESM.pdf]

nature portfolio

Corresponding author(s): Florian Krammer

Last updated by author(s): Mar 20, 2023

Reporting Summary

Nature Portfolio wishes to improve the reproducibility of the work that we publish. This form provides structure for consistency and transparency in reporting. For further information on Nature Portfolio policies, see our Editorial Policies and the Editorial Policy Checklist.

Statistics

For all statistical analyses, confirm that the following items are present in the figure legend, table legend, main text, or Methods section.

n/a	Confirmed	
☐	☒	The exact sample size (*n*) for each experimental group/condition, given as a discrete number and unit of measurement
☐	☒	A statement on whether measurements were taken from distinct samples or whether the same sample was measured repeatedly
☐	☒	The statistical test(s) used AND whether they are one- or two-sided<br>*Only common tests should be described solely by name; describe more complex techniques in the Methods section.*
☒	☐	A description of all covariates tested
☐	☒	A description of any assumptions or corrections, such as tests of normality and adjustment for multiple comparisons
☐	☒	A full description of the statistical parameters including central tendency (e.g. means) or other basic estimates (e.g. regression coefficient) AND variation (e.g. standard deviation) or associated estimates of uncertainty (e.g. confidence intervals)
☐	☒	For null hypothesis testing, the test statistic (e.g. *F*, *t*, *r*) with confidence intervals, effect sizes, degrees of freedom and *P* value noted<br>*Give P values as exact values whenever suitable.*
☒	☐	For Bayesian analysis, information on the choice of priors and Markov chain Monte Carlo settings
☒	☐	For hierarchical and complex designs, identification of the appropriate level for tests and full reporting of outcomes
☐	☒	Estimates of effect sizes (e.g. Cohen's *d*, Pearson's *r*), indicating how they were calculated

Our web collection on statistics for biologists contains articles on many of the points above.

Software and code

Policy information about availability of computer code

Data collection	Safety data were collected in electronic Case Report Forms in the specialized program Datatrack. Experiments data was captured in Excel spreadsheets
Data analysis	Data was analyzed and/or graphed in Prism.

For manuscripts utilizing custom algorithms or software that are central to the research but not yet described in published literature, software must be made available to editors and reviewers. We strongly encourage code deposition in a community repository (e.g. GitHub). See the Nature Portfolio guidelines for submitting code & software for further information.

Data

Policy information about availability of data

All manuscripts must include a data availability statement. This statement should provide the following information, where applicable:

- Accession codes, unique identifiers, or web links for publicly available datasets
- A description of any restrictions on data availability
- For clinical datasets or third party data, please ensure that the statement adheres to our policy

The data that support the findings of this study are available from the corresponding author upon reasonable request.

Human research participants

Policy information about studies involving human research participants and Sex and Gender in Research.

Reporting on sex and gender	Sex and gender was collected as self-reported by the participants. As reported in the paper Fig. 1C 44 males and 47 females participated in the study.
Population characteristics	Clinically healthy female and male participants between 18 and 55 years of age without prior immunity to SARS-CoV-2 were enrolled
Recruitment	This Phase I study was designed as a non-randomized open label study without placebo control group. Ninety volunteers were assigned to one of nine treatment groups in the order of enrolment. No biases were expected.
Ethics oversight	Ethics, Biosafety and Research Committees of the clinical research site Hospital Medica Sur (03-2021-CI/CEI/CB-156)

Note that full information on the approval of the study protocol must also be provided in the manuscript.

Field-specific reporting

Please select the one below that is the best fit for your research. If you are not sure, read the appropriate sections before making your selection.

- [x] Life sciences
- [ ] Behavioural & social sciences
- [ ] Ecological, evolutionary & environmental sciences

For a reference copy of the document with all sections, see nature.com/documents/nr-reporting-summary-flat.pdf

Life sciences study design

All studies must disclose on these points even when the disclosure is negative.

Sample size	90 volunteers
Data exclusions	Data from participants that presented COVID-19 infection were excluded from the immunogenicity analysis
Replication	As a First-in-humans experiments, these cannot be repeated but the validity was determined by registering the adverse events.
Randomization	This was a non-randomized study. Participants were assigned to the groups in the order of enrollment to avoid biases and no other criteria was used for assignment to groups to avoid biases
Blinding	As this was a safety study without placebo control the study was open label.

Reporting for specific materials, systems and methods

We require information from authors about some types of materials, experimental systems and methods used in many studies. Here, indicate whether each material, system or method listed is relevant to your study. If you are not sure if a list item applies to your research, read the appropriate section before selecting a response.

Materials & experimental systems

n/a	Involved in the study	
[ ]	[x]	Antibodies
[x]	[ ]	Eukaryotic cell lines
[x]	[ ]	Palaeontology and archaeology
[x]	[ ]	Animals and other organisms
[ ]	[x]	Clinical data
[ ]	[ ]	Dual use research of concern

Methods

n/a	Involved in the study	
[x]	[ ]	ChIP-seq
[ ]	[x]	Flow cytometry
[x]	[ ]	MRI-based neuroimaging

Antibodies

Antibodies used	Anti-human IFN-[illegible] FITC 4S.B3 1:200 BD 554551<br>Anti-human CD3 Alexa Fluor[illegible] 700 SK7 1:33 BioLegend 344822<br>Anti-human CD4 PerCP/Cy5.5 RPA-T4 1:100 BioLegend 300530

	Anti-human CD8 PE/Cy7 53-6.7 1:100 BioLegend 980910
Validation	We rely on the data provided by the commercial manufacturers of these antibodies for validation (BD, BioLegend).

Clinical data

Policy information about clinical studies

All manuscripts should comply with the ICMJE guidelines for publication of clinical research and a completed CONSORT checklist must be included with all submissions.

Clinical trial registration	NCT04871737
Study protocol	https://clinicaltrials.gov/ct2/show/NCT04871737
Data collection	Data was collected at one research site: Hospital Medica Sur
Outcomes	Primary outcomes of the study were established as follows:<br>● To evaluate the safety of three concentrations (107.0-7.49, 107.5-7.99, 108.0-8.49 EID50%/dose) of the recombinant vaccine against SARS-CoV-2 based on a Newcastle Disease virus (rNDV) administered twice intramuscularly, twice intranasally or intranasally followed by intramuscularly in healthy volunteers<br>Secondary outcomes<br>● To evaluate the immunogenicity of three concentrations (107.0-7.49, 107.5-7.99, 108.0-8.49 EID50%/dose) of the recombinant vaccine against SARS-CoV-2 based on a Newcastle Disease virus (rNDV) administered twice intramuscularly, twice intranasally or intranasally followed by intramuscularly in healthy volunteers<br>● To evaluate the nasal mucosal humoral immunity of three concentrations (107.0-7.49, 107.5-7.99, 108.0-8.49 EID50%/dose) of the recombinant vaccine against SARS-CoV-2 based on a Newcastle Disease virus (rNDV)

Dual use research of concern

Policy information about dual use research of concern

Hazards

Could the accidental, deliberate or reckless misuse of agents or technologies generated in the work, or the application of information presented in the manuscript, pose a threat to:

No	Yes	
☒	☐	Public health
☒	☐	National security
☒	☐	Crops and/or livestock
☒	☐	Ecosystems
☒	☐	Any other significant area

Experiments of concern

Does the work involve any of these experiments of concern:

No	Yes	
☒	☐	Demonstrate how to render a vaccine ineffective
☒	☐	Confer resistance to therapeutically useful antibiotics or antiviral agents
☒	☐	Enhance the virulence of a pathogen or render a nonpathogen virulent
☒	☐	Increase transmissibility of a pathogen
☒	☐	Alter the host range of a pathogen
☒	☐	Enable evasion of diagnostic/detection modalities
☒	☐	Enable the weaponization of a biological agent or toxin
☒	☐	Any other potentially harmful combination of experiments and agents

Flow Cytometry

Plots

Confirm that:

☒ The axis labels state the marker and fluorochrome used (e.g. CD4-FITC).

☒ The axis scales are clearly visible. Include numbers along axes only for bottom left plot of group (a 'group' is an analysis of identical markers).

☒ All plots are contour plots with outliers or pseudocolor plots.

☒ A numerical value for number of cells or percentage (with statistics) is provided.

Methodology

Sample preparation	Whole blood diluted 1:1 was stimulated with RPMI 1640 medium (Lonza, Walkersville, MD) 0.99 g/mL of S1 subunit of spike protein in the presence of anti-CD28/CD49d (BD, San Jose CA) for 18 h20 min at 37°C in 5% CO2. GolgiStop (BD, San José, CA) was added, and the samples were cultured additionally for 4 h. Medium was used as a negative control and 10 g/mL of PHA (Sigma-Aldrich) as a positive control. Samples were washed with phosphate buffered saline (PBS) without Ca2+ and Mg2+ (Lonza), and stained with Live/Dead near-IR Dead Cell Stain Kit for 633 or 635nm excitation (Invitrogen, Eugene, OR), for 15 min at room temperature in the dark. Then red blood cells (RBCs) were lysed with RBC lysis buffer (BD) for 10 min followed by a washing step with staining buffer PBS without Ca2+ and Mg2+ supplemented with 1% fetal bovine serum (FBS) and 0.1% NaN3). Cell surface staining was performed using a cocktail of anti-human CD3, CD4 and CD8 antibodies in staining buffer for 15 min at room temperature in the dark. After an additional washing step with staining buffer, the cells were fixed and further permeabilized using BD Cytofix/Cytoperm following the manufacturer's instructions. Intracellular staining was performed in Cytoperm using a anti-human IFN-γ antibodies for 30 min at room temperature in the dark. Cells were washed with BD Perm/Wash buffer and further resuspended in PBS. Cells were kept at 4°C in the dark until acquisition and analysis. Unstained and fluorescence minus one (FMO) controls were included. Details of the antibodies used in the flow cytometry assay are listed in Supplementary Table 1, and the rest of the reagents in Supplementary Table 2. At least 200,000 events of the lymphocyte region in a forward scatter (FSC) vs side scatter (SSC) scatter plot were acquired in a fluorescence-activated cell sorter (FACS) Aria II (BD). Analysis was performed using FACS Diva 8.0. The gates applied for the identification of SARS-CoV-2 antigen-specific cytokine-producing CD3+, CD4+ or CD8+ cells were defined using the FMO controls and used for limit of detection (LOD) (37).
Instrument	Cell sorter (FACS) Aria II (BD)
Software	FACS Diva 8.0
Cell population abundance	*Describe the abundance of the relevant cell populations within post-sort fractions, providing details on the purity of the samples and how it was determined.*
Gating strategy	*Describe the gating strategy used for all relevant experiments, specifying the preliminary FSC/SSC gates of the starting cell population, indicating where boundaries between "positive" and "negative" staining cell populations are defined.*

☒ Tick this box to confirm that a figure exemplifying the gating strategy is provided in the Supplementary Information.
