# Supplementary material for: Interim safety and immunogenicity results from an NDV-based COVID-19 vaccine phase I trial in Mexico
Source: NPJ Vaccines. 2023 May 10;8:67. doi: 10.1038/s41541-023-00662-6 (PMC10170424; doi:10.1038/s41541-023-00662-6)
Supplement: Supplementary file 3 — Supplementary Appendix 1 [file 41541_2023_662_MOESM3_ESM.pdf]

# Protocol Title

Dose-escalation, open-label, non-randomized Phase I study to evaluate safety and immunogenicity of three concentrations ($10^{7.0}$, $10^{7.5}$, $10^{8.0}$ $EID_{50}$/dose) of a recombinant vaccine against SARS-CoV-2 based on an active viral vector of the Newcastle Disease Virus (rNDV), administered by intranasal and intramuscular route to healthy volunteers.

## Protocol ID

AVIMEX-SARS-CoV-2-VAC-rNDV

**Protocol No.:** AVX-SARS-CoV-2-VAC-001

| | |
|---|---|
| **Investigation product** | Recombinant vaccine against SARS-CoV-2 based on a viral vector of the Newcastle Disease Virus (rNDV). |
| **Protocol version** | Version 1 |
| **Version date** | 01-Mar-2021 |
| **Sponsor** | Laboratorio Avi-Mex S.A. de C.V. (Avimex*) Maíz 18 Colonia Granjas Esmeralda, Ciudad de México, C.P. 09810.<br>Phone: (55) 54450460/Fax: 54450462/Hotline: (55) 54450465<br>E-mail: lozano@avimex.com.mx<br>www.avimex.com.mx |
| **Investigation site** | |
| **Address** | Centro Integral de Diagnóstico y Tratamiento<br>Hospital Médica Sur, S.A.B. de C.V.<br>Puente de Piedra 150, Toriello Guerra, Tlalpan, 14050,<br>Ciudad de México, México. |
| **Sponsor** | Laboratorio Avi-Mex S.A. de C.V. (Avimex*)<br>Consejo Nacional de Ciencia y Tecnología<br>AMEXCID |

**Confidentiality Notice**

The information contained herein is confidential and property of Laboratorio Avi-Mex S.A. de C.V. This document should not be disclosed to anyone other than the study personnel or members of the Research Ethics Committee and the Research Committee. The information herein may not be used for any other purpose other than evaluation or carrying out of clinical research without prior written authorization by Laboratorio Avi-Mex S.A. de C.V. Should you have any questions regarding use or distribution of this document, please do not hesitate to call us: 5554450465.

# PROTOCOL SINOPSIS

| | |
|---|---|
| **TITLE** | Dose-escalation, open-label, non-randomized Phase I study to evaluate safety and immunogenicity of three concentrations ($10^{7.0}$, $10^{7.5}$, $10^{8.0}$ $EID_{50}$/dose) of a recombinant vaccine against SARS-CoV-2 based on an active viral vector of the Newcastle Disease Virus (rNDV), administered by intranasal and intramuscular route to healthy volunteers. |
| **PROTOCOL VERSION** | Version 1. |
| **SPONSOR** | Laboratorio Avi-Mex S.A. de C.V. (Avimex*). |
| **PROJECT** | Clinical Trial. |
| **INDICATION** | Target population, healthy subjects. |
| **OBJECTIVE** | **Primary Objectives:**<br>• To evaluate safety of three concentrations ($10^{7.0-7.49}$, $10^{7.5-7.99}$, $10^{8.0-8.49}$ $EID_{50}$/dose) of the recombinant vaccine against SARS-CoV-2 based on a viral vector of the Newcastle Disease Virus (rNDV), administered two times by intramuscular route in healthy volunteers.<br>• To evaluate safety of three concentrations ($10^{7.0-7.49}$, $10^{7.5-7.99}$, $10^{8.0-8.49}$ $EID_{50}$/dose) of the recombinant vaccine against SARS-CoV-2 based on a viral vector of the Newcastle Disease Virus (rNDV), administered two times by intranasal route in healthy volunteers.<br>• To evaluate safety of three concentrations ($10^{7.0-7.49}$, $10^{7.5-7.99}$, $10^{8.0-8.49}$ $EID_{50}$/dose) of the recombinant vaccine against SARS-CoV-2 based on a viral vector of the Newcastle Disease Virus (rNDV), administered two times, the first intranasal and the second by intramuscular route in healthy volunteers. |

**Secondary Objectives:**

- To evaluate immunogenicity of three concentrations ($10^{7.0\text{-}7.49}$, $10^{7.5\text{-}7.99}$, $10^{8.0\text{-}8.49}$ $EID_{50}$/dose) of the recombinant vaccine against SARS-CoV-2 based on a viral vector of the Newcastle Disease Virus (rNDV), administered two times by intramuscular route in healthy volunteers.

  Immunogenicity shall be determined by:
  Titers of circulating IgG and IgM antibodies.
  Titers of neutralizing antibodies.
  T-cell immunity response.

- To evaluate immunogenicity of three concentrations ($10^{7.0\text{-}7.49}$, $10^{7.5\text{-}7.99}$, $10^{8.0\text{-}8.49}$ $EID_{50}$/dose) of the recombinant vaccine against SARS-CoV-2 based on a viral vector of the Newcastle Disease Virus (rNDV), administered two times by intranasal route in healthy volunteers.

  Immunogenicity shall be determined by:
  Titers of circulating IgG and IgM antibodies.
  Titers of neutralizing antibodies.
  T-cell immunity response.

- To evaluate immunogenicity of three concentrations ($10^{7.0\text{-}7.49}$, $10^{7.5\text{-}7.99}$, $10^{8.0\text{-}8.49}$ $EID_{50}$/dose) of the recombinant vaccine against SARS-CoV-2 based on a viral vector of the Newcastle Disease Virus (rNDV), administered two times each: first by intranasal route; and second by intramuscular route in healthy volunteers.

  Immunogenicity shall be determined by:
  Titers of circulating IgG and IgM antibodies.
  Titers of neutralizing antibodies.
  T-cell immunity response.

- To evaluate nasal mucosal humoral immunity of three concentrations ($10^{7.0\text{-}7.49}$, $10^{7.5\text{-}7.99}$, $10^{8.0\text{-}8.49}$ $EID_{50}$/dose) of the recombinant vaccine against SARS-CoV-2 based on a viral vector of the Newcastle Disease Virus (rNDV).

Mucosal humoral immunity shall be determined by:

Titers of IgA antibodies.
Titers of neutralizing IgA antibodies.

**HYPOTHESIS**

- The recombinant vaccine against SARS-CoV-2 based on a viral vector of the Newcastle Disease Virus (rNDV), administered two times by intramuscular route is safe and induces humoral and cellular immune response against SARS-CoV-2 virus.
- The recombinant vaccine against SARS-CoV-2 based on a viral vector of the Newcastle Disease Virus (rNDV), administered two times by intranasal route is safe and induces humoral and cellular immune response against SARS-CoV-2 virus.
- The recombinant vaccine against SARS-CoV-2 based on a viral vector of the Newcastle Disease Virus (rNDV), administered two times each: first by intranasal route; and second by intramuscular route is safe and induces humoral and cellular immune response against SARS-CoV-2 virus.
- The recombinant vaccine against SARS-CoV-2 based on a viral vector of the Newcastle Disease Virus (rNDV), administered by intranasal route induces humoral and mucosal immune response against SARS-CoV-2 virus.

**STUDY DESIGN**

Dose-escalation, open-label, non-randomized Phase I clinical trial which uses two administration routes to evaluate a vaccine safety and immunogenicity.

**NUMBER OF SUBECTS** 90 healthy volunteers.
**NUMBER OF CENTRES** A single research site in Mexico City.

**TARGET POPULATION** Healthy volunteers.

**STUDY PERIOD** 12 months.

**SELECTION CRITERIA**

**Inclusion Criteria**

- Adult men and women ≥18 years old and ≤55 years old.
- Signed informed consent.
- No respiratory disease within last 21 days prior to the first dose administration.
- Body Mass Index from 18.0 to 29.0 kg/$m^2$.
- Negative RT-PCR for SARS-CoV-2 infection.
- Negative test for SARS-CoV-2 IgM and IgG antibodies.
- $O_2$ saturation ≥92% by pulse oximetry.
- Normal CT scan of thorax.
- No symptoms from clinical history and normal physical exam at the screening visit.
- Lab test values within normal ranges according to the local laboratory for all the following tests:
  - Urinalysis.
  - Liver enzymes.
  - Renal function tests.
  - Cholesterol and triglycerides.
  - Fasting glucose.
  - Haematology.
- Negative tests for HBsAg, anti-HCV and anti-HIV antibodies. Negative VDRL test.
- Normal electrocardiogram.
- Negative pregnancy test for women of childbearing potential.
- Agreement of all sexually active volunteers to use highly effective contraceptives over the study period and up to 30 days after the last administration of the vaccine dose.
- Commitment from all the participants to keep social distancing, use facemask (when social distancing is not possible) and frequent handwashing with soap or antibacterial gel during the study period.

**Exclusion Criteria**

- History of hypersensitivity or allergy to any vaccine ingredient of the vaccine.
- History of severe anaphylactic reaction.
- History of seizures.
- History of chronic diseases or cancer.
- Vaccination against SARS-CoV-2 with either approved or experimental vaccines.
- Participation in any other study with an experimental intervention within the last 3 months.
- Administration of any other drug or herbal preparation, as well as "alternative medicine" treatments, transfer factor, chlorine dioxide or others within the last 30 days prior screening, which the investigator considers they could interfere with subject safety or data analysis.
- Any other vaccine administered within the last 30 days, including influenza vaccine.
- Fever at the time of enrolment.
- Blood transfusion or blood components transfusion within the last 4 months.
- Regular activity related to work, social interaction or entertainment that represents an exposure to SARS-CoV-2 higher than that of the general population, as per investigator judgement.
- Poultry farm work, including contact with fighting cocks.
- Alcohol and drugs abuse, which may interfere with the development of the research protocol.
- Any medical or not medical condition that could threaten patient safety or interfere with vaccine evaluation or analysis of the study results, as per investigator judgement.

**STUDY VACCINE**

Recombinant vaccine against SARS-CoV-2 based on a viral vector of the Newcastle Disease Virus (rNDV) *LaSota* strain of low virulence expressing the SARS-CoV-2 S-glycoprotein.

**DOSE/ ADMINISTRATION ROUTE**

Concentrations of the Study Vaccine at Embryo Infectious Dose (EID):

- Low dose – $10^{7.0\text{-}7.49}$ $EID_{50}$/dose.
- Medium dose – $10^{7.5\text{-}7.99}$ $EID_{50}$/dose.
- High dose – $10^{8.0\text{-}8.49}$ $EID_{50}$/dose.

Vaccine is administered in two vaccination events: Day 0 and Day 21.

There are two administration routes:

- Intramuscular: deltoid muscle.
- Intranasal: through nasal spray applicator into both nostrils.

Administration schemes of both, the first and second dose, respectively:

- Intramuscular – intramuscular (IM-IM).
- Intranasal – intranasal (IN-IN).
- Intranasal – intramuscular (IN-IM).

**EFFICACY ENDPOINTS**

- Titers of circulating anti-SARS-CoV-2 antibodies. Seroconversion defined as the appearance of specific circulating antibodies (IgG and IgM) titers for SARS-CoV-2 S-protein epitopes determined by the immunoassay ELISA.
- Titers of neutralizing antibodies.
- T-cell immunity response, which includes assessment of pro-inflammatory cytokine release after spike protein challenge.
- Titers of IgA in nasal mucosa.

**SAFETY ENDPOINTS**

**Adverse events.**

**Lab test values.**

**Haematology** – Hemoglobin, erythrocytes, hematocrit, platelets, leukocytes, including differential count (neutrophils, lymphocytes, monocytes, eosinophils, and basophils).
**Blood chemistry** – Glucose, liver function tests (AST, ALT, alkaline phosphatase, LDH), kidney function tests (serum creatinine, blood urea nitrogen), cholesterol, triglycerides.

**PROCEDURE OF ESCALATION DOSE AND ADMINISTRATION ROUTE**

90 volunteers shall be assigned, following the order they enter the study, into nine treatment groups, determined by three different concentrations of the vaccine and three different administration schemes, as shown in the following table:

| Administration route / Concentration | First and second administration by intramuscular route | First and second administration by intranasal route | First administration by intranasal route and second by intramuscular route |
|---|---|---|---|
| $10^{7.0-7.49}$ $EID_{50}$/dose | 10 volunteers | 10 volunteers | 10 volunteers |
| $10^{7.5-7.99}$ $EID_{50}$/dose | 10 volunteers | 10 volunteers | 10 volunteers |
| $10^{8.0-8.49}$ $EID_{50}$/dose | 10 volunteers | 10 volunteers | 10 volunteers |

EID: embryo infectious dose.

First vaccination of each treatment group shall be done sequentially to sentinel subjects according to the dose and administration route of the following table:

| | Day 1 | Day 2 | Day 3 | Day 4 | Day 5 | Day 6 | Day 7 | Day 8 | Day 9 |
|---|---|---|---|---|---|---|---|---|---|
| Baseline dose concentration | **Low ($10^{7.0-7.49}$ $EID_{50}$/dose)** | | | **Medium ($10^{7.5-7.99}$ $EID_{50}$/dose)** | | | **High ($10^{8.0-8.49}$ $EID_{50}$/dose)** | | |
| **IM route** | Subject 1 | Subject 3 | Subject 5 | Subject 7 | Subject 9 | Subject 11 | Subject 13 | Subject 15 | Subject 17 |
| **IN route** | Subject 2 | Subject 4 | Subject 6 | Subject 8 | Subject 10 | Subject 12 | Subject 14 | Subject 16 | Subject 18 |
| | **Day 10 to 15** | | | | | | | | |
| | **Data Safety Monitoring Board Evaluation** | | | | | | | | |

Once all sentinel groups have been vaccinated and the Data Safety Monitoring Board (DSMB) considers safety conditions have been fulfilled, the rest of the volunteers shall be vaccinated with dose-escalation according to the following table:

| Baseline dose concentration | **Day 16**<br>**Low** | **Day 17**<br>**Low** | **Day 18**<br>**Low** |
|---|---|---|---|
| IM route | S19, S21, S23 | S28, S30, S32 | S37, S38 |
| IN route | S20, S22, S24<br>S25, S26, S27 | S29, S31, S33<br>S34, S35, S36 | S39, S40,<br>S41, S42 |
| Baseline dose concentration l | **Day 19**<br>**Medium** | **Day 20**<br>**Medium** | **Day 21**<br>**Medium** |
| IM route | S43, S45, S47 | S52, S54, S56 | S61<br>S62 |
| IN route | S44, S46, S48<br>S49, S50,51 | S53, S55, S57<br>S58, S59, S60 | S63, S64,<br>S65, S66 |
| Baseline dose concentration | **Day 22**<br>**High** | **Day 23**<br>**High** | **Day 24**<br>**High** |
| IM route | S67, S69, S71 | S76, S78, S80 | S85<br>S86 |
| IN route | S68, S70, S72<br>S73, S74, S75 | S77, S79, S81<br>S82, S83, S84 | S87, S88,<br>S89, S90 |

IM= intramuscular; IN= intranasal; S= subject.

Second event of vaccination shall be on Day 21 with prior authorization of the Data Safety Monitoring Board.

**STATISTICS**

**Sample Size Calculation**

No sample size calculation is carried out. It is estimated to include 10 healthy volunteers per dose group and administration route in order to reach a total sample of 90 healthy volunteers.

**STATISTICAL ANALYSIS PLAN**

**Baseline and Demographic Characteristics**

To evaluate vaccine groups and the administration route, each independent group with the same baseline characteristics shall be tested. Student-*t* test and ANOVA shall be applied to equality of means in continuous variables and non-parametric tests to equality of medians in count variables.

**Characteristics of outcome and safety variables**

**Immunogenicity**

Titers of SARS-CoV-2 anti-spike protein antibodies (IgG, IgM and IgA) shall be given and analysed for the subjects and per treatment group, according to the following:

SARS-CoV-2 anti-spike protein antibodies shall be expressed as Geometric Mean Titer (GMT) with a 95% Confidence Interval (CI).

Geometric Mean Titer and its two-sided 95% Confidence Interval (CI) shall be calculated the following days: 0 (Baseline), 14 and 21, 28, 35, 42, 90, 180 and 365.

ANOVA shall be used to analyse log-transformed antibody titers and the Wilcoxon rank-sum test for non-normal distribution data.

When there is a significant overall difference among the three concentration groups paired comparisons shall be performed and the differences shall be estimated with 95% CI.

Two-sided 95% CI for GMT shall be calculated by back-transformation of the 95% confidence limits based on a Student-*t* test for titrations with $\log_{10}$-transformation.

Ratio of subjects with a titration rate above the parameter established for IgG, IgM and IgA (95% CI) shall be measured the following days: 14, 21, 28, 35, 42, 90, 180 and 365.

The percentage of subjects showing IgG, IgM and IgA antibodies and their corresponding two-sided 95% CI shall be reported.
For the presence of each antibody (IgG, IgM and IgA) the CI shall be found by using the F distribution method given in Collett (Collett, 1991).

Seroconversion rate and 95% CI shall be determined on Day 14 or 21 regarding Day 0 of vaccination. Seroconversion is defined as the appearance of specific circulating antibodies (IgG, IgM and IgA) titers for SARS-CoV-2 S-protein epitopes determined by the immunoassay ELISA.

Total percentage of subjects showing seroconversion and their corresponding two-sided 95% CI shall be reported. Analyses for both, total of subjects and subgroup of subjects resulting seronegative and seropositive shall be performed.

Geometric Mean Ratio (GMR) and its 95% CI for ratios of Day 14 or 21 are divided by the antibodies (IgG and IgM) appearance on Day 1. The GMR and its two-sided 95% CI shall be shown. GMR CI shall be calculated by back-transformation of the 95% confidence limits estimated by using the Student-*t* test for differences (between Day 14 or 21 and Day 1) of antibodies (IgG and IgM) appearance with $\log_{10}$-transformation.

Geometric means confidence intervals were calculated by using the Student-*t* distribution in log-transformed data. Seroconversion estimated by ELISA shall be defined as a 4-fold (or higher) increase in the antibodies titre over the baseline value (from results in preclinical studies).

**Neutralization Capability of Circulating Antibodies**

The ANOVA test shall be used to analyse the circulating antibodies neutralization capability by estimating 95% CI.

**T-Cells Immunity Response**

Cell response shall be shown as ratio of positive responders.

Fisher's exact test or $\chi^2$ test shall be used to analyse categorical values.

**Analysis of Comparison between Antibodies Titers and the Administration Route (IN-IN, IM-IM, IN-IM)**

A two-way ANOVA shall be used to compare differences between the administration route and concentration vs antibody-mediated immune response induced.

**Safety Data Analysis**

**Adverse Events**

Percentages of adverse events occurred during the study shall be calculated. Adverse events should be documented with standardised terminology (MedDRA). For purposes of this study adverse events shall be considered under the following terms:

- Adverse Events of Special Interest (AESIs), as defined in this protocol.
- Adverse Events (AEs), as defined by the GCP – ICH/E6R2.
- Serious Adverse Events (SAEs), as defined by the GCP – ICH/E6R2.

Adverse events shall be assessed as follows:

To analyse adverse events of special interest (AESIs), number and percentage of subjects showing them after each vaccine administration shall be assessed.

To analyse adverse events (AEs), number and percentage of subjects showing them after each vaccine administration shall be assessed.

To analyse serious adverse events (SAEs), number and percentage of subjects showing them after each vaccine administration shall be assessed.

Description of adverse events should be as follows:

Adverse events of special interest shall be analysed within 7 days following administration of each vaccine.

Adverse events shall be analysed within 21 days following administration of the vaccine.

AESI, AE and SAE shall be identified according to the MedDRA terminology, i.e., severity (mild, moderated, or severe), start and end date, vaccine correlation and medical management.

AESIs shall be considered related with the vaccination.

Incidence of each adverse event shall be compared among groups by using the two-proportion z-test.

**Results of Clinical Laboratory and Physical Exam**

Results of clinically significant variations in the physical, clinical and laboratory examinations shall be established according to the vaccine concentration route assigned to the subject and then compared with each other.

Statistical analysis of adverse events, clinical laboratory variables and physical examination results shall be performed by comparing their pre- and post- values related to normal reference values.

A multivariate analysis shall be carried out to establish possible effects on immunogenicity, as well as the safety profile of candidate concentrations for the vaccine.

A two-tailed hypothesis test shall be performed with an alpha ($\alpha$) value of 0.05.

Safety endpoints shall be expressed as frequencies (%) with exact binomial 95% CIs; while immunological endpoints shall be expressed as medians and interquartile ranges (IQR), and these analyses shall be only for descriptive purposes.

**Intermediate and Final Analyses**

Intermediate analyses shall be performed the following intervals: on Days 21, 28, 42, then by Month 6 and at the end of the study.

Software SPSS shall be used for statistical analyses.

## TABLE 1. EVALUATION PROGRAM

| PERIOD | Screening Visit 1 | Baseline 2 | Visit 3 | Visit 4 | Visit 5 | Visit 6 | Visit 7 | Visit 8 | Visit 9 | Visit 10 | Visit 11 | Visit 12 | Visit 13 | Visit 14 | Visit 15 | Visit 16 | Final Visit 17 | Not programmed Early withdrawal |
|---|---|---|---|---|---|---|---|---|---|---|---|---|---|---|---|---|---|---|
| **CONTACT MEANS** | Face to face | | Telephone | | | | | | Face to face | | | | | | | | | |
| **DAY** | Day -3 | Day 0 Vaccine | Day 1 | Day 2 | Day 3 | Day 4 | Day 5 | Day 6 | Day 7 | Day 14 | Day 21 Vaccine | Day 28 (+7) | Day 35 (+14) | Day 42 (+21) | Day 90 (± 3 days) | Day 180 (± 3 days) | Day 365 (± 3 days) | |
| **GENERAL** | | | | | | | | | | | | | | | | | | |
| Informed consent | X | | | | | | | | | | | | | | | | | |
| Assignation of the volunteer number | X | | | | | | | | | | | | | | | | | |
| Selection criteria | X | X (a) | | | | | | | | | | | | | | | | |
| Clinical history, including vaccines and other drugs administered | X | | | | | | | | | | | | | | | | | |
| General physical exam | X | | | | | | | | | | | | | | | | | |
| Full vital signs (b) | X | X | | | | | | | X | X | X | X | X | X | X | X | X | X |
| Weight, height, body mass index (d) | X | X (c) | | | | | | | X | X | X | X | X | X | X | X | X | X |
| **NO PRESENCE OF SARS-CoV-2 INFECTION** | | | | | | | | | | | | | | | | | | |
| RT-PCR for SARS-CoV-2 (e) | X | | | | | | | | | | X | | | | | | | X (e) |
| IgM and IgG (e) | X | | | | | | | | | | | | | | | | | X (e) |
| Thorax CT scan | X | | | | | | | | | | | | | | | | | |
| **STUDY INTERVENTION** | | | | | | | | | | | | | | | | | | |
| Check contraindications | | X | | | | | | | | | X | | | | | | | |
| Assignation to treatment group | | X | | | | | | | | | | | | | | | | |
| Vital signs pre- and post-vaccination (f) | | X | | | | | | | | | X | | | | | | | |
| Vaccine administration | | X | | | | | | | | | X | | | | | | | |
| In observation for 90 min | | X | | | | | | | | | X | | | | | | | |
| Examination of the injection site | | X | | | | | | | X | X | X | X | X | X | X | | | |
| Deliver a new diary to the subject | | X | | | | | | | | | | | | X | | | | |
| Collect subject's diary | | | | | | | | | X | X | X | X | X | X | X | | | |
| Return subject's diary | | | | | | | | | X | X | X | X | X | | | | | |
| **IMMUNOGENICITY STUDIES** | | | | | | | | | | | | | | | | | | |
| Titers of IgM – IgG antibodies | | X | | | | | | | | X | X | X | | X | X | X | X | |
| Neutralizing antibodies | | X | | | | | | | | X | X | X | | X | X | X | X | |
| Mucosal IgA antibody | | | | | | | | | | X | X | X | | X | X | X | X | |
| T-cells response | | X | | | | | | | | X | X | X | | X | X | X | X | |
| **ADVERSE EVENTS** | | | | | | | | | | | | | | | | | | |
| Adverse events (AE), serious adverse events (SAE) and adverse events of special interest (AESI) | | X | X | X | X | X | X | X | X | X | X | X | X | X | X | X | X | X |

| | | | | | | | | | | | | | | | | | | |
|---|---|---|---|---|---|---|---|---|---|---|---|---|---|---|---|---|---|---|
| **SAFETY LABORATORY TESTS** | | | | | | | | | | | | | | | | | | |
| Complete haematology and blood chemistry panel (g) | X | | | | | | | | X | X | X | X | X | X | X | | | |
| Urine pregnancy test (h) | X | X | | | | | | | | | X | | | | | | | |
| HBsAg, Anti-HCV, Anti-HIV, VDRL | X | | | | | | | | | | | | | | | | | |
| Urinalysis | X | | | | | | | | | | X | | | | | | | |
| 12-Lead ECG | X | | | | | | | | | | | | | | | | | |
| Pulse oximetry | X | | | | | | | | | | X | | | | | | | |
| **CLINICAL SAFETY** | | | | | | | | | | | | | | | | | | |
| Evaluation of laboratory results | | X | | | | | | | X | X | X | X | X | X | X | X | | X |
| Questionnaire and physical exam | | X | | | | | | | X | X | X | X | X | X | X | X | X | X |
| Risk of SARS-CoV-2 exposure | X | X | X | X | X | X | X | X | X | X | X | X | X | X | X | X | | |
| Concomitant drugs | | X | X | X | X | X | X | X | X | X | X | X | X | X | X | X | X | X |

**NOTES:**

a. The review of inclusion and exclusion criteria will be done once the results of laboratory, clinical examinations and screening tests are completed.
b. Vital signs shall include blood pressure, heart rate, respiratory rate and body temperature measured at rest in a supine position for 5 minutes.
c. Height shall be measured only at the screening visit.
d. To calculate the body mass index, apply the formula BMI= weight [kg]/height squared [$m^2$].
e. These tests shall be carried out only in case of COVID-19 infection suspicion.
f. Full vital signs shall be measured 5 min before the vaccine administration and 30 min after.
g. Haematology tests include CBC, including differential. Blood chemistry shall include blood sugar (fasting), liver function, kidney function, blood proteins, blood fat (lipids) and basic metabolic panel.
h. Only in women with childbearing potential.

# INVESTIGATOR COMMITMENT

I ____________________________________________, the Principal Investigator, confirm that I have read, and I understand this protocol, the Investigator Manual, as well as the information of the recombinant vaccine against SARS-CoV-2 based on a viral vector of the Newcastle Disease Virus (rNDV) provided by Laboratorio Avi-Mex S.A. de C.V. (Avimex*).

I accept to conduct this investigation study according to this protocol requirements and above all, I commit to protect the rights, safety, privacy, and wellbeing of the investigation subjects by following strictly:

- The ethical principles from The Declaration of Helsinki.
- The Guideline for Good Clinical Practices (November 2016) from the International Council for Harmonization.
- Every law and regulation applicable, including laws and directives regarding data privacy, among others.
- Regulatory requirements to report serious adverse events defined in this protocol.
- Specific agreements signed between Laboratory Avi-Mex S.A. de C.V. (Avimex*) and the investigation site.

Principal Investigator: ____________________________________________

Principal Investigator Signature: ________________________________________

Date: ________________________________________________________

Investigation Unit: ________________________________________________

Phone No.: ____________________________________________________

Address: ______________________________________________________

__________________________________________________________

__________________________________________________________

## EMERGENCY CONTACT LIST

| | |
|---|---|
| Sponsors: | L.N. Ana Patricia Grajales<br>+52 (55) 5536 0663/ (55) 5543 6187<br><br>+52 55 3996 6216<br><br>ana.grajales@mccr.com.mx |
| Experts Committee: | Dr Constantino López Macías<br>+ 52 55 5437 9371<br><br>constantino@sminmunologia.mx |
| CRO: | Eng. Arturo Rodríguez<br>+52 55 5080 3620<br><br>Ext. 2004<br>+52 55 5438 8937<br><br>arturo.rodriguez@weareinfinite.mx |
| Clinical Monitor: | Ángeles Rocha Mendoza<br>+52 55 5080 3620<br>Ext. 2014 |

# ABBREVIATIONS

**A**

| | |
|---|---|
| Ab | Antibody |
| ACE2 | Angiotensin-converting enzyme 2 |
| AE | Adverse Event |
| AESI | Adverse Event of Special Interest |
| ALT | Alanine aminotransferase |
| AST | Aminotransferase aspartate |

**B**

| | |
|---|---|
| BMI | Body Mass Index |

**C**

| | |
|---|---|
| CAbT | Circulating Antibody Titre |
| CDA | Clinical data administration |
| CI | Confidence Interval |
| COVID-19 | Coronavirus Disease caused by SARS-CoV-2 |
| CPK | Creatine phosphokinase |
| CRA | Clinical Research Associate |
| CRF | Case Report Form |
| CRO | Contract Research Organization |
| CT scan | Computed Tomography scan |

**D**

| | |
|---|---|
| DCF | Data Clarification Form |
| DNA | Deoxyribonucleic acid |
| DSMB | Data Safety Monitoring Board |

**E**

| | |
|---|---|
| EID | Embryo Infectious Dose |
| ELISA | Enzyme-Linked Immunosorbent Assay |

**F**

| | |
|---|---|
| FDA | Food and Drug Administration, US |

**G**

| | |
|---|---|
| GCP | Good Clinical Practices (ICH/GCP E6 R2) |
| GMR | Geometric Mean Ratio |
| GMT | Geometric Mean Titer |

**H**

| | |
|---|---|
| Hb | Haemoglobin |
| HBsAg | Hepatitis B surface antigen |
| HBV | Hepatitis B Virus |
| HCV | Hepatitis C Virus |
| HIV | Human Immunodeficiency Virus |
| **I** | |
| ICF | Informed Consent Format |
| ICH | International Council for Harmonization |
| IgG | Immunoglobin G |
| IgM | Immunoglobin M |
| IM | Intramuscular route |
| IN | Intranasal route |
| IQR | Interquartile Range |
| IRB | Institutional Review Board |
| IU | International Unit |
| **L** | |
| Li | Lithium |
| LS | *LaSota* strain (paramyxovirus) |
| **M** | |
| MedDRA | The Medical Dictionary for Regulatory Activities |
| **N** | |
| NDV | Newcastle Disease Virus |
| **P** | |
| PCR | Polymerase Chain Reaction |
| PFU | Plaque-Forming Unit |
| PV | Pharmacovigilance |
| **R** | |
| RNA | Ribonucleic acid |
| rNDV | Recombinant (viral vector of the) Newcastle Disease Virus (against SARS-CoV-2) |
| **S** | |
| S | SARS-CoV-2 S-glycoprotein (spike) |
| SAE | Serious Adverse Event |
| SARS-CoV-2 | Severe Acute Respiratory Syndrome Coronavirus 2 |
| SD | Standard Deviation |
| **U** | |
| U/L | Unit per Litre |

**V**

| | |
|---|---|
| VDRL | Venereal Disease Research Laboratory |

**W**

| | |
|---|---|
| WBC | White Blood Cells |

**International Units**

| | |
|---|---|
| ºC | Celsius degrees |
| cm | Centimetre |
| gm | Gram |
| µL | Microlitre |

# TABLE OF CONTENT

**PROTOCOL SINOPSIS** .......... **2**

**INVESTIGATOR COMMITMENT** .......... **17**

**EMERGENCY CONTACT LIST** .......... **18**

**ABBREVIATIONS** .......... **19**

**TABLE OF CONTENT** .......... **22**

**PART I. DESIGN AND CONDUCT** .......... **26**

***1. BACKGROUND*** .......... *26*
1.1. SARS-CoV-2 background .......... 26
1.1.1. SARS-CoV-2 generalities .......... 27
1.1.2. Clinical manifestations of the disease caused by SARS-CoV-2 .......... 27
1.1.3. Newcastle Disease Virus (NDV) and its use as viral vector for vaccines development 29
1.1.4. Recombinant vaccine against SARS-CoV-2 based on a viral vector of the Newcastle Disease (rNDV) .......... 31
1.1.5. Formulation of the vaccine against SARS-CoV-2 based on a rNDV viral vector .......... 33
1.1.6. Preclinical Studies .......... 35
1.1.6.1. References of studies in animals with NDV and viral vectors based on NDV .......... 35
1.1.6.2. Preclinical studies with the recombinant active vaccine against SARS-CoV-2 based on a viral vector of the Newcastle Disease Virus (rNDV) .......... 39
1.2. Justification .......... 39

***2. STUDY OBJECTIVES*** .......... *40*
2.1 Hypothesis .......... 41

***3. STUDY DESIGN*** .......... *42*
3.1 Overall design .......... 42
3.2 Number of subjects/treatment assignation .......... 42
3.2.1 Number of subjects .......... 42
3.2.2 Assignation to vaccine and administration route .......... 42
3.3 Investigation site .......... 44
3.4 General indications .......... 44
3.5 Study period .......... 44

***4. STUDY POPULATION*** .......... *44*

4.1 Target population ... 44
4.2 Inclusion criteria ... 44
4.3 Exclusion Criteria ... 46
4.4 Withdrawal criteria ... 46

*5. STUDY PARAMETERS ... 46*
5.1 Efficacy endpoints ... 46
5.1.1. General procedure of management and processing of immunological tests ... 47
5.2 Safety endpoints ... 47
5.3.1 Adverse events ... 48
5.3.1.1. Adverse events of special interest (AESI) ... 48
5.3.1.2. Adverse Events (AEs) ... 49
5.3.1.3. Serious Adverse Events (SAEs) ... 49
5.3.2 Laboratory Tests ... 50
5.3.2.1 Haematology ... 50
5.3.2.2 Blood chemistry ... 50
5.3.3 Clinical evaluations ... 50

*6. STUDY PROCEDURES (Table 1. Evaluations program) ... 51*
6.1 Screening Visit 1 Procedures (Day -3) ... 51
6.2 Baseline Visit 2 Procedures (Day 0) ... 52
6.3 Follow-up Procedure ... 53

*7. STUDY VACCINE ... 61*
7.1 Vaccine name ... 61
7.2 Synonyms ... 61
7.3 Formula ... 61
7.3.1 Intranasal ... 61
7.3.2 Intramuscular ... 61
7.4 Concentrations ... 62
7.5 Storage and Stability ... 62
7.5.1 Stability ... 62
7.6 Packaging and Labelling ... 63
7.7 Study Vaccine ... 63
7.7.1 Dosage ... 63
7.7.2 Administration Route ... 63
7.7.3 Administration Technique ... 63
7.7.3.1 Vaccine preparation ... 64
7.7.3.2 Intranasal Administration ... 64
7.7.3.3 Intramuscular administration ... 65
7.7.3.4 Delivery and vaccine count ... 65
7.8 Concomitant Drugs ... 65

*8. Early Withdrawal ... 66*

*9. Cautions and Warnings ... 66*

*10. Statistical Considerations ... 66*
10.1 Sample Size Calculation ... 66
10.2 Statistical Analysis Plan ... 67
10.2.1 Characteristics of outcome and safety variables ... 67
10.2.2 Analysis of Comparison between Antibodies Titers and the Administration Route (IN-IN, IM-IM, IN-IM) ... 69
10.2.3 Safety Data Analysis ... 69
10.2.4 Intermediate Analyses ... 70
10.2.5 Information Processing ... 71

*11. Data Quality ... 71*

*12. Protocol Approval Signatures ... 72*

**References ... 73**

**Part II. Ethical and Administrative Considerations ... 79**

*1. Ethical considerations ... 79*
1.1. Declaration of Helsinki ... 79
1.2. Good Clinical Practices ... 79

*2. Investigator Responsibilities ... 80*
2.1 Ethics ... 80
2.1.1 Declaration of Helsinki ... 80
2.1.3 Good Clinical Practices ... 80
2.1.4 Research Ethics Committees ... 80
2.1.5 Informed Consent ... 81
2.1.6 Subject withdrawal ... 81
2.1.7 Subjects Privacy ... 82

*3. Conditions of Protocol Amendment ... 82*
3.1 Protocol Amendments ... 82
3.2 Case Report Forms ... 82
3.3 Source Document Verification ... 83

*4. Protocol Suspension Conditions ... 83*

*5. Adverse Events ... 83*
5.1 Definition ... 83
5.2 Unexpected Adverse Events ... 84
5.3 Causality ... 84
5.4 Severity of Adverse Event – Definition ... 85
5.4.1 Three-Point Scale – Definition ... 85
5.5 Treatment and Follow-up of adverse events ... 85

*6. Publication of Results and Protection of Trade Secrets ... 85*

*7. Study Documentation, Case Report Form and Other Registries* ... *86*
7.1 Special Considerations ... 86
7.2 Investigator Files/Documents Preservation ... 86
7.3. Original documents and basic data ... 87
7.4. Audits and inspections ... 87
7.5 Study Supervision ... 87
*8. Responsibilities of the Laboratory Providing the Investigational Product* ... *88*
8.1. General Responsibilities. ... 88
*9. Responsibilities of the Monitor* ... *88*
**ANNEX 1 ... 89**
**ANNEX 2 ... 92**
**ANNEX 3 ... 93**
**ANNEX 4 ... 100**
**ANNEX 5 ... 104**

# PART I. DESIGN AND CONDUCT

## 1. BACKGROUND

### 1.1. SARS-CoV-2 background

In December 2019 the first case of Severe Acute Respiratory Syndrome in Wuhan China was reported, whose aetiological agent was Coronavirus 2 (SARS-CoV-2). The coronavirus disease 2019 (COVID-19) pandemic has registered more than 100 million cases confirmed and around two point five million of deaths.[1]

In Mexico, the Ministry of Health is estimating 2,283,465 cases and about 206,463 deaths by March-2021.[2]

Coronaviruses are a large family of viruses that usually cause upper-respiratory tract illnesses, like the common cold up to severe diseases, such as the Middle East Respiratory Syndrome (MERS-CoV) and the Severe Acute Respiratory Syndrome (SARS-CoV).

The US Centres for Disease Control and Prevention (CDC) informed that a person's risk of severe illness increases in relation to age (≥65 years) and underlying medical conditions, such as chronic or moderate lung diseases, severe asthma, cardiopathies, obesity, diabetes, hypertension, chronic renal disease requiring dialysis, liver illnesses and immunosuppressed patients (CDC, 2020).

Currently, there is no treatment for COVID-19.

Regarding vaccines, there are many studies conducted by different groups, now in Phases I, II and III. To date (February 22, 2021), at least seven different vaccines derived from three technological platforms have been implemented around the world; also, there are 60 more in clinical development.

### 1.1.1. SARS-CoV-2 generalities

SARS-CoV-2 is a positive-strand RNA virus, whose genome includes 29,903 nucleotides encoding 4 structural proteins (S, E, M and N) and 6 auxiliary proteins (ORF-1, -3a, -6, -7a, -7b and -8).[4]

It has a glycoprotein membrane where S-protein (spike) is expressed. Spike protein is a homotrimer with two subdomains: SD1 and SD2. SD1 is responsible for the virulence, and it attaches the virus to the host cell through 15 residues at the Receptor Binding Domain (RBD).[5,6]

The SARS-CoV-2 RBD has a more compact conformation, and it has two critical sites to interact with the ACE2 receptor increasing virus-receptor binding affinity.[7]

The dominant antigenic epitope on SARS-CoV-2 spike-protein is RBD SD1, since this has shown immunodominance in trials conducted with a recombinant fusion protein of SARS-CoV-2 S-protein RBD, which was capable to produce neutralizing antibodies that inhibited infection in rabbits at 1:10,240 dilutions.[8]

This information highlights the importance of the spike protein as infection inducer, but also as therapeutic target to avoid it.

### 1.1.2. Clinical manifestations of the disease caused by SARS-CoV-2

SARS-CoV-2 is a virus that infects alveolar epithelial cells (pneumocytes type I and II) by an endocytosis mechanism, which allows the entry of the heterotrimer (spike protein, ACE2 receptor and BOAT-1 protein).[9,10]

However, the infection can be caused by other two mechanisms: the first one involves binding non-neutralizing antibodies derived from the individual exposure to circulating coronavirus, such as NL63, OC43, 229E or HKU1 and use FcgRIIa receptors (CD32a) expressed in epithelial cells, alveolar macrophages, and monocytes to infect them. Also, aggregation of these receptors by immunoglobulins triggers a severe inflammatory condition related to pneumonia and cytokine storm. The second mechanism involves binding DC-SIGN receptor expressed in macrophages, which may work as co-receptor of the virus and favour its appearing and replication in these immune cells.[11]

Once the virus is inside the alveolar epithelial cell, it uses cell translation mechanisms to create thousands of new viral particles. It replicates, transcribes, translates, assembles, and secretes particles outside to infect new epithelial cells.[12]

The median incubation period lasts 5 days, then the most frequent symptoms show up, such as fever, cough, nasal congestion, fatigue, and severe headache.[13]

These clinical manifestations represent the first attempt of the infected individual's immune system to remove the virus and it involves numerous inflammatory lymphocytes Th17 and T CD8 secreting perforin and granulysin, which severely damage the alveolar tissue and then favour a pro-inflammatory state characterised by inflammation and diffuse alveolar damage (DAD), bilateral consolidation areas and copious secretions comprising mucus, lymphatic fluid, and cell debris.[14,15]

It is estimated that each patient infects from 2.2 to 3.5 individuals. Infection risk, as well as severity of symptoms is higher in people ≥60 years than children.[16]

Some patients infected develop a severe local inflammatory condition in two- or three-weeks showing symptoms like pneumonia, such as dyspnoea and chest pain along with an elevation of the serum C-reactive protein (CRP) and pro-inflammatory cytokines IL-1, IL-6, IL-10 and TNF-α levels.[17]

Mortality in this stage of infection used to be higher in men older than 50 years who already have an underlying medical condition (hypertension, diabetes mellitus, cardiopathies, chronic respiratory diseases or smokers) and showing hypoxemia (59%), severe dyspnoea (71%), fever above 38ºC (92%), severe fatigue (59%) and biochemical parameters, such as neutropenia upon admission to the hospital; also, a decrease in CD4+ and CD8+ lymphocytes count, decrease of IFN-γ in CD4+ lymphocytes and still elevated levels of pro-inflammatory cytokines.[18]

### 1.1.3. Newcastle Disease Virus (NDV) and its use as viral vector for vaccines development

The Newcastle Disease Virus (NDV) is responsible for the Newcastle disease in birds and poultry. It is an avian paramyxovirus serotype 1 virus that consists of single-stranded negative-sense RNA of 15,200 bp long,[19] which does not insert in the human genome; it also has contains transcription genes (3′-N-P-M-F-HN-L-5′) encoding 6 proteins (N, P, M, F, HN and L).[20] There are three families known based on their virulence (low, intermediate and high), as well as two classes according to their genomic similarities.[21] Low-virulence non-toxic virus[21] are known as lentogenic. *LaSota* is one of the lentogenic strains widely used for avian vaccines development and for IL-2 and TRAIL production.[22,23] The replication of this strain occurs only at the inoculation site; however, it does not reach lymph nodes, but reduces induction of pro-inflammatory cytokines without affecting the robust protective immune response.[24]

Both NDV mesogenic strains, wild and reverse genetic-modified,[25] have cytolytic activity and shown to be useful against tumour cell lines derived from humans.[26,27] Since replication of this virus is regardless the host cell DNA, these strains have been used to treat several cancer types in murine models[28] and patients with colorectal cancer, in whom the cytotoxic activity of immune cells becomes easier and secondary effects (low-grade fever, conjunctivitis, laryngitis) are mild and lasts less than 24 h.[29] Nonetheless, for purposes of this study it is more relevant that this virus has been identified as a very efficient vector to express and release gene products;[30,31] and being antigenically different to the most common human pathogens, it is very unlikely the existence of circulating antibodies against NDV among population.

As abovementioned, the lentogenic *LaSota* strain is used as viral vector to express immunologically relevant antigens, since it is inoculated by intranasal route, it replicates efficiently and rapidly in the host respiratory tract and induces both local and systemic immune responses.[32,33] The NDV genomic structure allows incorporation of DNA fragments steadily expressed throughout a long process and this expression can be modulated varying the (insert) position in the vector genome.

Reverse genetics is applied to develop vaccines. With this technique the virus is fully recovered from cDNA transfecting cell lines with plasmids encoding viral compounds, including proteins related to replication and transcription.[34] Overall, insertion of a gene nearby the 3’ region between genes P and M ensures the most effective expression of the foreign protein and NDV replication.[35,36] Upon built, another advantage of this vector is its reproduction with very high titers in both eggs allantoic fluid ($10^9$ PFU/dose) and Vero cells ($10^8$ PFU/dose).

The NDV genome is non-segmented and after being transcribed and replicated a single linear RNA molecule is produced, which provides the virus with enough stability to avoid reassortment; in addition, it has proofreading polymerase (L) to maintain a low mutation rate and all these result in its genetic and antigenic stability throughout decades of use as vaccine strain.[38]

The surface glycoprotein (HN) is responsible for the attachment to the sialic acid from gangliosides and N-glycoproteins on the respiratory tract epithelial cells, where NDV infects naturally. The infection route is pH-independent, where the F protein promotes fusion between the virus enveloped and cell membrane. Besides this mechanism, the NDV can infect through cell receptor-mediated endocytosis.[39]

Following viral entry, it releases its negative-sense RNA genome into the host cell cytoplasm to be transcribed to mRNA (positive messenger), which is translated into viral proteins to assembly the virion that outside the cell carries part of the cell membrane as envelope. Infectivity of the NDV depends on the cleavage site of the F protein, synthesized as an inactive precursor (F0) and cleaved by proteases into two subunits F1 and F2. This amino acid sequence for the F protein cleavage site has been recognised as the primary factor of the infection mechanism.[40,41]

The recognition of the cell receptor by the HN protein and the neuraminidase activity determines and contributes to virulence in several avian species.[42,43,44,45]

*LaSota* strain of the NDV has a genome of 15.2 kb long and the viral vector keeps an efficient replication in chicken embryos.[46]

Vector of the NDV *LaSota* strain is capable to accommodate up to 4.5 kb of exogenous genes and express up to three proteins effectively.[32]

To produce these recombinant viruses of NDV an appropriate cell line is transfected with plasmids that include the complete genome of the virus containing the heterologous gene. The full-length NDV genome cloned is co-transfected by helper plasmids encoding viral proteins N, P and L, this under control of the bacteriophage T7 RNA polymerase promoter. The chimeric virus is recovered from the culture and propagated in SPF chicken embryos of 10 days old producing the original vaccine virus.

A main determinant of the NDV virulence is the activation of the cleavage site corresponding to the F protein precursor phenotype (fusion). For highly virulent strains cleavage is promoted by ubiquitous intracellular proteases, which allows their widespread replication in birds and poultry. However, for non-virulent or weakened strains the cleavage site is activated by a secretory protease restraining replication to the mucous surfaces and this same protease is involved with the NDV *LaSota* strain in primates and humans; therefore, NDV replication is limited to the upper respiratory tract.[47]

### 1.1.4. Recombinant vaccine against SARS-CoV-2 based on a viral vector of the Newcastle Disease (rNDV)

Recombinant vaccine against SARS-CoV-2 is a live vaccine based on the active viral vector of the Newcastle Disease Virus (rNDV) *LaSota* strain produced in SPF chicken embryos, in which the S-glycoprotein gene of the SARS-CoV-2 virus (rNDV-S) has been inserted.

Recombinant vaccine against SARS-CoV-2 is based on the design and synthesis of the SARS-CoV-2 spike antigen gene. Software Vector NTi Data Expert Tool by Thermo Scientific Fisher Mx was used for the design based on the Wuhan-Hu-1 virus sequence (NC_045512.2) and then assembled *in silico*. Construct comprises the SARS-CoV-2 (Wuhan) S-protein ectodomain, also inserted in the NDV_LS vector and fused to the transmembrane domain (TM) and the cytoplasmic tail (CT) of the NDV F protein anchored, codon-optimized for human and six prolines distributed along the spike. Vector NDVLS also carries the L289A mutation in the F protein introduced by the ISMMS.[58]

This synthetic gen also contains a single insertion site SacII at both ends and it was produced through chemical synthesis by GenScript (860 Centennial Av. Piscataway. NK. 08854. US). The synthetic Spike gene was digested by the restriction enzyme SacII (Thermo Scientific Fisher Mx) to remove it from the pUC19 plasmid (GenScript, 860 Centennial Av. Piscataway. NK. 08854. US) and subclone the fragment purified at the SacII site located between P and M genes from the *LaSota* Newcastle genome contained in pNDVLS11801140 vector. Additionally, the synthetic DNA cloned in pNDVLS11801140 vector contains all sequences of signals for transcription and translation of the S gene.

Ligation of the synthetic gene in pNDVLS11801140 vector by using T4 ligase (Thermo Fisher Scientific Mx) has produced the DNA infectious clone denominated as pNDVLS/S SARS-CoV2/TMCyto, which comprises one plasmid with the NDV genome containing the ectodomain sequence of the S gen fused to the Full-Length NDV-S (F). This infectious clone has been characterised by PCR to detect the S gene designed by using forward and reverse primers, P and M, respectively. DNA is also characterised by digestion through enzymes producing the restriction patterns expected. Finally, stability and sequence of the S gene (detected by PCR) has been confirmed by using Sanger sequencing.

From the DNA of that infectious clone the viral vector of the recombinant Newcastle Disease Virus (rNDV) is produced with the sequence of the S-glycoprotein inserted. The DNA is initially propagated inside an *E. coli* DH5α strain and purified by applying molecular biology standard procedures (Endotoxin Free-DNA Purification Kit. Quiagen). This purified DNA is used in experiments of transfection by using Lipofectamine® 3000 (Thermo Fisher Scientific Mx) in Hep-2 and A-549 cells. Twenty-four hrs after transfection the supernatant is recovered and used in viral propagation trials with 10 days old-SPF chicken embryos (Alpes, México). Embryos are incubated for 48 hrs, then the allantoic fluid is recovered to characterise production of the recombinant virus (rescued from culture cell) by haemagglutination (HA) and S gene sequence is amplified through RT-PCR with the specific P and M primers. Once the identity has been established through RT-PCR the stability of the several inserts is determined by using Sanger sequencing.

To produce the master seed new steps were followed by using Embryo Infectious Dose (EID) to calculate the viral titre for three concentrations $10^{7.0\text{-}7.49}$, $10^{7.5\text{-}7.99}$ and $10^{8.0\text{-}8.49}$ $EID_{50}$/dose.

Besides the former methods, immunoperoxidase and immunofluorescence are also useful to detect S-protein from the SARS-CoV-2 virus cloned in the NDV genome with Vero cells.

The recombinant NDV is used to infect monolayer Vero cells in serial dilutions. In 24, 48 and 72 hrs after infection the immunofluorescence method is applied to detect S-protein expressed by the recombinant NDV. Cells are fixed and challenged to primary anti-S antibody, followed by a secondary antibody coupled to immunoperoxidase. A change of colour indicates the presence of the protein.

### 1.1.5. Formulation of the vaccine against SARS-CoV-2 based on a rNDV viral vector

Recombinant vaccine against SARS-CoV-2 based on a viral vector of the Newcastle Disease Virus (rNDV) is a sterile preparation for intramuscular (IM) and intranasal (IN) administration. In both cases, IM and IN, it is a slightly turbid liquid.

Different concentrations to administer the recombinant vaccine against SARS-CoV-2 based on a viral vector of the Newcastle Disease Virus (rNDV) have been formulated with a volume of 0.5 mL through the IM route and 0.2 mL through the IN route. There are three different concentrations: $10^{7.0\text{-}7.49}$, $10^{7.5\text{-}7.99}$ and $10^{8.0\text{-}8.49}$ $EID_{50}$/dose.

The final product for IM and IN administration routes has been formulated in a vehicle that comprises one cryoprotective agent (TPG – trehalose, monobasic potassium phosphate, dibasic sodium phosphate and monosodium glutamate) plus L-Histidine for the IM route and L-Arginine and hydrolysed gelatine for the IN route. No adjuvant is used.

Filling, primary, and secondary packaging of the investigation drug product shall be performed by Avimex. The vaccine filled shall be packaged inside shipping packages validated for distribution to the investigation sites. All the investigation products shall be distributed in cold chain under refrigeration conditions to the investigation site.

Due to the manufacturing process of the recombinant vaccine against SARS-CoV-2, this has shown contain ovalbumin traces. Nevertheless, the analysis of different vaccine batches has identified the ovalbumin content below 5 µg/SD, which complies with the FEUM, and the US FDA parameters and it is still appropriate for subjects allergic to egg. The product does not contain antibiotics.

The recombinant vaccine against SARS-CoV-2 based on a viral vector of the Newcastle Disease Virus (rNDV) should be stored under refrigeration at 2-8ºC of temperature.

### 1.1.6. Preclinical Studies

#### 1.1.6.1. References of studies in animals with NDV and viral vectors based on NDV

Lentogenic strains of NDV, such as *LaSota* or B1 used as vaccine vector have demonstrated a highly weakened activity; thus, these strains result safe for poultry and for more than 70 years have been used as live vaccines in the poultry industry with good history of safety and efficacy. This characteristic is exclusive for lentogenic strains of NDV among all live vaccines. Conversely, other live vaccines currently used in human beings and animals are not naturally weakened. Therefore, it is very unlikely these NDV strains cause diseases in wild or domestic birds. In fact, studies have shown the insertion of foreign genes inside NDV genomes results in a reduced pathogenicity for birds. Thus, development of a NDV vaccine containing the SARS-CoV-2 S-gen should not imply any environmental or agricultural risk. Also, the viral vector showed no evidence of being excreted in SPF birds administered with the vaccines based on rNDV; thus, there is no horizontal nor direct contact transmission with susceptible birds. Additionally, safety tests performed with vaccines based on rNDV in SPF birds indicate that one dose 10-times higher than suggested does not cause pathogenicity issues for this type of recombinant vaccine. Following administration of the vaccine (rNDV) by intranasal route in birds a respiratory reaction similar to that induced by active vaccines containing live or active respiratory viruses occurs and disappears in around 8 days without sequels.

Buijs et al., 2014[48] described toxicity, biodistribution and excretion of the NDV injected in Cynomolgus monkeys by intravenous route. The dose-escalation design was applied to inject two animals with one non-recombinant vaccine strain, one recombinant lentogenic strain or one recombinant mesogenic strain. NDV injection did not cause severe disease, haematologic abnormalities or alteration of biochemical parameters in animals. Although injected animals excreted virus in low amounts, this did not cause seroconversion of the animals around/in contact. *Post-mortem* evaluation did not show pathological changes or evidence of viral replication. This study shows the NDV produced in chicken embryo is safe to be administered in nonhuman primates.

On the other hand, in 2005 Bukreyev et al.[49] evaluated two NDV strains as vaccine vectors: *LaSota* NDV, which replicates mainly in the respiratory tract of the chicken and BC (Beaudette) NDV of intermediate virulence that causes mild symptoms of infection in chickens. A recombinant version of each virus was modified through insertion of a gene cassette between P and M genes to encode the haemagglutinin-neuraminidase (HN) protein of the human parainfluenza virus type 3 (HPIV3), a well-known test antigen. One dose of $10^{6.5}$ PFU of recombinant viruses was administered to African green monkeys (NDV-BC and NDV-LS) and Rhesus monkeys (only NDV-BC) by intranasal and intratracheal routes combined (one

dose per site) and the same dose was administered 28 days later. Excretion of the virus or its detection in nose or throat swabbing was almost null with both strains. The lung tissue examination showed the NDV-BC strain was highly weakened and the replication pattern was confined to upper airways. Serologic response to HN increased after the second dose. These data suggest the NDV could be effective to develop vaccines against emerging human diseases and, even though one dose might be useful to control localised outbreaks, two doses would achieve a better outcome. The authors conclude that intranasal immunisation induces both local and systemic immunity, which might prevent infection or decrease its severity; also, they emphasise the vast experience from clinical tests with humans regarding administration of human paramyxovirus into the respiratory tract and they advise that have already tested vaccine vectors based on NDV would accelerate the development of a vaccine against any emerging pathogen, since it would only require the protective antigen gene cloning and its insertion into the structural base of the vector.

Additionally, a study by DiNapoli et al., 2007[50] proposes the development of a NDV vaccine against emerging diseases in humans. With the NDV vector construct, which includes an insert expressing SARS-CoV-2 S-protein, they demonstrate the vaccine administered by intranasal route gives protection to African green monkeys without causing adverse reactions. The authors also establish the required dose to be administered and determine it as a serious alternative in case of pandemics. According to that informed by the authors, the recombinant virus of NDV is appropriate to be used by the intranasal route for respiratory diseases, such as SARS-CoV-emerging. They informed the NDV 6 vector with the SARS-CoV S-gene administered by intranasal route in African green monkeys confers protection against challenge producing neutralising antibodies and mucosal immunity. Two promising candidates for vaccine were evaluated: NDV-BC/S and NDV-VF/S as one dose of $10^7$ PFU. By direct analysis of the respiratory tract tissue at the challenge virus replication peak, a significant level of immunogenicity and protective efficacy was demonstrated against SARS-CoV in a primate model. These topic candidates of vaccine for respiratory diseases resulted highly weakened in nonhuman primates without significant excretion.

Furthermore, other study by DiNapoli et al, 2007[51] offers evidence related to African green monkeys administered with a recombinant vaccine of NDV, which has been inserted with a high-pathogenic influenza gene. The authors stated that two doses of $2x10^7$ PFU administered in an interval of 28 days induced a high titre of H5N1 HPAIV (H5N1 high-pathogenic avian influenza virus) neutralising antibodies in serum of all monkeys immunised. According to that informed by the authors: *"only low titers of virus were excreted, which indicates safety. Two doses of NDV-HA induced high titers of serum H5N1 HPAIV neutralising antibodies in all monkeys immunised. Still, a significant mucosal response to immunoglobulin A was provoked in the respiratory tract after one and two doses. Titers of neutralising*

*antibodies achieved in this study suggest the vaccine could prevent mortality and reduce morbidity caused by H5N1 HPAIV virus. Also, stimulation of a local immune response in the respiratory tract is an important advantage, which very likely will reduce or avoid virus spread throughout epidemics or pandemics. This vaccine is candidate for clinical evaluation in humans".*

Another study by DiNapoli et al, 2009[52] based on the same prior SARS-CoV construct from 2007[50], demonstrated efficacy of several administration routes of NDV-vectorized vaccines in a nonhuman primate model. NDV constructs based on the Beaudette C mesogenic strain (NDV-BC), which is a version of the *LaSota* lentogenic strain, but modified to carry the polybasic furin cleavage site of NDV-BC of intermediate virulence, just between NDV-BC and NDV-*LaSota*. In this study NDV and HPIV3 seronegative results were confirmed for adult African green monkeys through hemagglutination inhibition (HI) assay by using turkey and guinea pig erythrocytes from two studies. African green monkeys were immunised with NDV-BC/HN by both intranasal and intratracheal routes combined or by subcutaneous route in one dose of $10^7$ PFU per site. On Day 28 the animals received a second dose by the same route of either, the same construct or the parental NDV-BC without insert. As negative control an additional group was immunised on days 0 and 28 with an empty vector of NDV-BC administered by intranasal, intratracheal and subcutaneous route in a dose of $10^7$ PFU per site. In total there were 4 experimental groups and one control group, each with four animals. Serum samplings were carried out on days 0, 28 and 49 to evaluate specific immune responses from vector and insert.

In a second study African green monkeys were immunised with an empty vector of NDV-BC (2 animals), NDV-BC/S (4 animals) or NDV-VF/S (4 animals) by intranasal and intratracheal routes combined on days 0 and 28 in a dose of $10^7$ PFU per site or with only $10^7$ PFU of NDV-BC/S by IN route (4 animals). Vaccination by intranasal/intratracheal route induced a good immune response, while both subcutaneous and intranasal (only one time) route induced a limited or insignificant immune response, which suggest a better response when the vaccine is administered in the lower respiratory tract. The NDV mesogenic strain (NDV-BC) vaccine compared to another of similar vector, but lentogenic strain, shared similar immunogenicity and both were equally protective.

The study by Viktorova et al., 2018[53] describes the replicative virus of NDV, which carries the genes comprised in the poliovirus capsid and when administered by intranasal route polio proteins are expressed into the host, which assemble themselves to form virus-like particles (VLP). The vaccine was tested in guinea pigs inducing VLP formation, as well as both mucosal and systemic immunity through generation of neutralising antibodies.

The study by Martinez-Sobrido, 2006[54] describes a NDV vector carrying the Respiratory Syncytial Virus (RSV) F gene, which causes death of infants and elders. The NDV is a strong inducer of alpha and beta interferon, and it also induces dendritic cells maturation. RSV reinfection is attributed to weak immunity induced by the virus. Therefore, the NDV seems an excellent choice as vaccine vector. The vaccine was tested in BLAB/c mice by intranasal route and protection against challenge, specific anti-F response and induction of specific CD8+ T-cells was observed.

Kim, 2016[32] describes a set of works performed with the NDV vector in tests of vaccines against human pathogens, in which the conclusion stated the NDV is effective to induce a strong immune response against the antigens of interest. Once again it highlights that the intranasal inoculation route has been widely tested and the doses are consistently reported. Noteworthy, NDV *LaSota* strain has been tested extensively in this type of studies producing good results, showing stability and safety, and taking advantage that there is no pre-existent immunity in human population; hence, interference with the heterologous antigen efficacy is not expected.

#### 1.1.6.2. Preclinical studies with the recombinant active vaccine against SARS-CoV-2 based on a viral vector of the Newcastle Disease Virus (rNDV)

The Icahn School of Medicine at Mount Sinai (ISMMS) carried out a study with Newcastle Disease Virus (NDV) vector vaccines expressing the SARS-CoV-2 S-protein in its wild type or in a pre-fusion membrane-anchored format.[55] The studies were performed in mice and hamster models with two vaccinations. The tested vaccines induced high levels of neutralising antibodies when the vaccine was administered intramuscularly. Importantly, these vaccine prototypes protected mice from mouse-adapted SARS-CoV-2 challenge with no detectable viral titre nor viral antigen in lungs.[55,56]

Within the information contained in the Investigator Manual preclinical studies are described in detail.

## 1.2. Justification

Absence of highly effective treatments against COVID-19 along with the effects of the pandemics regarding morbidity, mortality, and socioeconomic impact demand the development of vaccines to induce enough immune response against the infection propagation and then modify clinical development of the disease.

A literature analysis confirms that most of the strategies to develop these vaccines focus on the virus S-protein could activate the immune system most efficiently.

This study is part of the vaccine development program based on recombinant technology, which has been successfully used to elaborate avian vaccines with no contraindications against its use in humans. Manipulation of the viral vector by the reverse genetics technique we propose induces a significant immune response in the host, which includes production of neutralising antibodies against the viral S-protein and generation of immune memory that can protect the individual against new outbreaks.

The results of this study could be the foundations for the development of a useful vaccine model to reduce incidence and mortality of COVID-19 in Mexico and ultimately participate in worldwide immunisation for the pandemics control.

## 2. STUDY OBJECTIVES

**Primary Objectives:**

- To evaluate safety of three concentrations ($10^{7.0-7.49}$, $10^{7.5-7.99}$, $10^{8.0-8.49}$ $EID_{50}$/dose) of the recombinant vaccine against SARS-CoV-2 based on a viral vector of the Newcastle Disease Virus (rNDV), administered two times by intramuscular route in healthy volunteers.

- To evaluate safety of three concentrations ($10^{7.0-7.49}$, $10^{7.5-7.99}$, $10^{8.0-8.49}$ $EID_{50}$/dose) of the recombinant vaccine against SARS-CoV-2 based on a viral vector of the Newcastle Disease Virus (rNDV), administered two times by intranasal route in healthy volunteers.

- To evaluate safety of three concentrations ($10^{7.0-7.49}$, $10^{7.5-7.99}$, $10^{8.0-8.49}$ $EID_{50}$/dose) of the recombinant vaccine against SARS-CoV-2 based on a viral vector of the Newcastle Disease Virus (rNDV), administered two times by both intranasal (first) and intramuscular (second) route in healthy volunteers.

**Secondary Objectives:**

- To evaluate immunogenicity of three concentrations ($10^{7.0-7.49}$, $10^{7.5-7.99}$, $10^{8.0-8.49}$ $EID_{50}$/dose) of the recombinant vaccine against SARS-CoV-2 based on a viral vector of the Newcastle Disease Virus (rNDV), administered two times by intramuscular route in healthy volunteers.

- To evaluate immunogenicity of three concentrations ($10^{7.0-7.49}$, $10^{7.5-7.99}$, $10^{8.0-8.49}$ $EID_{50}$/dose) of the recombinant vaccine against SARS-CoV-2 based on a viral vector of the Newcastle Disease Virus (rNDV), administered two times by intranasal route in healthy volunteers.

- To evaluate immunogenicity of three concentrations ($10^{7.0-7.49}$, $10^{7.5-7.99}$, $10^{8.0-8.49}$ $EID_{50}$/dose) of the recombinant vaccine against SARS-CoV-2 based on a viral vector of the Newcastle Disease Virus (rNDV), administered two times each: first by intranasal route; and second by intramuscular route in healthy volunteers.

- To evaluate nasal mucosal humoral immunity of three concentrations ($10^{7.0-7.49}$, $10^{7.5-7.99}$, $10^{8.0-8.49}$ $EID_{50}$/dose) of the recombinant vaccine against SARS-CoV-2 based on a viral vector of the Newcastle Disease Virus (rNDV).

## 2.1 Hypothesis

- The recombinant vaccine against SARS-CoV-2 based on a viral vector of the Newcastle Disease Virus (rNDV), administered two times by intramuscular route is safe and induces humoral and cellular immune response against SARS-CoV-2 virus.

- The recombinant vaccine against SARS-CoV-2 based on a viral vector of the Newcastle Disease Virus (rNDV), administered two times by intranasal route is safe and induces humoral and cellular immune response against SARS-CoV-2 virus.

- The recombinant vaccine against SARS-CoV-2 based on a viral vector of the Newcastle Disease Virus (rNDV), administered two times each: first by intranasal route; and second by intramuscular route is safe and induces humoral and cellular immune response against SARS-CoV-2 virus.

- The recombinant vaccine against SARS-CoV-2 based on a viral vector of the Newcastle Disease Virus (rNDV), administered by intranasal route induces humoral and mucosal immune response against SARS-CoV-2 virus.

# 3. STUDY DESIGN

## 3.1 Overall design

Dose-escalation, open-label, non-randomized Phase I clinical trial which uses two administration routes to evaluate a vaccine safety and immunogenicity.

## 3.2 Number of subjects/treatment assignation

### 3.2.1 Number of subjects

90 healthy volunteers from both sexes, who comply with the selection criteria.

### 3.2.2 Assignation to vaccine and administration route

The study comprises nine treatment groups arranged to administrate three different concentrations ($10^{7.0-7.49}$, $10^{7.5-7.99}$, $10^{8.0-8.49}$ $EID_{50}$/dose) by three different administration routes (first and second by intramuscular route; first and second by intranasal route; and first administration intranasally and second intramuscularly). Each of these nine groups shall have 10 volunteers assigned, who will integrate sequentially (according to their study entry). The vaccine shall be administered on Day 0 (Baseline) and Day 21.

The following table shows how the 90 volunteers are distributed to each group according to the vaccine concentration and administration route of the first and second dose:

| **Administration route** / **Concentration** | First and second administration by intramuscular route | First and second administration by intranasal route | First administration by intranasal route and second by intramuscular route |
|---|---|---|---|
| $10^{7.0-7.49}$ $EID_{50}$/dose | 10 volunteers | 10 volunteers | 10 volunteers |
| $10^{7.5-7.99}$ $EID_{50}$/dose | 10 volunteers | 10 volunteers | 10 volunteers |
| $10^{8.0-8.49}$ $EID_{50}$/dose | 10 volunteers | 10 volunteers | 10 volunteers |

EID= embryo infectious dose.

The Data Safety Monitoring Board (DSMB) shall exhaustively assess any adverse event occurred after administration of the first vaccine dose to sentinel subjects before continuing vaccination of the other study participants. Sentinel subjects shall be distributed in groups per dose and administration route as the following table:

| | Day 1 | Day 2 | Day 3 | Day 4 | Day 5 | Day 6 | Day 7 | Day 8 | Day 9 |
|---|---|---|---|---|---|---|---|---|---|
| | Low dose ($10^{7.0\text{-}7.49}$ $EID_{50}$/dose) | | | Medium dose ($10^{7.5\text{-}7.99}$ $EID_{50}$/dose) | | | High dose ($10^{8.0\text{-}8.49}$ $EID_{50}$/dose) | | |
| IM route | Subject 1 | Subject 3 | Subject 5 | Subject 7 | Subject 9 | Subject 11 | Subject 13 | Subject 15 | Subject 17 |
| IN route | Subject 2 | Subject 4 | Subject 6 | Subject 8 | Subject 10 | Subject 12 | Subject 14 | Subject 16 | Subject 18 |
| | **Day 10 to 15** | | | | | | | | |
| | **Data Safety Monitoring Board Evaluation** | | | | | | | | |

IM= intramuscular; IN= intranasal; EID= embryo infectious dose.

Once all sentinel subjects have been vaccinated and the Data Safety Monitoring Board approved vaccination of the rest of the volunteers, the first vaccination shall be escalated according to the following table:

First administration of dose-escalated per concentration and administration route:

| Baseline dose concentration | **Day 16**<br>**Low** | **Day 17**<br>**Low** | **Day 18**<br>**Low** |
|---|---|---|---|
| IM route | S19, S21, S23 | S28, S30, S32 | S37, S38 |
| IN route | S20, S22, S24<br>S25, S26, S27 | S29, S31, S33<br>S34, S35, S36 | S39, S40,<br>S41, S42 |
| Baseline dose concentration | **Day 19**<br>**Medium** | **Day 20**<br>**Medium** | **Day 21**<br>**Medium** |
| IM route | S43, S45, S47 | S52, S54, S56 | S61<br>S62 |
| IN route | S44, S46, S48<br>S49, S50,51 | S53, S55, S57<br>S58, S59, S60 | S63, S64,<br>S65, S66 |
| Baseline dose concentration | **Day 22**<br>**High** | **Day 23**<br>**High** | **Day 24**<br>**High** |
| IM route | S67, S69, S71 | S76, S78, S80 | S85<br>S86 |
| IN route | S68, S70, S72<br>S73, S74, S75 | S77, S79, S81<br>S82, S83, S84 | S87, S88,<br>S89, S90 |

IM= intramuscular; IN= intranasal.

Administration of the second vaccine shall be on Day 21 with prior authorization of the Data Safety Monitoring Board.

### 3.3 Investigation site

The study is designed to be conducted in a clinical investigation centre. Investigators should follow the procedures described in this protocol.

### 3.4 General indications

Before the study starts the investigators and their personnel involved shall meet in order to ensure an excellent comprehension of the study and its guidelines.

### 3.5 Study period

Each subject shall participate for 12 months plus 3 days for screening.

Upon considering a healthy volunteer to participate in the study, the following appointment (at the investigation site) schedule shall be respected: Visit 1 for screening tests (day -3), Baseline (day 0), on days 1, 2, 3, 4, 5, 6 only by phone, Visit 9 (day 7), Visit 10 (day 14), Visit 11 (day 21), Visit 12 (day 28), Visit 13 (day 35) Visit 14 (day 42), Visit 15 (day 90 or month 3), Visit 16 (day 180 or month 6) and Visit 17 (day 365, month 12).

To attend appointments 1 to 14 there is a tolerance of ±24 hrs regarding the scheduled date and for appointments 15 to 17 there is a tolerance of ±3 days.

## 4. STUDY POPULATION

### 4.1 Target population

Healthy, adult volunteers from both sexes.

### 4.2 Inclusion criteria

- Adult men and women ≥18 years old and ≤55 years old.
- Signed informed consent.
- No respiratory disease within last 21 days prior to the first dose administration.
- Body Mass Index from 18.0 to 29.0 $kg/m^2$.
- Negative RT-PCR for SARS-CoV-2 infection (nasopharyngeal and oropharyngeal swabbing).
- Negative test for SARS-CoV-2 IgM and IgG antibodies.

- $O_2$ saturation ≥92% by pulse oximetry.
- Normal thorax CT scan.
- No symptoms from clinical history and normal physical examination at the screening visit.
- Lab test values within normal ranges according to the local laboratory for all the following tests:
    - Urinalysis.
    - Liver enzymes.
    - Renal function tests.
    - Cholesterol and triglycerides.
    - Fasting glucose.
    - Haematology.
- Negative tests for HBsAg, anti-HCV and anti-HIV antibodies. Negative VDRL test.
- Normal electrocardiogram.
- Negative pregnancy test for women of childbearing potential.
- Agreement of all sexually active volunteers to use highly effective contraceptives over the study period and up to 30 days after the last administration of the vaccine dose.
- Commitment from all the participants to keep social distancing, use facemask (when social distancing is not possible) and frequent handwashing with soap or antibacterial gel during the study period.

## 4.3 Exclusion Criteria

- History of hypersensitivity or allergy to any vaccine ingredient.
- History of severe anaphylactic reaction.
- History of seizures.
- History of chronic diseases or cancer.
- Vaccination against SARS-CoV-2 with either approved or experimental vaccines.
- Participation in any other study with an experimental intervention within the last 3 months.
- Administration of any other drug or herbal preparation, as well as "alternative medicine" treatments, transfer factor, chlorine dioxide or others within the last 30 days prior screening, which the investigator considers they could interfere with subject safety or data analysis.
- Any other vaccine administered within the last 30 days, including influenza vaccine.
- Fever at the time of enrolment.
- Blood transfusion or blood components transfusion within the last 4 months.
- Regular activity related to work, social interaction or entertainment that represents an exposure to SARS-CoV-2 higher than that of the general population, as per investigator judgement.
- Poultry farm work, including contact with fighting cocks.
- Alcohol and drugs abuse, which may interfere with the development of the research protocol.
- Any medical or not medical condition that could threaten patient safety or interfere with vaccine evaluation or analysis of the study results.

## 4.4 Withdrawal criteria

- Pregnancy.
- Conditions that lead the Investigator to suspend the study.
- Treatment non-compliance that threatens validity of the study, as deliberated by the Investigator/Sponsor.

# 5. STUDY PARAMETERS

## 5.1 Efficacy endpoints

- Circulating anti-SARS-CoV-2 antibodies titers. A seroconversion is defined as the appearance of specific circulating antibodies (IgG and IgM) titers for SARS-CoV-2 S-protein epitopes determined by the immunoassay ELISA.
- Neutralizing antibodies titers.

- IFN-γ production by T-cell immunity response after spike protein challenge, shall be assessed by ELISPOT assay and proliferation of cell subpopulations by FC.
- IgA titers in nasal mucosa.

#### 5.1.1. General procedure of management and processing of immunological tests

Immunological studies shall be conducted in the National Institute of Respiratory Diseases (INER) Tuberculosis Immunology Laboratory, Flow Cytometry Unit and Clinical Microbiology Laboratory.

At the Hospital *Médica Sur* (clinical investigation centre) the procedure of drawing a peripheral blood sample from each participant according to the scheduled appointment shall be performed to proceed with the immunogenicity analysis. Blood samples and safety samples are drawn at the same time.

Three test tubes are necessary for immunological tests: 1 tube without anticoagulant, 1 tube with sodium heparin and 1 tube with CPA. For volunteers assigned to intranasal vaccine the sample from nasal swabbing shall be contained inside a test tube (to determine IgA). Tubes containing peripheral blood shall be sent to the INER inside a container identified, at room temperature and under proper conditions for biological samples transport, which shall be delegated to an accredited courier for biological samples.

Presence of circulating anti-spike antibodies (IgG, IgM and IgA), as well as neutralising antibodies shall be confirmed by ELISA. Incidence of T-cells expressing IFN-γ shall be determined by ELISPOT. Specific antigen expression from several cytokines in peripheral blood mononuclear cells culture supernatant shall be assessed by Bioplex. Cell phenotype and intracellular cytokines production shall be determined by flow cytometry.

For 5 years a set of aliquots from each volunteer shall be safeguarded under freezing conditions (-20 and -70ºC), since such samples are Sponsor's property. It is likely that new technology or new discoveries generate questions; therefore, volunteers were requested to sign their informed consent to use their blood samples in further investigations to enrich knowledge about SARS-CoV-2 or COVID-19 behaviour.

### 5.2 Safety endpoints

- Adverse Events of Special Interest (AESIs), as defined in this protocol.
- Adverse Events (AEs), as defined by the GPC – ICH.
- Serious Adverse Events (GCP classification).
- Laboratory tests.
    - Haematology: complete blood count, including differential.

  - Blood chemistry: blood sugar, liver enzymes, renal enzymes, metabolic and lipid panel.

- Clinical evaluations of signs and symptoms.

### 5.3.1 Adverse events

There are three categories of adverse events for this study:

#### 5.3.1.1. Adverse events of special interest (AESI)

**Derived from vaccination**

- Injection site inflammation.
- Injection site redness.
- Injection site pruritus.
- Elevation of temperature at the injection site.
- Low-grade fever.

**COVID-19 related**

- Fever or chills.
- Cough.
- Shortness of breath.
- Muscle and joint pain.
- Headache.
- Loss of taste or smell.
- Sore throat.
- Congestion or runny nose.
- Nauseas or vomit.
- Diarrhoea.
- Diagnosis related to COVID-19.
- Fatigue.

#### 5.3.1.2. Adverse Events (AEs)

An adverse event is defined as any undesirable experience that starts after study intervention, whether considered related to it. AEs include signs, symptoms, intercurrent diseases and laboratory alterations occurring throughout clinical study or if present since the screening test their severity or frequency increase.

Every adverse event occurred throughout study shall be registered in the clinical record and its corresponding Case Report Form. At every visit, patients shall be encouraged to report all new sign, symptom or change experienced since their last visit. All changes should be investigated and registered properly.

Adverse events are categorised with a three-point scale and evaluated as causal related or not with treatment (see Annex 1).

#### 5.3.1.3. Serious Adverse Events (SAEs)

Serious adverse events are those fulfilling the following criteria:

- Result in subject death (death by itself should be considered an outcome, not an event).
- Life threatening, which refers an event where patient was at substantial risk of dying and not an event that hypothetically could cause subject’s death if were more severe.
- Require hospitalization or its prolongation (unexpected).
- With disability or permanent damage.
- Congenital anomaly, birth defects.

- Require intervention to prevent any prior outcome.

### 5.3.2 Laboratory Tests

#### 5.3.2.1 Haematology

Haemoglobin, erythrocytes, haematocrit, platelets, leukocytes and differential panel (neutrophiles, lymphocytes, monocytes, eosinophiles, basophiles).

#### 5.3.2.2 Blood chemistry

Fasting blood sugar, creatinine, BUN, total, direct and indirect bilirubin, TGO, TGP, alkaline phosphatase, DHL, GGT, albumin, total proteins, lipids panel and metabolic panel.

### 5.3.3 Clinical evaluations

Every Visit includes a general questionnaire, vital signs measuring, general physical examination, injection site assessment and collect of information regarding concomitant drugs used.

If patient is withdrawn from study (whatever reason), at least one further Visit shall be scheduled as Final Visit.

# 6. STUDY PROCEDURES (Table 1. Evaluations program)

## 6.1 Screening Visit 1 Procedures (Day -3)

At the Screening Visit the first step is to obtain the informed consent signed. No other screening procedure could be performed to any volunteer unless the informed consent has been signed in duplicate. And one copy should be given. It is important to have the subject and his/her contact person phone number.

Once the subject has signed the informed consent the following procedures shall be conducted:

- Subject shall be assigned with an identification number for this study according to the sequential assignation table.
- Inclusion and exclusion criteria shall be matched.
- Complete clinical history including demographic data, age, sex, race (ethnic origin), work, risk factors to develop severe COVID-19, vaccines history (highlighting influenza vaccine and other in adulthood) and usual activities that lead to greater exposure to SARS-CoV-2.
- Physical examination, including vital signs measured after 5 min of rest (blood pressure, heart rate, respiratory rate, oxygen saturation and body temperature); and weight and height calculating body mass index.
- Drug products used by the volunteer within 30 days shall be registered.
- RT-PCR to detect SARS-CoV-2 IgG and IgM shall be performed.
- Tests of VDRL, HBsAg, antibodies against HCV, HIV, and urinalysis.
- Pregnancy tests for women of childbearing potential.
- ECG and thorax CT scan.
- Qualitative analysis of COVID-19 exposure risk by using table from Annex 5 as support.
- Schedule Baseline Visit within three business days.
- Blood sampling for haematology tests, fasting blood sugar, creatinine, BUN, total, direct and indirect bilirubin, TGO, TGP, alkaline phosphatase, DHL, GGT, albumin, total proteins, lipids panel and metabolic panel.

## 6.2 Baseline Visit 2 Procedures (Day 0)

The following is necessary throughout Visit 2 (Baseline Visit):

- Ask subject for adverse events occurred to be evaluated and SARS-CoV-2 infection risk.
- Physical examination, complete vital signs after 5 min of rest and register subject's weight.
- Urine pregnancy tests for women of childbearing potential.
- Check laboratory and clinical tests results from the Screening Test.
- Match inclusion and exclusion criteria.
- Register concomitant drugs used.

Should volunteer fulfil all inclusion criteria (and no exclusion criteria) an overall evaluation shall confirm there are no contraindications for vaccine administration. With no contraindications, samples shall be drawn for baseline immunology tests, including SARS-CoV-2 IgG and IgM titers, neutralising antibodies titers and T-cells immune response.

With the full information of each volunteer, each shall be distributed in nine groups based on vaccine concentration and administration route. The assigned vaccine shall be prepared following the instructions:

- Check contraindications.
- Measure vital signs before vaccination.
- Apply the vaccine according to its assignation.
- Keep the volunteer in observation for 30 min, after which vital signs shall be measured again followed by 60 min of observation.
- Check the injection site 90 min after vaccine administration.
- Register the procedure in the clinical record in detail.

At the end of the observation period after applying the vaccine the subject shall receive a diary along with the instructions to fill. At Visit 2 subject should bring the diary to receive instructions about warning signs.

## 6.3 Follow-up Procedure

**Visits 3, 4, 5, 6, 7 and 8. Phone contact (Days 1, 2, 3, 4, 5, 6)**

From Day 1 to 6 the volunteer shall be contacted by phone after applying the vaccine to evaluate his/her condition and ask for adverse events, adverse events of special interest and serious adverse events occurred, as well as use of concomitant drugs and activities that increase his/her SARS-CoV-2 exposure risk.

Information should be documented in the clinical record.

**Visit 9 (Day 7)**

Procedures to be performed:

- Clinical evaluation, including questions about adverse events and activities that represent a higher SARS-CoV-2 exposure risk.
- Ask the subject about concomitant drugs used and register the information in the clinical record.
- Physical examination, measure vital signs after 5 min of rest, as well as subject weight and check the injection site.
- Request the diary to check if the information provided is registered therein.
- Check the laboratory results from the prior visit (screening).
- Draw blood samples for haematology tests, fasting blood sugar, creatinine, BUN, total, direct and indirect bilirubin, TGO, TGP, alkaline phosphatase, DHL, GGT, albumin, total proteins, lipids panel and metabolic panel.
- Return the diary to the volunteer and provide him/her with general indications.
- Evaluate qualitatively his/her COVID-19 exposure risk by using the Annex 5 table as support.

### Visit 10 (Day 14)

Procedures to be performed:

- Clinical evaluation, including questions about adverse events and activities that represent a higher SARS-CoV-2 exposure risk.
- Ask the subject about concomitant drugs used and register the information in the clinical record.
- Physical examination, measure vital signs after 5 min of rest, as well as subject weight and check the injection site.
- Request the diary to check if the information provided is registered therein.
- Check the laboratory results from the prior Visit 9.
- Draw blood samples for haematology tests, fasting blood sugar, creatinine, BUN, total, direct and indirect bilirubin, TGO, TGP, alkaline phosphatase, DHL, GGT, albumin, total proteins, lipids panel and metabolic panel.
- Draw blood samples for antibodies and neutralising antibodies titers, and T-cell tests. Also, collect a sample from nasal discharge according to immunological tests indications.
- Evaluate qualitatively his/her COVID-19 exposure risk by using the Annex 5 table as support.
- Return the diary to the volunteer and provide him/her with general indications. Also, remind the volunteer the second vaccine shall be applied the next Visit.

### Visit 11 (Day 21) for 2nd Vaccination

Procedures to be performed:

- Clinical evaluation, including questions about adverse events and activities that represent a higher SARS-CoV-2 exposure risk.
- Ask the subject about concomitant drugs used and register the information in the clinical record.
- Physical examination, measure vital signs after 5 min of rest, as well as subject weight, check the injection site and register oxygen saturation values.
- Request the diary to check if the information provided is registered therein.
- Check the laboratory results from the prior Visit 10.
- Evaluate qualitatively his/her COVID-19 exposure risk by using the Annex 5 table as support.
- Before the second vaccination draw blood samples for haematology tests, fasting blood sugar, creatinine, BUN, total, direct and indirect bilirubin, TGO, TGP, alkaline phosphatase, DHL, GGT, albumin, total proteins, lipids panel and metabolic panel.

- Collect samples of urine for uranalysis and pregnancy tests for women of childbearing potential.
- Perform PCR for SARS-CoV-2. Draw blood samples for immune tests of Day 21, which include SARS-CoV-2 IgG and IgM and neutralising antibodies titers and T-cells immune response.
- Before second vaccination collect a nasal discharge sample according to immunological tests indications.

Once the study physician declares there are no contraindications to apply the vaccine on the volunteer, the vaccine is prepared and applied according to concentration and route administration assigned. This process includes:

- Measure vital signs before vaccination.
- Apply the vaccine following its dose and administration route assigned.
- Keep the volunteer in observation for 30 min. Measure vital signs and again keep him/her in observation for 60 min.
- Check the injection site 90 min after vaccination.
- Register the procedure in the medical record in detail.

At the end of the observation period after vaccination the subject shall receive a diary along with the instructions to fill. At Visit 12 subject should bring the diary to receive instructions about warning signs.

**Visit 12 (Day 28)**

Procedures to be performed:

- Clinical evaluation, including questions about adverse events and activities that represent a higher SARS-CoV-2 exposure risk.
- Ask the subject about concomitant drugs used and register the information in the clinical record.
- Physical examination, measure vital signs after 5 min of rest, as well as subject weight and check the injection site.
- Request the diary to check if the information provided is registered therein.
- Check the laboratory results from Visit 11.
- Draw blood samples for haematology tests, fasting blood sugar, creatinine, BUN, total, direct and indirect bilirubin, TGO, TGP, alkaline phosphatase, DHL, GGT, albumin, total proteins, lipids panel and metabolic panel.
- Draw blood samples for antibodies and neutralising antibodies titers and T-cell tests.
- Collect a sample from nasal discharge according to immunological tests indications.

- Evaluate qualitatively his/her COVID-19 exposure risk by using the Annex 5 table as support.
- Return the diary to the volunteer and provide him/her with general indications, as well as warning signs.

**Visit 13 (Day 35)**

Procedures to be performed:

- Clinical evaluation, including questions about adverse events and activities that represent a higher SARS-CoV-2 exposure risk.
- Ask the subject about concomitant drugs used and register the information in the clinical record.
- Physical examination, measure vital signs after 5 min of rest, as well as subject weight and check the injection site.
- Request the diary to check if the information provided is registered therein.
- Check the laboratory results from Visit 12.
- Draw blood samples for haematology tests, fasting blood sugar, creatinine, BUN, total, direct and indirect bilirubin, TGO, TGP, alkaline phosphatase, DHL, GGT, albumin, total proteins, lipids panel and metabolic panel.
- Evaluate qualitatively his/her COVID-19 exposure risk by using the Annex 5 table as support.
- Return the diary to the volunteer and provide him/her with general indications.

### Visit 14 (Day 42)

Procedures to be performed:

- Clinical evaluation, including questions about adverse events and activities or situations that represent a SARS-CoV-2 infection risk.
- Ask the subject about concomitant drugs used and register the information in the clinical record.
- Physical examination, measure vital signs after 5 min of rest, as well as subject weight and check the injection site.
- Request the diary to check if the information provided is registered therein. This diary shall be safeguarded in the clinical record.
- Check the laboratory results from Visit 13.
- Draw blood samples for haematology tests, fasting blood sugar, creatinine, BUN, total, direct and indirect bilirubin, TGO, TGP, alkaline phosphatase, DHL, GGT, albumin, total proteins, lipids panel and metabolic panel. Also, draw blood samples for antibodies and neutralising antibodies titers and T-cell tests.
- Collect a sample from nasal discharge according to immunological tests indications.
- Evaluate qualitatively his/her COVID-19 exposure risk by using the Annex 5 table as support.
- Provide the volunteer with a new diary and general indications. Also, remind the volunteer to bring that diary the next Visit.

### Visit 15 (Month 3, Day 90)

Procedures to be performed:

- Clinical evaluation, including questions about adverse events and activities or situations that represent a SARS-CoV-2 infection risk.
- Ask the subject about concomitant drugs used and register the information in the clinical record.
- Physical examination, measure vital signs after 5 min of rest, as well as subject weight and check the injection site.
- Request the diary to check if the information provided is registered therein. This diary shall be safeguarded in the clinical record; hence, no diary is returned.
- Check the laboratory results from Visit 14.
- Draw blood samples for haematology tests, fasting blood sugar, creatinine, BUN, total, direct and indirect bilirubin, TGO, TGP, alkaline phosphatase, DHL, GGT, albumin, total proteins, lipids panel and metabolic panel. Also, draw blood samples for antibodies and neutralising antibodies titers and T-cell tests.

- Collect a sample from nasal discharge according to immunological tests indications.
- Evaluate qualitatively his/her COVID-19 exposure risk by using the Annex 5 table as support.
- Provide the volunteer with general indications.

**Visit 16 (Month 6, Day 180)**

Procedures to be performed:

- Clinical evaluation, including questions about adverse events and activities or situations that represent a SARS-CoV-2 infection risk.
- Ask the subject about concomitant drugs used and register the information in the clinical record.
- Physical examination, measure vital signs after 5 min of rest, as well as subject weight.
- Check the laboratory results from Visit 15 (Month 3).
- Draw blood samples for antibodies, neutralising antibodies titers and T-cell tests.
- Collect a sample from nasal discharge according to immunological tests indications.
- Evaluate qualitatively his/her COVID-19 exposure risk by using the Annex 5 table as support.
- Provide the volunteer with general indications.

**Final Visit 17 (Month 12, Day 360)**

Procedures to be performed:

- Clinical evaluation, including questions about adverse events.
- Ask the subject about concomitant drugs used and register the information in the clinical record.
- Physical examination, measure vital signs after 5 min of rest, as well as subject weight.
- Check the laboratory results from Visit 15 (Month 3).
- Draw blood samples for antibodies, neutralising antibodies titers and T-cell tests; an also collect a sample from nasal discharge from volunteers who received the intranasal vaccine according to the immunology tests indications.
- The volunteer should be informed this is the last visit and possibly the investigation personnel may contact him/her further, in case of need more data or communicate relevant information.

## TABLE 1. EVALUATION PROGRAM

| PERIOD | Screening Visit 1 | Baseline 2 | Visit 3 | Visit 4 | Visit 5 | Visit 6 | Visit 7 | Visit 8 | Visit 9 | Visit 10 | Visit 11 | Visit 12 | Visit 13 | Visit 14 | Visit 15 | Visit 16 | Final Visit 17 | Not programmed Early withdrawal |
|---|---|---|---|---|---|---|---|---|---|---|---|---|---|---|---|---|---|---|
| CONTACT MEANS | Face to face | | Telephone | | | | | | Face to face | | | | | | | | | |
| DAY | Day -3 | Day 0 Vaccine | Day 1 | Day 2 | Day 3 | Day 4 | Day 5 | Day 6 | Day 7 | Day 14 | Day 21 Vaccine | Day 28 (+7) | Day 35 (+14) | Day 42 (+21) | Day 90 (± 3 days) | Day 180 (± 3 days) | Day 365 (± 3 days) | |
| **GENERAL** | | | | | | | | | | | | | | | | | | |
| Informed consent | X | | | | | | | | | | | | | | | | | |
| Assignation of the volunteer number | X | | | | | | | | | | | | | | | | | |
| Selection criteria | X | X (a) | | | | | | | | | | | | | | | | |
| Clinical history, including vaccines and other drugs administered | X | | | | | | | | | | | | | | | | | |
| General physical exam | X | | | | | | | | | | | | | | | | | |
| Full vital signs (b) | X | X | | | | | | | X | X | X | X | X | X | X | X | X | X |
| Weight, height, body mass index (d) | X | X (c) | | | | | | | X | X | X | X | X | X | X | X | X | X |
| **NO PRESENCE OF SARS-CoV-2 INFECTION** | | | | | | | | | | | | | | | | | | |
| RT-PCR for SARS-CoV-2 (e) | X | | | | | | | | | | X | | | | | | | X (e) |
| IgM and IgG (e) | X | | | | | | | | | | | | | | | | | X (e) |
| Thorax CT scan | X | | | | | | | | | | | | | | | | | |
| **STUDY INTERVENTION** | | | | | | | | | | | | | | | | | | |
| Check contraindications | | X | | | | | | | | | X | | | | | | | |
| Assignation to treatment group | | X | | | | | | | | | | | | | | | | |
| Vital signs pre- and post-vaccination (f) | | X | | | | | | | | | X | | | | | | | |
| Vaccine administration | | X | | | | | | | | | X | | | | | | | |
| In observation for 90 min | | X | | | | | | | | | X | | | | | | | |
| Examination of the injection site | | X | | | | | | | X | X | X | X | X | X | X | | | |
| Deliver a new diary to the subject | | X | | | | | | | | | | | | X | | | | |
| Collect subject's diary | | | | | | | | | X | X | X | X | X | X | X | | | |
| Return subject's diary | | | | | | | | | X | X | X | X | X | | | | | |
| **IMMUNOGENICITY STUDIES** | | | | | | | | | | | | | | | | | | |
| Titers of IgM – IgG antibodies | | X | | | | | | | | X | X | X | | X | X | X | X | |
| Neutralizing antibodies | | X | | | | | | | | X | X | X | | X | X | X | X | |
| Mucosal IgA antibody | | | | | | | | | | X | X | X | | X | X | X | X | |
| T-cells response | | X | | | | | | | | X | X | X | | X | X | X | X | |
| **ADVERSE EVENTS** | | | | | | | | | | | | | | | | | | |
| Adverse events (AE), serious adverse events (SAE) and adverse events of special interest (AESI) | | X | X | X | X | X | X | X | X | X | X | X | X | X | X | X | X | X |
| **SAFETY LABORATORY TESTS** | | | | | | | | | | | | | | | | | | |
| Complete haematology and blood chemistry panel (g) | X | | | | | | | | X | X | X | X | X | X | X | | | |
| Urine pregnancy test (h) | X | X | | | | | | | | | X | | | | | | | |
| HBsAg, Anti-HCV, Anti-HIV, VDRL | X | | | | | | | | | | | | | | | | | |

| | | | | | | | | | | | | | | | | | | |
|---|---|---|---|---|---|---|---|---|---|---|---|---|---|---|---|---|---|---|
| Urinalysis | X | | | | | | | | | | X | | | | | | | |
| 12-Lead ECG | X | | | | | | | | | | | | | | | | | |
| Pulse oximetry | X | | | | | | | | | | X | | | | | | | |
| **CLINICAL SAFETY** | | | | | | | | | | | | | | | | | | |
| Evaluation of laboratory results | | X | | | | | | | X | X | X | X | X | X | X | X | | X |
| Questionnaire and physical exam | | X | | | | | | | X | X | X | X | X | X | X | X | X | X |
| Risk of SARS-CoV-2 exposure | X | X | X | X | X | X | X | X | X | X | X | X | X | X | X | X | | |
| Concomitant drugs | | X | X | X | X | X | X | X | X | X | X | X | X | X | X | X | X | X |

**NOTES:**

a. Check inclusion and exclusion criteria once the results of laboratory, clinical examinations and screening tests are completed.
b. Vital signs shall include blood pressure, heart rate, respiratory rate and body temperature measured at rest in a supine position for 5 minutes.
c. Height shall be measured only at the screening visit.
d. To calculate the body mass index, apply the formula BMI= weight [kg]/height squared [$m^2$].
e. These tests shall be carried out only in case of COVID-19 infection suspicion.
f. Full vital signs shall be measured 5 min before the vaccine administration and 30 min after.
g. Haematology tests include CBC, including differential. Blood chemistry shall include blood sugar (fasting), liver function, kidney function, blood proteins, blood fat (lipids) and basic metabolic panel.
h. Only in women of childbearing potential.

# 7. STUDY VACCINE

## 7.1 Vaccine name

AVX/COVID-12.

## 7.2 Synonyms

Active viral vector vaccine containing SARS-CoV-2 spike-protein insert.

## 7.3 Formula

### 7.3.1 Intranasal

Formula: Each 0.2 mL contains:

| | |
|---|---|
| Purified FAA-rNDV-LS-S-HP | NLT 0.032 mL |
| Stabiliser (TPG/L-Arginine/Hydrolysed gelatine solution) | NMT 0.168 mL |
| **Total** | **0.2 mL** |

To produce one batch for investigation studies with 2,500 pieces and a filling volume of 0.2 mL:

| | |
|---|---|
| Purified FAA-rNDV-LS-S-HP | NLT 80.0 mL |
| Stabiliser (TPG/L-Arginine/Hydrolysed gelatine solution) | NMT 420.0 mL |
| **Total** | **500.0 mL** |

### 7.3.2 Intramuscular

Formula: Each 0.5 mL contains:

| | |
|---|---|
| Purified FAA-rNDV-LS-S-HP | NLT 0.032 mL |
| Stabiliser (TPG/L-Histidine solution) | NMT 0.468 mL |

| | |
|---|---|
| **Total** | **0.5 mL** |

To produce one batch for investigation studies with 1,000 pieces and a filling volume of 0.5 mL (500 mL):

| | |
|---|---|
| Purified FAA-rNDV-LS-S-HP | NLT 32.0 mL |
| Stabiliser (TPG/L-Histidine solution) | NMT 468.0 mL |
| **Total** | **500.0 mL** |

The amount of FAA varies depending on the titre, but not less than 5%.

Minimum FAA titre (diafiltered) included in the finished product formulation is $10^7$ $EID_{50}$/dose. Titre may change, it depends on the clinical study results.

## 7.4 Concentrations

$10^{7.0-7.49}$, $10^{7.5-7.99}$ and $10^{8.0-8.49}$ $EID_{50}$/dose.

## 7.5 Storage and Stability

### 7.5.1 Stability

Based on that established in the NOM-073-SSA1-2015, there is a Stability Study Procedure (PNOH-Q-013) to carry out the long-term study under refrigeration (from 2 – 8ºC) and freezing (-20ºC) conditions with the finished product.

### 7.5.2 Storage

The vaccine should be stored refrigerated at 2 to 8°C.

## 7.6 Packaging and Labelling

For the product it is intended to use sterile borosilicate type 1 vials as primary package for clinical studies. These are washed, depyrogenated and sterilized primary glass containers for pharmaceutical use, which should protect against humidity and microbial contamination.

Type of container-closure system used for the pharmaceutical form and diluent reconstituted, if apply.

| No. | Specification No. | Material name/Raw material | Grade | Intended for | |
|---|---|---|---|---|---|
| 1 | EMVPB001 | Borosilicate T-1 bottle | Type 1 | Packaging | Filling |
| 2 | EMVPB002 | Bromo-butyl T-1 stopper of 13 mm | Type 1 | Packaging | Stoppering |
| 3 | EMVPB003 | Lacquered aluminium cap of 13 mm | Lacquered aluminium | Packaging | Capping |
| 4 | EMVPB004 | Sterile single-use bag | Sartorius | Safeguard formulated and filtered product | Formulation and filtration |

Vaccine should be labelled as investigation product bearing the administration route, volume, and vial number.

## 7.7 Study Vaccine

### 7.7.1 Dosage

Intranasal: 0.2 mL containing $1x10^{7.0\text{-}7.49}$, $1x10^{7.5\text{-}7.99}$ and $1x10^{8.0\text{-}8.49}$ $EID_{50}$/dose.
Intramuscular: 0.5 mL containing $1x10^{7.0\text{-}7.49}$, $1x10^{7.5\text{-}7.99}$ and $1x10^{8.0\text{-}8.49}$ $EID_{50}$/dose.

### 7.7.2 Administration Route

Intranasal and intramuscular.

### 7.7.3 Administration Technique

#### 7.7.3.1 Vaccine preparation

- To start thawing process vials should be transported under refrigeration conditions (2-8°C) for 14 and 10 hrs before use.
- Vial to be used should reach room temperature maximum 30 min before application.
- Once vial has reached room temperature IT SHOULD NOT FREEZE NOR REFRIGERATE AGAIN for further use.

#### 7.7.3.2 Intranasal Administration

Fill the syringe with the vial containing the vaccine AVX/COVID-12 (0.2 mL).

Administer each nostril with 0.1 mL (half and half) as illustrated below.

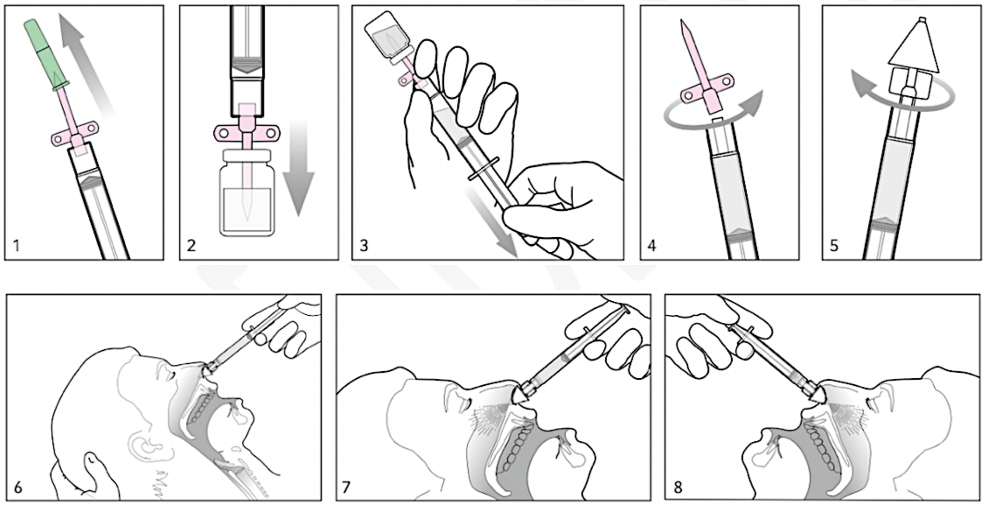


Instructions:

1. Remove and discard the green cover that protects the adaptor with needle.
2. Introduce the adaptor needle into the vial containing the vaccine.
3. Pull the plunger to charge the syringe with an appropriate volume to provide the patient with 0.2 mL.
4. Remove the adaptor with needle rotating it and discard it in an appropriate sharps and infectious wastes container.
5. Attach the MAD device to the syringe through the internal connector.

6. With the free hand hold patient's head by the occipital area and place the MAD device tip comfortably inside one nostril pointing slightly upwards (towards the top of the ear).
7. Press the syringe plunger intensely to release half of the vaccine inside one nostril.
8. Then, move to the other nostril and repeat steps 6 and 7.

After vaccination discard the syringe with the device according to the procedures established to manage biohazardous wastes. The container should be preserved to count vials according to the investigation unit guidelines.

#### 7.7.3.3 Intramuscular administration

- Charge the syringe with the bottle containing the vaccine AVX/COVID-12 (0.5 mL).
- Clean the injection site properly, which is the area of the deltoid muscle from the non-dominant arm.
- Administer the syringe content intramuscularly.
- Preserve the container to count vials according to the investigation unit guidelines.

#### 7.7.3.4 Delivery and vaccine count

Vaccine shall be distributed on a weekly basis in cold chain under refrigeration conditions (2-8ºC) to keep its stability and safeguarded at the investigation site under refrigeration conditions (2-8ºC) too.

The number of vaccines received is registered at the investigation site and stored inside the refrigerator assigned. The Study Monitor shall compare the vials reported with those delivered and those keep locked.

## 7.8 Concomitant Drugs

All concomitant drugs shall appear in the Case Report Forms. There are two types of drugs: those administered within 30 days before Baseline Visit (listed in the Drugs History section) and those indicated after vaccine assignation, which are registered as concomitant drugs. In both cases the generic names (although for those having multiple compounds is preferable to use their tradename), start date, end date or if continue shall be registered.

It is important to annotate every drug product applied or consumed, regardless of who prescribed it.

# 8. Early Withdrawal

Early withdrawal of a patient could be due to any of these reasons:

- **Investigator decision to protect subject from risks duly confirmed.**
- **Subject decision.** Volunteers can leave the study any time and due to any reason. It is always desirable to request an explanation to leave the study but clarifying this is not mandatory.

If early withdrawal of a subject is unavoidable, every effort should be made to attend the visit indicated by the procedures schedule in case of early withdrawal and document the observations in detail.

# 9. Cautions and Warnings

In Mexico, the operation of a national vaccination program against COVID-19 has started by using vaccines approved by COFEPRIS for emergency use. Because of this, it is important to explain the volunteer that if he/she considers receiving any of these vaccines, such situation should be consulted with the Principal Investigator, since the interaction between this experimental vaccine with that other against COVID-19 is unknown.

# 10. Statistical Considerations

## 10.1 Sample Size Calculation

Sample size calculation has not been performed. 10 healthy volunteers are estimated per each of the nine groups organized according to dose and administration route with a total sample of 90 healthy volunteers.

## 10.2 Statistical Analysis Plan

To evaluate vaccine groups and administration route statistical tests shall apply to each group regardless of similar baseline characteristics. Student-*t* test and ANOVA shall be applied to equality of means in continuous variables and non-parametric tests to equality of medians in count variables.

### 10.2.1 Characteristics of outcome and safety variables

**Immunogenicity**

Titers of SARS-CoV-2 anti-spike protein antibodies (IgG, IgM and IgA) shall be summarised and analysed for all subjects per treatment group, according to the following:

SARS-CoV-2 anti-spike protein antibodies shall be expressed as Geometric Mean Titer (GMT) with a 95% CI.

Geometric Mean Titer and its two-sided 95% Confidence Interval (CI) shall be calculated the following days: 0 (Baseline), 14 and 21, 28, 35, 42, 90, 180 and 365.

ANOVA shall be used to analyse log-transformed antibody titers and the Wilcoxon rank-sum test for non-normal distribution data.

When there is a significant overall difference among the three concentration groups paired comparisons shall be performed and the differences between groups shall be estimated with 95% CI.

Two-sided 95% CI for GMT shall be calculated by back-transformation of the 95% confidence limits based on a Student-*t* test for titrations with $\log_{10}$-transformation.

Ratio of subjects with a titration rate above the parameter established for IgG, IgM and IgA antibodies with 95% CI shall be measured the following days: 14, 21, 28, 35, 42, 90, 180 and 365.

The percentage of subjects showing IgG, IgM and IgA antibodies and their corresponding two-sided 95% CI shall be reported.

For presence of each antibody (IgG, IgM and IgA) the CI shall be found by using the F distribution method given in Collett (Collett, 1991).

Seroconversion rate and 95% CI shall be determined on Day 14 or 21 regarding Day 0 of vaccination. Seroconversion is defined as the appearance of specific circulating antibodies (IgG, IgM and IgA) titers for SARS-CoV-2 S-protein epitopes determined by the immunoassay ELISA.

Total percentage of subjects showing seroconversion and their corresponding two-sided 95% CI shall be reported. Analyses shall be performed for all subjects and separately (seronegative and seropositive subgroups).

Geometric Mean Ratio (GMR) and its 95% CI for ratios of Day 14 or 21 are divided by the antibodies (IgG and IgM) appearance on Day 1. The GMR and its two-sided 95% CI shall be summarised. GMR CI shall be calculated by back-transformation of the 95% confidence limits estimated by using the Student-*t* test for differences (between Day 14 or 21 and Day 1) of antibodies (IgG and IgM) appearance with $\log_{10}$-transformation.

Geometric means confidence intervals were calculated by using the Student-*t* distribution in log-transformed data. Seroconversion confirmed by ELISA shall be defined as a 4-fold (or higher) increase in the antibodies titre over the baseline value.

**Neutralization Capability of Circulating Antibodies**

The ANOVA test shall be used to analyse the neutralization capability of circulating antibodies by estimating 95% CI.

**T-Cells Immunity Response**

T-Cell response shall be evaluated as ratio of subjects showing positive responses.

Fisher's exact test or $\chi^2$ test shall be used to analyse categorical values.

### 10.2.2 Analysis of Comparison between Antibodies Titers and the Administration Route (IN-IN, IM-IM, IN-IM)

A two-way ANOVA shall be used to compare differences between the administration route and concentration vs the antibodies-mediated immune response expressed.

### 10.2.3 Safety Data Analysis

**Adverse Events**

Percentages of adverse events occurred during the study shall be calculated. Adverse events should be documented with standardised terminology (MedDRA). Adverse events shall be considered under the following terms:

Adverse Events of Special Interest (AESIs), as defined in this protocol.
Adverse Events (AEs), as defined by the GCP – ICH/E6R2.
Serious Adverse Events (SAEs), as defined by the GCP – ICH/E6R2.

Adverse events shall be assessed as follows:

Analysis of adverse events of special interest (AESIs) shall consider number and percentage of subjects showing them after each vaccine administration.

Analysis of adverse events (AEs) shall consider number and percentage of subjects showing them after each vaccine administration.

Analysis of serious adverse events (SAEs) shall consider number and percentage of subjects showing them after each vaccine administration.

Description of adverse events should be as follows:

Adverse events of special interest shall be analysed within 7 days following administration of each vaccine.

Adverse events shall be analysed within 21 days following administration of the vaccine.

AESI, AE and SAE shall be registered according to the MedDRA terminology, i.e., severity (mild, moderated, or severe), start and end date, vaccine correlation and medical management.

AESIs shall be considered related with the vaccination.

Incidence rate of each adverse event shall be compared among groups by using the two-proportion z-test.

**Results of Clinical Laboratory and Physical Exam**

Results of clinically significant variations in the physical, clinical and laboratory examinations shall be summarised per vaccine concentration group and compared with each other.

Statistical analysis of adverse events, clinical laboratory variables and physical examination results shall be performed by comparing their pre- and post- values related to normal reference values.

A multivariate analysis shall be carried out to establish possible effects on immunogenicity, as well as the safety profile of candidate concentrations for the vaccine.

A two-tailed hypothesis test shall be performed with an alpha ($\alpha$) value of 0.05.

Safety endpoints shall be expressed as frequencies (%) with exact binomial 95% CIs; while immunological endpoints shall be expressed as medians and interquartile ranges (IQR), and these analyses shall be only for descriptive purposes.

### 10.2.4 Intermediate Analyses

Intermediate analyses shall be performed on Days 21, 28, 42, then by Month 6 and at the end of the study (Month 12).

Software SPSS shall be used for statistical analyses.

### 10.2.5 Information Processing

Software Statistical Package for Social Sciences (SPSS) Version 21 shall be used for statistical analysis.

## 11. Data Quality

Study Monitor shall ensure data collection is reliable by verifying data and matching Case Report Forms vs Clinical Record.

Database shall be prepared and subjected to procedures ensuring quality according to statistical procedures.

## 12. Protocol Approval Signatures

We have carefully read and reviewed this Clinical Study Protocol, and we perfectly understood its requirements and conditions. We agree that this Clinical Study Protocol fulfils the International requirements of Good Clinical Practices, as well as those stated by the Regulatory Authorities regarding corroboration of the source documents and audits/inspection of the study.

We agree that the materials used in this study, including the vaccines, are described in the protocol.

We understand that changes implemented to the protocol should be amendments approved by Dr Samuel Ponce de León Rosales in writing.

We agree the established period to carry out this protocol should be respected. This should start in ____________________________ and be completed in _____________________________.

DATE ________________________

| | |
|---|---|
| **Principal Investigator:** | **Signature** _________________________ |
| **Methodologist:** | **Signature** _________________________ |
| **Statistician:** | **Signature** _________________________ |
| **Responsible Person:** | **Signature** _________________________ |

# References

1. Consulted in 01-Mar-2021. Available https://www.worldometers.info/coronavirus/

2. Secretaría de Salud y Conacyt. https://datos.covid-19.conacyt.mx/ consulted in 2-Mar-2021.

3. OMS. (2020). Draft landscape of COVID-19 candidate vaccines. 3-Oct-2020, World Health Organization website: https://www.who.int/publications/m/item/draft-landscape-of-covid-19-candidate-vaccines

4. Khailany RA, Safdar M, Ozaslan M. Genomic characterization of a novel SARS-CoV-2. Gene Rep. 2020;19:100682. doi:10.1016/j.genrep.2020.100682.

5. Jiang Shibi et al., Emerging Microbes & Infections. 2012;1:1-8.

6. Vankadari N & Wilce JA. Emerging WuHan (COVID-19) coronavirus: glycan shield and structure prediction of *spike* glycoprotein and its interaction with human CD26. Emerging Microbes & Infections. 2020; 9:601-604.

7. Shang J et al., Nature. 2020; 581:221-224.

8. He YX et al., Biochem Biophys Res Commun. 2004; 324: 773-781.

9. Sohail A, Nutini A. Forecasting the timeframe of 2019-nCoV and human cells interaction with reverse engineering. Prog Biophys Mol Biol. 2020;S0079-6107(20)30026-2. doi:10.1016/j.pbiomolbio.2020.04.002.

10. Verdecchia P, Cavallini C, Spanevello A, Angeli F. The pivotal link between ACE2 deficiency and SARS-CoV-2 infection. Eur J Intern Med. 2020;S0953-6205(20) 30151-5. doi:10.1016/j.ejim.2020.04.037.

11. Magrone T, Magrone M, Jirillo E. Focus on Receptors for Coronaviruses with Special Reference to Angiotensin-converting Enzyme 2 as a Potential Drug Target - A Perspective. Endocr Metab Immune Disord Drug Targets. 2020;10.2174/1871530320666200427112.

12. Su S, Wong G, Shi W, Liu J, Lai ACK, Zhou J, Liu W, Bi Y, Gao GF. Epidemiology, Genetic Recombination, and Pathogenesis of Coronaviruses. Trends Microbiol. 2016;24:490-502.

13. Chen, N., Zhou, M., Dong, X., Qu, J., Gong, F., Han, Y., ... & Yu, T. (2020). Epidemiological and clinical characteristics of 99 cases of 2019 novel coronavirus pneumonia in Wuhan, China: a descriptive study. The Lancet, 395(10223), 507-513.

14. Felsenstein S, Herbert JA, McNamara PS, Hedrich CM. COVID-19: Immunology and treatment options. Clin Immunol. 2020;215:108448. doi:10.1016/j.clim.2020.108448.

15. Wichmann D, Sperhake JP, Lütgehetmann M, et al. Autopsy Findings and Venous Thromboembolism in Patients With COVID-19. Ann Intern Med. 2020;M20-2003. doi:10.7326/M20-2003.

16. Liu, Y., Gayle, A. A., Wilder-Smith, A., & Rocklöv, J. (2020). The reproductive number of COVID-19 is higher compared to SARS coronavirus. *Journal of travel medicine*.

17. Bindoli S, Felicetti M, Sfriso P, Doria A. The amount of cytokine-release defines different shades of Sars-Cov2 infection. Exp Biol Med (Maywood). 2020; doi:10.1177/1535370220928964.

18. Nile SH, Nile A, Qiu J, Li L, Jia X, Kai G. COVID-19: Pathogenesis, cytokine storm and therapeutic potential of interferons. Cytokine Growth Factor Rev. 2020;S1359-6101(20)30070-8. doi:10.1016/ j.cytogfr.2020.05.002.

19. Miller, P. J., Decanini, E. L., & Afonso, C. L. (2010). Newcastle disease: evolution of genotypes and the related diagnostic challenges. Infection, genetics and evolution, 10(1), 26-35.

20. Czegledi A, Ujvari D, Somogyi E, Wehmann E, Werner O, Lomniczi B. Third genome size category of avian paramyxovirus serotype 1 (Newcastle disease virus) and evolutionary implications. Virus Res. 2006; 120: 36-48.

21. Diel DG, da Silva LH, Liu H, Wang Z, Miller PJ, Afonso CL. Genetic diversity of avian paramyxovirus type 1: proposal for a unified nomenclature and classification system of Newcastle disease virus genotypes. Infect. Genet. Evol. 2012; 12:1770-1779.

22. Costa-Hurtado M, Afonso CL, Miller PJ, et al. Previous infection with virulent strains of Newcastle disease virus reduces highly pathogenic avian influenza virus replication, disease, and mortality in chickens. Vet Res. 2015; 46:97. doi:10.1186/s13567-015-0237-5.

23. Bai FL, Yu YH, Tian H, Ren GP, Wang H, Zhou B, Han XH, Yu QZ, Li DS. Genetically engineered Newcastle disease virus expressing interleukin-2 and TNF-related apoptosis-inducing ligand for cancer therapy. Cancer Biol Ther. 2014: 15:1226-1238.

24. Kapczynski DR, Afonso CL, Miller PJ. Immune responses of poultry to Newcastle disease virus. Dev Comp Immunol. 2013;41: 447-453.

25. Vigil A., Park MS, Martinez O, Chua MA, Xiao S, Cros JF, Martinez-Sobrido L, Woo SL, Garcia-Sastre Al. Use of reverse genetics to enhance the oncolytic properties of Newcastle disease virus. Cancer Res. 2007; 67:8285-8292.

26. Fournier P, Bian H, Szeberényi J, Schirrmacher V. Analysis of three properties of Newcastle disease virus for fighting cancer: tumour-selective replication, antitumor cytotoxicity, and immunostimulation Methods Mol Biol. 2012; 797:177-204.

27. Cheng X, Wang W, Xu Q, et al. Genetic Modification of Oncolytic Newcastle Disease Virus for Cancer Therapy. J Virol. 2016; 90: 5343-5352.

28. Cuadrado-Castano S, Ayllon J, Mansour M, de la Iglesia-Vicente J, Jordan S, Tripathi S, García-Sastre A, Villar E. Enhancement of the proapoptotic properties of Newcastle disease virus promotes tumour remission in syngeneic murine cancer models. Mol Cancer Ther. 2015; 14:1247-1258.

29. Schirrmacher V, Schlude C, Weitz J, Beckhove P. Strong T cell co-stimulation can reactivate tumour antigen specific T cells in late stage metastasized colorectal carcinoma patients: results from a phase I clinical study. Int J Oncol. 2015; 46:71–77.

30. Zhao H, Janke M, Fournier P, Schirrmacher V. Recombinant Newcastle disease virus expressing human interleukin-2 serves as potential candidate for tumour therapy. Virus Res. 2008; 136: 75-80.

31. Mohamed Amin Z, Che Ani MA, Tan SW et al. Evaluation of a Recombinant Newcastle Disease Virus Expressing Human IL12 against Human Breast Cancer. Sci Rep. 2019; 9:13999.

32. Kim SH & Samal SK. Newcastle disease virus as a vaccine vector for development of human and veterinary vaccines. Viruses 2016; 8: 183. Doi:10.3390/v8070183.

33. Bukreyev A & Collins PL. Newcastle disease virus as a vaccine vector for humans. Curr.Opin.Mol.Ther. 2008; 10: 46-55.

34. Huang Z., Krisnamurthy S., Panda A., Samal S.K. High-level expression of a foreign gene from the 3′ proximal first locus of a recombinant Newcastle disease virus. J. Gen. Virol. 2001; 82:1729–1736.

35. Carnero E., Li W., Borderia A.V., Moltedo B., Moran T., García-Sastre A. Optimization of human immunodeficiency virus Gag expression by Newcastle disease virus vectors for the induction of potent immune responses. J. Virol. 2009;83(2):584–597.

36. Zhao H., Peeters B.P.H. Recombinant Newcastle disease virus as a viral vector: Effect of genomic location of foreign gene on gene expression and virus replication. J. Gen. Virol. 2013;84:781–788.

37. Kim SH & Samal SK. Newcastle disease virus as a vaccine vector for development of human and veterinary vaccines. Viruses 2016; 8: 183. Doi:10.3390/v8070183.

38. Czegledi A, Ujvari D, Somogyi E, Wehmann E, Werner O, Lomniczi B. Third genome size category of avian paramyxovirus serotype 1 (Newcastle disease virus) and evolutionary implications. Virus Res. 2006; 120: 36-48.

39. Cantin, C., Holguera, J., Ferreira, L., Villar, E., & Munoz-Barroso, I. (2007). Newcastle disease virus may enter cells by caveolae-mediated endocytosis. Journal of general virology, 88(2), 559-569.

40. Panda, A., Huang, Z., Elankumaran, S., Rockemann, D. D., & Samal, S. K. (2004). Role of fusion protein cleavage site in the virulence of Newcastle disease virus. Microbial pathogenesis, 36(1), 1-10

41. Peeters, B. P., de Leeuw, O. S., Koch, G., & Gielkens, A. L. (1999). Rescue of Newcastle disease virus from cloned cDNA: evidence that cleavability of the fusion protein is a major determinant for virulence. Journal of virology, 73(6), 5001-5009.

42. Leeuw, O. S., Koch, G., Hartog, L., Ravenshorst, N., & Peeters, B. P. (2005). Virulence of Newcastle disease virus is determined by the cleavage site of the fusion protein and by both the stem region and globular head of the haemagglutinin–neuraminidase protein. Journal of General Virology, 86(6), 1759-1769.

43. Engel-Herbert, I., Werner, O., Teifke, J. P., Mebatsion, T., Mettenleiter, T. C., & Römer-Oberdörfer, A. (2003). Characterization of a recombinant Newcastle disease virus expressing the green fluorescent protein. Journal of virological methods, 108(1), 19-28.

44. Cornax, I., Diel, D. G., Rue, C. A., Estevez, C., Yu, Q., Miller, P. J., & Afonso, C. L. (2013). Newcastle disease virus fusion and haemagglutinin-neuraminidase proteins contribute to its macrophage host range. The Journal of General Virology, 94(Pt 6), 1189.

45. Huang, Z., Panda, A., Elankumaran, S., Govindarajan, D., Rockemann, D. D., & Samal, S. K. (2004). The hemagglutinin-neuraminidase protein of Newcastle disease virus determines tropism and virulence. Journal of virology, 78(8), 4176-4184.

46. Nakaya, T., Cros, J., Park, M. S., Nakaya, Y., Zheng, H., Sagrera, A., ... & Palese, P. (2001). Recombinant Newcastle disease virus as a vaccine vector. Journal of virology, 75(23), 11868-11873.

47. Honda, K., Sakaguchi, S., Nakajima, C., Watanabe, A., Yanai, H., Matsumoto, M., ... & Seya, T. (2003). Selective contribution of IFN-α/β signalling to the maturation of dendritic cells induced by double-stranded RNA or viral infection. Proceedings of the National Academy of Sciences, 100(19), 10872-10877.

48. Buijs, P. R., van Amerongen, G., van Nieuwkoop, S., Bestebroer, T. M., Van Run, P. R. W. A., Kuiken, T., ... & Van Den Hoogen, B. G. (2014). Intravenously injected Newcastle disease virus in non-human primates is safe to use for oncolytic virotherapy. Cancer gene therapy, 21(11), 463-471.

49. Bukreyev, A., Huang, Z., Yang, L., Elankumaran, S., Claire, M. S., Murphy, B. R., ... & Collins, P. L. (2005). Recombinant Newcastle disease virus expressing a foreign viral antigen is attenuated and highly immunogenic in primates. Journal of Virology, 79(21), 13275-13284.

50. DiNapoli, J. M., Kotelkin, A., Yang, L., Elankumaran, S., Murphy, B. R., Samal, S. K., ... & Bukreyev, A. (2007). Newcastle disease virus, a host range-restricted virus, as a vaccine vector for intranasal immunization against emerging pathogens. Proceedings of the National Academy of Sciences, 104(23), 9788-9793

51. DiNapoli, J. M., Yang, L., Suguitan, A., Elankumaran, S., Dorward, D. W., Murphy, B. R., ... & Bukreyev, A. (2007). Immunization of primates with a Newcastle disease virus-vectored vaccine via the respiratory tract induces a high titer of serum neutralizing antibodies against highly pathogenic avian influenza virus. Journal of Virology, 81(21), 11560-11568.

52. DiNapoli JM, Ward JM, Cheng L, et al. Delivery to the lower respiratory tract is required for effective immunization with Newcastle disease virus-vectored vaccines intended for humans. Vaccine 2009; 27: 1530–1539.

53. Viktorova, E. G., Khattar, S. K., Kouiavskaia, D., Laassri, M., Zagorodnyaya, T., Dragunsky, E., ... & Belov, G. A. (2018). Newcastle disease virus-based vectored vaccine against poliomyelitis. Journal of virology, 92(17).

54. Martinez-Sobrido, L., Gitiban, N., Fernandez-Sesma, A., Cros, J., Mertz, S. E., Jewell, N. A., ... & Durbin, J. E. (2006). Protection against respiratory syncytial virus by a recombinant Newcastle disease virus vector. Journal of Virology, 80(3), 1130-1139.

55. SUN, Weina, et al. Newcastle disease virus (NDV) expressing the *spike* protein of SARS-CoV-2 as vaccine candidate. bioRxiv preprint doi: https://doi.org/10.1101/2020.07.26.221861. This version posted July 26, 2020.

56. SUN, Weina, et al. A Newcastle disease virus (NDV) expressing membrane-anchored *spike* as a cost-effective inactivated SARS-CoV-2 vaccine. bioRxiv preprint doi: https://doi.org/10.1101/2020.07.30.229120. This version posted July 31, 2020.

57. Lurie N, Saville M, Hatchett R, Halton J. Developing Covid-19 vaccines at pandemic speed. N Engl J Med 2020; 382:1969-1973.

58. W. Sun, S. R. Leist, S. McCroskery, Y. Liu, S. Slamanig, J. Oliva, F. Amanat, A. Schäfer, K. H. D. III, A. García-Sastre, F. Krammer, R. S. Baric y P. Palese, «Newcastle disease virus (NDV) expressing the *spike* protein of SARS-CoV-2 as vaccine candidate.» *bioRxiv: the preprint server for biology,* vol. doi: https://doi.org/10.1101/2020.07.26.221861, 2020.

# Part II. Ethical and Administrative Considerations

## Confidentiality Statement

The information contained herein is confidential and property of Laboratorio Avi-Mex S.A. de C.V. (Avimex*). We trust you, your personnel, the Research Ethics Committees, the Institutional Review Board, and the Regulatory Authorities to review it. It is understood that it cannot be provided to others without prior written authorization of Laboratorio Avi-Mex S.A de C.V (Avimex*), except when it is necessary to provide this information to obtain the Informed Consent of the candidates to vaccine administration.

## 1. Ethical considerations

### 1.1. Declaration of Helsinki

The Investigator guarantees this study shall be carried out by following strictly the "Declaration of Helsinki" principles (as amended in 2013, Fortaleza, Brazil), as well as the legislation and regulation of the country in which the investigation is carried out, which provides subjects with utmost protection.

The Declaration of Helsinki is accepted as ethical basis in Clinical Studies to ensure complete follow-up and respect of investigation in human beings. Some exceptions should be well justified in the Protocol. It is guaranteed that subjects are protected by the Research Ethics Committees and Informed Consent.

### 1.2. Good Clinical Practices

Good Clinical Practices serve as standard for Clinical Studies, in which the design, conduction, monitoring, finishing, audit, analysis, report and documentation of studies is ethically and scientifically justified and clinical properties of diagnosis, therapeutic or prophylactic products under investigation are appropriately documented.

The study should strictly follow principles of the ICH harmonised tripartite guideline: Guides of Good Clinical Practices (January 1997 and November 2016) or local legislation if this provides the subject with better protection.

# 2. Investigator Responsibilities

The Investigator is responsible for conducting the study as stated in the protocol and ensuring its availability for patients involved in the study within a period defined in that mentioned protocol.

## 2.1 Ethics

### 2.1.1 Declaration of Helsinki

The Investigator is responsible for ensuring the study is conducted in total compliance with the principles of the **Declaration of Helsinki**, amended version (2013). Annex 3.

### 2.1.3 Good Clinical Practices

The Investigator is responsible for ensuring the study is conducted according to the International Council for Harmonisation **Good Clinical Practices – ICH/GCP E6R2** and according to the local regulatory laws applicable to clinical studies.

### 2.1.4 Research Ethics Committees

The Investigator is responsible for submitting a protocol copy and information detailed (which will be presented to the patient in the Informed Consent) before the Research Ethics Committee, which in turn should approve the protocol prior its execution and provide the Investigator with the approval letter, who will send a copy to the Sponsor(s). The approval letter from the Research Ethics Committees should reference its members and designation.

### 2.1.5 Informed Consent

The Investigator (or designee, if acceptable by local legislation) is responsible for preparing and checking objectives, methods, potential risk, rights, and obligations that comprise the Informed Consent of every subject involved in the study. The Consent should be obtained upon explained all study procedures in detail and responded all questions asked by the investigation subjects. If the subject accepts to participate in the study, his/her acceptance shall be documented in the Informed Consent format.

The Investigator shall also explain the subjects that they can decline or leave the study any time and due to any reason. The Investigator or designee shall provide formats intended to document this informed consent.

For under-age subjects who cannot provide their Informed Consent, this should be obtained from an acceptable legal representative. Should the subject and its Legal Representative cannot read, two neutral witnesses should attend the discussion related to the informed consent. Once the subject and the legal representative have given oral consent to participate in the study, signature from both witnesses shall certify the information contained in the consent was perfectly explained and understood.

Case Report Forms of this study contain a section exclusive to document the informed consent, which should be filled properly. In case of collecting new data on safety which significantly modify risks and benefits evaluation, the Informed Consent should be revised and updated, if necessary. All subjects (even those in treatment) should be informed receiving a copy of the new version and give their consent to continue participating in the study.

### 2.1.6 Subject withdrawal

The Investigator has authority to **withdraw a subject** due to several reasons, which include optimal benefit of the subject, intercurrent diseases, adverse events, or treatment failures. When a subject wants to leave the study due to any reason, he/she should complete the final evaluation and declare his/her withdrawal reason. In case of follow-up lost the reason shall be stated as non-

attendance. Withdrawal due to intercurrent disease and adverse events should be extensively documented in Case Report Forms with available and appropriate supplementary information.

#### 2.1.7 Subjects Privacy

The Investigator should ensure privacy of subjects. All documents subjected to Laboratorio Avi-Mex S.A. de C.V. should identify subjects by identification code, not by names or hospital/clinical number. The Investigator should keep confidential all data that identify codes with names and addresses of the subjects involved in the study. Documents not subjected to Laboratorio Avi-Mex S.A. de C.V. should be safeguarded by the Investigator under strict confidentiality.

## 3. Conditions of Protocol Amendment

An ongoing study protocol can be modified only by Avi-Mex S.A. de C.V. approval, and this shall be submitted before the Research Ethics Committee, as well as the corresponding authority as amendment.

### 3.1 Protocol Amendments

All amendments agreed to the protocol should be approved in writing by the Investigator; amendments should be submitted before the Research Ethics Committee and regulatory agencies, who should review and authorize all protocol amendments in conformity with local legislation. Without authorization modifications cannot be implemented unless these are necessary to remove an immediate risk to study subjects or logistical or administrative modifications.

### 3.2 Case Report Forms

The Investigator is responsible for ensuring the electronic Case Report Forms are filled out accurately. There should be a case report form for each subject involved in the study. The Case Report Forms should be filled out properly according to the instructions; the electronic Case Report Forms should have the traceability requested by the authority, as well as an electronic signature.

## 3.3 Source Document Verification

In accordance with the standards of data protection laws, all data obtained during clinical study should be treated with discretion and in order guaranteeing the right to privacy of patients.

The Investigator should permit the monitor/auditor/inspector have access to all study materials necessary to verify the data source and establish an adequate review of the study progress.

# 4. Protocol Suspension Conditions

The Investigator reserves the right to discontinue the study at any time. If necessary, suspension procedures will be established on a case-by-case basis after a case study. When suspending the study, the Investigator will guarantee a correct protection of patients interests.

# 5. Adverse Events

The Investigator is responsible for reporting all adverse events in Case Report Forms. Serious adverse events (SAEs) have to be reported to the CRO, the Committees and Laboratorio Avi-Mex S.A. de C.V. immediately on a business day.

## 5.1 Definition

Any new medical event that occurs in a patient or clinical investigation subject who has been administered with the study drug and not necessarily have a causal relationship with the treatment.

Therefore, adverse events may be unfavourable and unexpected signs (including laboratory abnormalities), symptoms or temporary illness associated with the use of the investigational drug, whether related to the study drug.

Pre-existing conditions which worsen during study are reported as adverse events.

These may become Serious Adverse Events if fulfil any of these criteria:

- Death (***note:*** death is a result, not an event).
- Life-threatening (***note:*** it refers an event when patient is at risk of dying because of the event, not a hypothetical cause of death if the event were more severe).
- Patients requiring hospitalization or its prolongation (***note:*** hospitalization unexpected).
- Persistence of results, disability/permanent damage.
- Congenital anomalies, birth defects.
- Clinically significant or requires intervention to prevent any results abovementioned.

All these adverse events are considered SERIOUS and the Investigator should report them immediately on business day to Laboratorio Avi-Mex S.A. de C.V. It is a legal requirement to report all SERIOUS adverse events. All serious adverse events should also be reported in the respective section of adverse events in Case Report Forms. The International Ethical Guides require to inform all serious adverse events to the Research Ethics Committee.

## 5.2 Unexpected Adverse Events

An unexpected adverse event that has not been reported in product information.

## 5.3 Causality

Causality involves any of these three possibilities:

**“NO”** (defined as not related to the study drug).

**YES** (remotely, possibly, probably, or definitely related to the drug).

**UNKONWN**

All adverse events qualified by the Investigator or Laboratorio Avi-Mex S.A. de C.V. as "DEFINITELY RELATED TO THE VACCINE" qualify as Adverse Event of Interest.

## 5.4 Severity of Adverse Event – Definition

Severity of an adverse event can be categorised with a three-point or four-point scale (*WHO – Handbook for Reporting Results of Cancer Treatment).*

### 5.4.1 Three-Point Scale – Definition

**Low or *Grade 1:*** evident malaise, but it does not interrupt a normal life activities.

**Moderate or *Grade 2:*** sufficient malaise to reduce or affect normal life activities.

**Severe or *Grade 3:*** incapacity to perform work or normal life activities.

## 5.5 Treatment and Follow-up of adverse events

All adverse events should be documented and followed-up until resolution or adequately explained, even when the patient is no longer involved the clinical study.

# 6. Publication of Results and Protection of Trade Secrets

The Investigator and Laboratorio Avi-Mex S.A de C.V are responsible for publishing the results of the clinical study as soon as it has been completed. The Investigator agrees to inform the Laboratory Sponsor to review all original manuscripts or abstracts before publishing them. This will allow Laboratorio Avi-Mex S.A de C.V. protect your copyrights and make comments based on information that the Investigator(s) may not know.

According to conventional editorial and ethical practice, Laboratorio Avi-Mex S.A de C.V. will support publication of the study results; if more than two centres are involved, it is then a global publication. In this case, a Coordinating Investigator will be appointed by mutual agreement.

The list of authors will be determined by common agreement with the participating investigators.

# 7. Study Documentation, Case Report Form and Other Registries

## 7.1 Special Considerations

## 7.2 Investigator Files/Documents Preservation

The Investigator must keep adequate and accurate records that perfectly document study development and that allow subsequent verification of study data. These documents can be classified into two separate categories: (1) the Investigator Site File, and (2) subject's original clinical documents.

The Investigator Site File will contain protocol with its amendments, Research Ethics Committee, Research Committee and Biosafety Committee authorizations and those from governmental authorities along with the correspondence managed, a copy of the informed consent, the records of drugs provided and returned, resumes of personnel involved, authorization forms, and other applicable documents.

The subjects' original clinical documents (which are usually defined in advance by the project management to record efficacy and safety parameters in CRFs) include hospital or clinical records, doctor's and nurse's notes, appointments, original laboratory reports, results of ECGs, X-rays, CT scans, pathology reports or other special reports, signed informed consent forms, letters from consultants, and subject selection and inclusion records. The Investigator must keep these two categories of documents in their files for a minimum period of 15 years from the conclusion or suspension of the study. At the end of this period, the documents can be destroyed according to local regulations.

If the Investigator wants to transfer the study records to a third party or move them to another place, he/she should notify Laboratorio Avi-Mex S.A de C.V. in advance.

If the Investigator cannot guarantee these conditions at the investigation site for some or all documents, special arrangements shall be made between the Investigator and Laboratorio Avi-Mex S.A de C.V. to keep them in a closed container off-site, so these can be returned unopened to the Investigator in case of official inspection. If original documents are needed throughout the patient's treatment period, copies will be made as necessary and kept off-site.

## 7.3. Original documents and basic data

The Investigator will safeguard the original data that the Research Ethics Committee and the corresponding authorities may request, both the documentation and the clinical records of the study. This is particularly important when electronic Case Record Forms are confusing or when data transcription errors are suspected. In case of special problems, official requests, or inspections, access to all study records is also necessary, always keeping confidentiality.

## 7.4. Audits and inspections

The Investigator should understand that the original documents of this study should be made available to the qualified personnel of the Research Ethics Committee and inspectors from health authorities, prior notification. Data contained in Electronic Case Report Forms should be verified by direct inspection of the original documents.

## 7.5 Study Supervision

It is understood that between the monitor responsible for Laboratorio Avi-Mex S.A. of C.V. (or designee) and the Investigator contact is constant visits shall be regular. Upon request, you will be allowed to inspect the various study records (clinical records, electronic Case Report Forms, and other relevant data), always keeping confidentiality according to local laws.

The monitor will be responsible for regularly reviewing electronic Case Report Forms, verifying compliance with the protocol, consistency and accuracy of the data recorded, and verifying data are complete. The monitor will have access to laboratory test reports and other patient records to verify data from case reports. The Investigator (or designee) agrees to cooperate with the monitor to resolve any issues that may be found during these visits.

A frequency of at least one monitoring visit is anticipated at the end of key visits for all subjects, Baseline, Day 21, Day 28, Day 42, Month 3, Month 6, and Month 12 to the investigation site.

# 8. Responsibilities of the Laboratory Providing the Investigational Product

## 8.1. General Responsibilities.

It is the responsibility of Laboratorio Avi-Mex S.A de C.V. updating Product Background (**Investigator's Manual**) every time new information appears on a product not yet authorized for marketing. If the product is already approved for marketing, updated information will be inserted on product's packaging and its monograph.

# 9. Responsibilities of the Monitor

The monitor is responsible for becoming familiar with the Investigator, with all centre staff involved in the study, and with all study procedures, including administration of the study vaccine.

Laboratory Avi-Mex S.A de C.V. will train the monitor who assists the Investigator in conducting the clinical study. The monitor has to visit the clinical study centre before the first patient is enrolled to establish the necessary bases for good development of the study, visit the centre during and at the end of the study until it has been completely finished.

The monitor is responsible for reviewing the study follow-up along with the Investigator, verifying adherence to the protocol and transferring any problems that may arise. At the same time Laboratorio Avi-Mex S.A de C.V. has to keep the study documents confidential.

The monitor is responsible for verifying that data established in the study documents (Case Report Forms) are documented in clinical records (notes, original laboratory results, etc.), which are considered source documents. There cannot be data in the Case Report Forms not documented in writing or by any means, in the source documents.

# ANNEX 1

# CATEGORIES TO CLASSIFY ADVERSE EVENTS INTENSITY AND THEIR ASSOCIATION WITH THE EXPERIMENTAL INTERVENTION

Severity of adverse events shall be classified with a three-point scale (low, moderate and severe) and described in detail along with the Investigator evaluation on adverse event association with the study vaccine.

Severity of an adverse event can be categorised with a three-point scale (*WHO – Handbook for Reporting Results of Cancer Treatment)*.

**Categories to determine adverse events' severity:**

**Three-Point Scale – Definition**

**Low or *Grade 1:*** evident malaise, but it does not interrupt a normal life activities.

**Moderate or *Grade 2:*** sufficient malaise to reduce or affect normal life activities.

**Severe or *Grade 3:*** incapacity to perform work or normal life activities.

**Categories to determine the adverse events association with the experimental intervention**

**Probable**

This category applies to adverse events considered with a high degree of certainty to be related to experimental intervention. An adverse event can be considered probable if it includes at least the first three points of the following list:

- It has a reasonable time sequence for administration of the experimental intervention.

- It cannot be reasonably explained by known characteristics of patient's clinical condition, environmental or toxic factors, or other treatment administered to the patient.
- It disappears or decreases when stopping or reducing the dose.
- It follows a known pattern of response to the suspected experimental intervention.
- It reappears with re-exposure to intervention.

**Possible**

This category applies to those adverse effects where a relationship with the intervention does not seem likely but cannot be ruled out with certainty. An adverse event can be considered possible if it includes at least the first two points:

- It follows a reasonable time sequence from the administration of the vaccine.
- It could have been caused by patient's clinical condition, environmental or toxic factors, or other treatments administered to the patient.
- It follows a known response pattern to the experimental intervention.

**Remote**

In general, this category is applied to an adverse event that meets at least two of the following points:

- It does not follow a reasonable time sequence from the administration of the vaccine.
- It could easily have been caused by patient's clinical condition, some environmental or toxic factor, or other treatment administered to the patient.
- It does not follow a known response pattern to the experimental intervention.
- It does not recur or worsen when administering the experimental intervention.

**Not related**

This category is applicable to those adverse events that are clearly and incontrovertibly considered to be due to external causes solely and do not meet the criteria of relationship to the experimental intervention abovementioned for “remote”, “possible” or “probable” categories.

**Categories to determine causality**

Causality can have any of these three possibilities:

- **"NO"** (defined as not related to study drug).
- **"YES"** (remotely, possibly, probably, or definitely related to the drug).
- **UNKNOWN**

All adverse events qualified by the Investigator or Sponsor as *"definitely related to the vaccine"* qualify as adverse reactions to the vaccine.

It is not necessary to fill in the adverse events page on the Case Report Forms for pre-existing adverse medical conditions during the screening period that do not worsen in severity nor frequency during the study.

Adverse conditions should be adequately documented in patient's clinical records and in Case Report Forms. Adverse medical conditions existent at screening but worsen after exposure to the study intervention should be reported as adverse events.

# ANNEX 2

## SERIOUS ADVERSE EVENT DEFINITION

For purposes of this study an adverse event is considered “serious” when it fulfils any of these criteria:

- Death (***note:*** death is a result, not an event).
- Life-threatening (***note:*** it refers an event when patient is at risk of dying because of the event, not a hypothetical cause of death if the event were more severe).
- Patients requiring hospitalization or its prolongation (***note:*** hospitalization unexpected).
- Persistence of results, disability/permanent damage.
- Congenital anomalies, birth defects.
- Clinically significant or requires intervention to prevent any results abovementioned.

All these adverse events are considered SERIOUS and the Investigator should report them immediately on business day to Laboratorio Avi-Mex S.A. de C.V. It is a legal requirement to report all SERIOUS adverse events. All serious adverse events should also be reported in the respective section of adverse events in Case Report Forms. The International Ethical Guides require to inform all serious adverse events to the Research Ethics Committee and corresponding authorities.

# ANNEX 3

# WORLD MEDICAL ASSOCIATION
# DECLARATION OF HELSINKI

## WMA Declaration of Helsinki –
## Ethical principles for medical research involving human beings

Adopted by the 18th WMA General Assembly, Helsinki, Finland, June 1964
and amended by the:
29th WMA General Assembly, Tokyo, Japan, October 1975
35th WMA General Assembly, Venice, Italy, October 1983
41st WMA General Assembly, Hong Kong, September 1989
48th WMA General Assembly, Somerset West, Republic of South Africa, October 1996
52nd WMA General Assembly, Edinburgh, Scotland, October 2000
Note of Clarification added by the WMA General Assembly, Washington 2002
Note of Clarification added by the WMA General Assembly, Tokyo 2004
59th WMA General Assembly, Seoul, Republic of Korea, October 2008
64th WMA General Assembly, Fortaleza, Brazil, October 2013

**Preamble**

1. The World Medical Association (WMA) has developed the Declaration of Helsinki as a statement of ethical principles for medical research involving human subjects, including research on identifiable human material and data.
   The Declaration is intended to be read as a whole and each of its constituent paragraphs should be applied with consideration of all other relevant paragraphs.
2. Consistent with the mandate of the WMA, the Declaration is addressed primarily to physicians. The WMA encourages others who are involved in medical research involving human subjects to adopt these principles.

**General Principles**

3. The Declaration of Geneva of the WMA binds the physician with the words, "The health of my patient will be my first consideration," and the International Code of Medical Ethics declares that "A physician shall act in the patient's best interest when providing medical care."
4. It is the duty of the physician to promote and safeguard the health, well-being, and rights of patients, including those who are involved in medical research. The physician's knowledge and conscience are dedicated to the fulfilment of this duty.

5. Medical progress is based on research that ultimately must include studies involving human subjects.
6. The primary purpose of medical research involving human subjects is to understand the causes, development and effects of diseases and improve preventive, diagnostic and therapeutic interventions (methods, procedures, and treatments). Even the best proven interventions must be evaluated continually through research for their safety, effectiveness, efficiency, accessibility, and quality.

7. Medical research is subject to ethical standards that promote and ensure respect for all human subjects and protect their health and rights.
8. While the primary purpose of medical research is to generate new knowledge, this goal can never take precedence over the rights and interests of individual research subjects.
9. It is the duty of physicians who are involved in medical research to protect the life, health, dignity, integrity, right to self-determination, privacy, and confidentiality of personal information of research subjects. The responsibility for the protection of research subjects must always rest with the physician or other health care professionals and never with the research subjects, even though they have given consent.
10. Physicians must consider the ethical, legal, and regulatory norms and standards for research involving human subjects in their own countries as well as applicable international norms and standards. No national or international ethical, legal, or regulatory requirement should reduce or eliminate any of the protections for research subjects set forth in this Declaration.
11. Medical research should be conducted in a manner that minimises possible harm to the environment.
12. Medical research involving human subjects must be conducted only by individuals with the appropriate ethics and scientific education, training, and qualifications. Research on patients or healthy volunteers requires the supervision of a competent and appropriately qualified physician or other health care professional.
13. Groups that are underrepresented in medical research should be provided appropriate access to participation in research.
14. Physicians who combine medical research with medical care should involve their patients in research only to the extent that this is justified by its potential preventive, diagnostic or therapeutic value and if the physician has good reason to believe that participation in the research study will not adversely affect the health of the patients who serve as research subjects.

15. Appropriate compensation and treatment for subjects who are harmed as a result of participating in research must be ensured.

**Risks, Burdens and Benefits**

16. In medical practice and in medical research, most interventions involve risks and burdens.
    Medical research involving human subjects may only be conducted if the importance of the objective outweighs the risks and burdens to the research subjects.
17. All medical research involving human subjects must be preceded by careful assessment of predictable risks and burdens to the individuals and groups involved in the research in comparison with foreseeable benefits to them and to other individuals or groups affected by the condition under investigation.
    Measures to minimise the risks must be implemented. The risks must be continuously monitored, assessed, and documented by the researcher.
18. Physicians may not be involved in a research study involving human subjects unless they are confident that the risks have been adequately assessed and can be satisfactorily managed.
    When the risks are found to outweigh the potential benefits or when there is conclusive proof of definitive outcomes, physicians must assess whether to continue, modify or immediately stop the study.

**Vulnerable Groups and Individuals**

19. Some groups and individuals are particularly vulnerable and may have an increased likelihood of being wronged or of incurring additional harm.
    All vulnerable groups and individuals should receive specifically considered protection.
20. Medical research with a vulnerable group is only justified if the research is responsive to the health needs or priorities of this group and the research cannot be carried out in a non-vulnerable group. In addition, this group should stand to benefit from the knowledge, practices or interventions that result from the research.

**Scientific Requirements and Research Protocols**

21. Medical research involving human subjects must conform to generally accepted scientific principles, be based on a thorough knowledge of the scientific literature, other relevant sources of information, and adequate laboratory and, as appropriate, animal experimentation.
    The welfare of animals used for research must be respected.

22. The design and performance of each research study involving human subjects must be clearly described and justified in a research protocol.
    The protocol should contain a statement of the ethical considerations involved and should indicate how the principles in this Declaration have been addressed. The protocol should include information regarding funding, sponsors, institutional affiliations, potential conflicts of interest, incentives for subjects and information regarding provisions for treating and/or compensating subjects who are harmed because of participation in the research study.
    In clinical trials, the protocol must also describe appropriate arrangements for post-trial provisions.

**Research Ethics Committees**

23. The research protocol must be submitted for consideration, comment, guidance, and approval to the concerned research ethics committee before the study begins. This committee must be transparent in its functioning, must be independent of the researcher, the sponsor and any other undue influence and must be duly qualified. It must take into consideration the laws and regulations of the country or countries in which the research is to be performed as well as applicable international norms and standards, but these must not be allowed to reduce or eliminate any of the protections for research subjects set forth in this Declaration.
    The committee must have the right to monitor ongoing studies. The researcher must provide monitoring information to the committee, especially information about any serious adverse events. No amendment to the protocol may be made without consideration and approval by the committee. After the end of the study, the researchers must submit a final report to the committee containing a summary of the study's findings and conclusions.

**Privacy and Confidentiality**

24. Every precaution must be taken to protect the privacy of research subjects and the confidentiality of their personal information.

**Informed Consent**

25. Participation by individuals capable of giving informed consent as subjects in medical research must be voluntary. Although it may be appropriate to consult family members or community leaders, no individual capable of giving informed consent may be enrolled in a research study unless he or she freely agrees.

26. In medical research involving human subjects capable of giving informed consent, each potential subject must be adequately informed of the aims, methods, sources of funding, any possible conflicts of interest, institutional affiliations of the researcher, the anticipated benefits and potential risks of the study and the discomfort it may entail, post-study provisions and any other relevant aspects of the study. The potential subject must be informed of the right to refuse to participate in the study or to withdraw consent to participate at any time without reprisal. Special attention should be given to the specific information needs of individual potential subjects as well as to the methods used to deliver the information.
    After ensuring that the potential subject has understood the information, the physician or another appropriately qualified individual must then seek the potential subject's freely given informed consent, preferably in writing. If the consent cannot be expressed in writing, the non-written consent must be formally documented and witnessed.
    All medical research subjects should be given the option of being informed about the general outcome and results of the study.
27. When seeking informed consent for participation in a research study the physician must be particularly cautious if the potential subject is in a dependent relationship with the physician or may consent under duress. In such situations the informed consent must be sought by an appropriately qualified individual who is completely independent of this relationship.
28. For a potential research subject who is incapable of giving informed consent, the physician must seek informed consent from the legally authorised representative. These individuals must not be included in a research study that has no likelihood of benefit for them unless it is intended to promote the health of the group represented by the potential subject, the research cannot instead be performed with persons capable of providing informed consent, and the research entails only minimal risk and minimal burden.
29. When a potential research subject who is deemed incapable of giving informed consent is able to give assent to decisions about participation in research, the physician must seek that assent in addition to the consent of the legally authorised representative. The potential subject's dissent should be respected.
30. Research involving subjects who are physically or mentally incapable of giving consent, for example, unconscious patients, may be done only if the physical or mental condition that prevents giving informed consent is a necessary characteristic of the research group. In such circumstances the physician must seek informed consent from the legally authorised representative. If no such representative is available and if the research cannot be delayed, the study may proceed without informed consent provided that the specific reasons for involving subjects with a condition that renders them unable to give informed consent have

been stated in the research protocol and the study has been approved by a research ethics committee. Consent to remain in the research must be obtained as soon as possible from the subject or a legally authorised representative.

31. The physician must fully inform the patient which aspects of their care are related to the research. The refusal of a patient to participate in a study or the patient's decision to withdraw from the study must never adversely affect the patient-physician relationship.
32. For medical research using identifiable human material or data, such as research on material or data contained in biobanks or similar repositories, physicians must seek informed consent for its collection, storage and/or reuse. There may be exceptional situations where consent would be impossible or impracticable to obtain for such research. In such situations the research may be done only after consideration and approval of a research ethics committee.

**Use of Placebo**

33. The benefits, risks, burdens, and effectiveness of a new intervention must be tested against those of the best proven intervention(s), except in the following circumstances:

    Where no proven intervention exists, the use of placebo, or no intervention, is acceptable; or where for compelling and scientifically sound methodological reasons the use of any intervention less effective than the best proven one, the use of placebo, or no intervention is necessary to determine the efficacy or safety of an intervention.

    Patients who receive any intervention less effective than the best proven one, placebo, or no intervention will not be subject to additional risks of serious or irreversible harm as a result of not receiving the best proven intervention.

    Extreme care must be taken to avoid abuse of this option.

**Post-Trial Provisions**

34. In advance of a clinical trial, sponsors, researchers, and host country governments should make provisions for post-trial access for all participants who still need an intervention identified as beneficial in the trial. This information must also be disclosed to participants during the informed consent process.

**Research Registration and Publication and Dissemination of Results**

35. Every research study involving human subjects must be registered in a publicly accessible database before recruitment of the first subject.
36. Researchers, authors, sponsors, editors, and publishers all have ethical obligations with regard to the publication and dissemination of the results of research. Researchers have a duty to

make publicly available the results of their research on human subjects and are accountable for the completeness and accuracy of their reports. All parties should adhere to accepted guidelines for ethical reporting. Negative and inconclusive as well as positive results must be published or otherwise made publicly available. Sources of funding, institutional affiliations and conflicts of interest must be declared in the publication. Reports of research not in accordance with the principles of this Declaration should not be accepted for publication.

**Unproven Interventions in Clinical Practice**

37. In the treatment of an individual patient, where proven interventions do not exist or other known interventions have been ineffective, the physician, after seeking expert advice, with informed consent from the patient or a legally authorised representative, may use an unproven intervention if in the physician's judgement it offers hope of saving life, re-establishing health, or alleviating suffering. This intervention should subsequently be made the object of research, designed to evaluate its safety and efficacy. In all cases, new information must be recorded and, where appropriate, made publicly available.

# ANNEX 4
# PATIENT DIARY

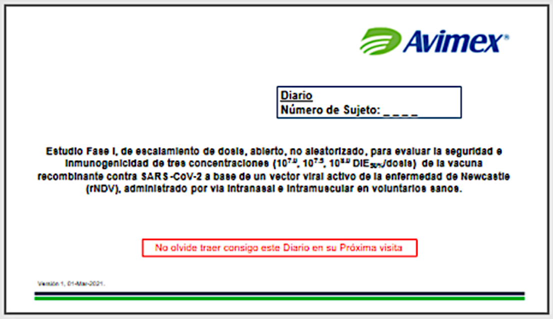


1

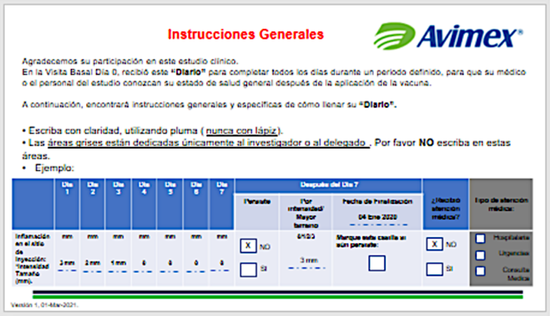


2

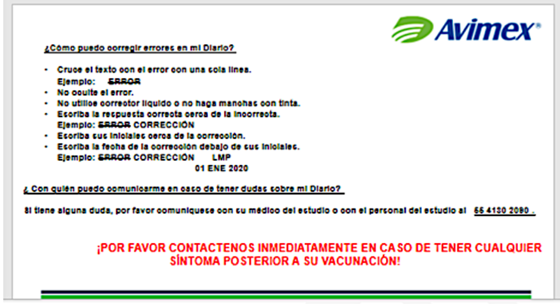


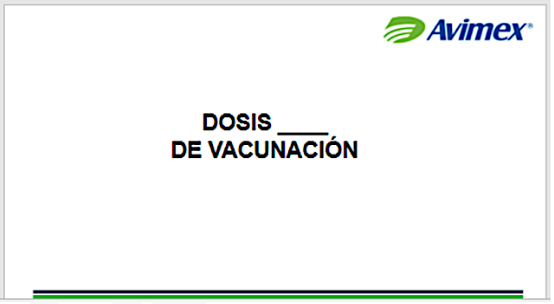


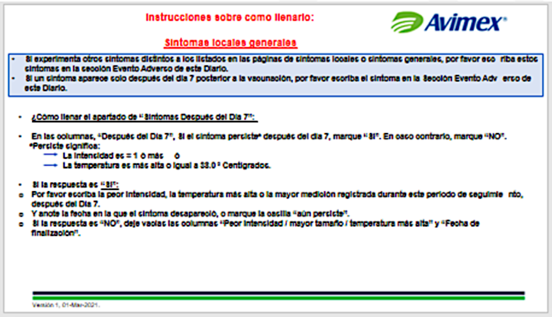


5

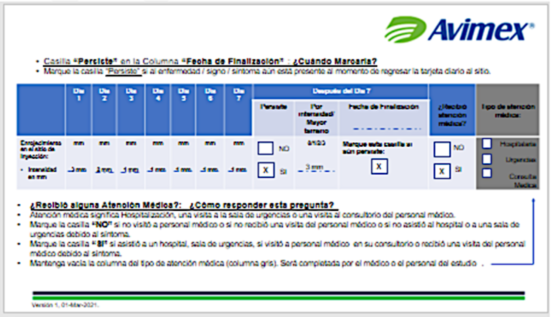


6

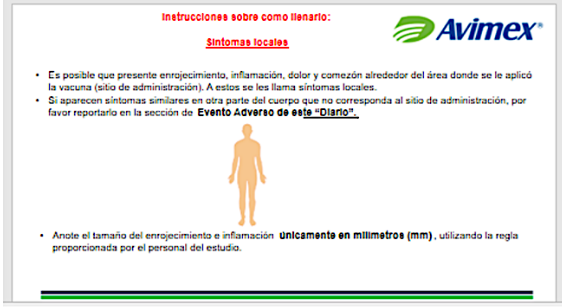


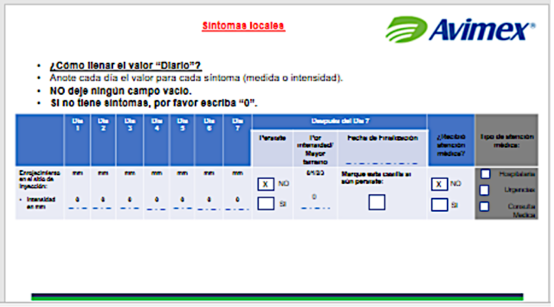

**Síntomas locales**

Avimex®

- Definición de **"Intensidad":**
- **Enrojecimiento e inflamación en el sitio de inyección:**
  Medir y registrar el mayor diámetro de superficie en milímetros (mm).

- **Dolor en el sitio de inyección:**
  **0: Ninguno.**
  **1: Leve**: Dolor que no interfiere ni impide el desarrollo de mis actividades cotidianas normales.
  **2: Moderado**: Es doloroso mover los brazos e interfiere con mis actividades cotidianas.
  **3: Grave**: Dolor significativo en estado de reposo. Impide desarrollo de mis actividades cotidianas normales.

- **Comezón en el sitio de inyección:**
  **0: Ninguno.**
  **1: Leve**: Sensación de comezón que no interfiere ni me impide el desarrollo de mis actividades normales.
  **2: Moderado**: Sensación de comezón que interfiere con mis actividades normales.
  **3: Severo:** Sensación de comezón que impide el desarrollo de mis actividades normales.

Versión 1, 01-Mar-2021.

9

| No. Protocolo: | TARJETA DIARIO Número de dosis de vacuna: | Número del sujeto: *Para ser completado por el personal del estudio. |
|---|---|---|

Avimex®

**SÍNTOMAS LOCALES:**

***Para ser completado por el personal del estudio:**
**Fecha de vacunación : ____________ Sitio de inyección (Ubicación):________________ Lado:____________**

| | Día 1 | Día 2 | Día 3 | Día 4 | Día 5 | Día 6 | Día 7 | Después del Día 7: Persiste | Peor intensidad/ Mayor tamaño | Fecha de finalización | ¿Recibió atención médica? | Tipo de atención médica: |
|---|---|---|---|---|---|---|---|---|---|---|---|---|
| Enrojecimiento en el sitio de inyección: • Intensidad en mm. | mm | mm | mm | mm | mm | mm | mm | ☐ NO ☐ SI | mm | Marque esta casilla si aún persiste: ☐ | ☐ NO ☐ SI | ☐ Hospitalaria ☐ Urgencias ☐ Consulta Medica |
| Dolor en el sitio de inyección: • Intensidad (0/1/2/3). | 0/1/2/3 | 0/1/2/3 | 0/1/2/3 | 0/1/2/3 | 0/1/2/3 | 0/1/2/3 | 0/1/2/3 | ☐ NO ☐ SI | 0/1/2/3 | ☐ | ☐ NO ☐ SI | ☐ Hospitalaria ☐ Urgencias ☐ Consulta Medica |
| Inflamación en el sitio de inyección: • Intensidad en mm. | mm | mm | mm | mm | mm | mm | mm | ☐ NO ☐ SI | mm | ☐ | ☐ NO ☐ SI | ☐ Hospitalaria ☐ Urgencias ☐ Consulta Medica |
| Comezón en el sitio de inyección: • Intensidad (0/1/2/3). | 0/1/2/3 | 0/1/2/3 | 0/1/2/3 | 0/1/2/3 | 0/1/2/3 | 0/1/2/3 | 0/1/2/3 | ☐ NO ☐ SI | 0/1/2/3 | ☐ | ☐ NO ☐ SI | ☐ Hospitalaria ☐ Urgencias ☐ Consulta Medica |

Versión 1, 01-Mar-2021.

10

**Instrucciones sobre como llenarlo:**

**Síntomas Generales**

Avimex®

- **¿Cómo Completar el Valor "Diario"?**
- Anote el valor para cada síntoma y cada síntoma y cada día (media o intensidad). **No deje ningún campo vacío.**

| | Día 1 | Día 2 | Día 3 | Día 4 | Día 5 | Día 6 | Día 7 | Después del Día 7: Persiste | Peor intensidad/ Mayor tamaño | Fecha de finalización | ¿Recibió atención médica? | Tipo de atención médica: |
|---|---|---|---|---|---|---|---|---|---|---|---|---|
| Fatiga: • Intensidad (0/1/2/3). | 0/1/2/3 | 0/1/2/3 | 0/1/2/3 | 0/1/2/3 | 0/1/2/3 | 0/1/2/3 | 0/1/2/3 | ☐ NO ☐ SI | 0/1/2/3 | Marque esta casilla si aún persiste: ☐ | ☐ NO ☐ SI | ☐ Hospitalaria ☐ Urgencias ☐ Consulta Medica |

**Instrucciones sobre como llenarlo:**
**Síntomas Generales: Temperatura.**

Avimex®

- Utilice el termómetro digital que se le proporcionó como parte del estudio.
- Tome su temperatura todos los días por la tarde desde el día de vacunación (Columna Día 1) y durante los siguientes 6 días y anote los valores.
- La vía para la toma de temperatura digital será en la frente. Se le solicita haga todo lo posible por siempre tomar su temper atura de acuerdo con las indicaciones. Por favor indique la ubicación de cada medición.
- Si tomó su temperatura más de una vez al día, anote la más alta.

- Ejemplo:
- A las 8am: 37.1
- A la 1pm : 37.4
- A la 7pm : 37.6

= **37.6 se registrara en la Columna de Día 1.**

- Escribir NT (No tomada) si no se tomó la temperatura.

***Debe tomar siempre la temperatura en el mismo lugar corporal.**

| | Día 1 | Día 2 | Día 3 | Día 4 | Día 5 | Día 6 | Día 7 | Después del Día 7: Persiste 38.0°C o más alta | Temperatura más alta | Fecha de finalización | ¿Recibió atención médica? | Tipo de atención médica: | Relación con la vacuna del estudio *A ser llenado por el personal del estudio |
|---|---|---|---|---|---|---|---|---|---|---|---|---|---|
| Temperatura: | 37.6 | 37.2 | NT | 37.2 | 37.1 | NT | 37.2 | ☐ NO ☐ SI | | Marque esta casilla si aún persiste: ☐ | ☐ NO ☐ SI | ☐ Hospitalaria ☐ Urgencias ☐ Consulta Medica | ☐ NO ☐ SI |

| No. Protocolo: | TARJETA DIARIO Número de dosis de vacuna: | Número del sujeto: *Para ser completado por el personal del estudio. |
|---|---|---|

Avimex®

**SÍNTOMAS GENERALES:**

***Para ser completado por el personal del estudio:**
**Fecha de vacunación : ____________ Sitio de inyección (Ubicación):________________ Lado:____________**
***La Ubicación debe ser siempre la misma.**

| | Día 1 | Día 2 | Día 3 | Día 4 | Día 5 | Día 6 | Día 7 | Después del Día 7: Persiste 38.0°C o más alta | Temperatura más alta | Fecha de finalización | ¿Recibió atención médica? | Tipo de atención médica: | Relación con la vacuna del estudio *A ser llenado por el personal del estudio |
|---|---|---|---|---|---|---|---|---|---|---|---|---|---|
| Temperatura: | | | | | | | | ☐ NO ☐ SI | | Marque esta casilla si aún persiste: ☐ | ☐ NO ☐ SI | ☐ Hospitalaria ☐ Urgencias ☐ Consulta Medica | ☐ NO ☐ SI |

*Aclaración (es) solo para el personal del estudio:

Versión 1, 01-Mar-2021.

13

**SÍNTOMAS GENERALES**

Avimex®

**Definición de "Intensidad":**

- **Fatiga:**
  **0: Nada de fatiga.**
  **1: Leve**: Fatiga
  **2: Moderado**: Fatiga que interfiere con mis actividades normales.
  **3: Grave**: Fatiga en reposo que me impide el desarrollo de mis actividades normales.

- **Síntomas gastrointestinales (incluyen náuseas, diarrea o dolor abdominal):**
  **0: Ninguno.**
  **1: Leve**: Síntomas gastrointestinales que no interfieren ni me impiden el desarrollo de mis actividades cotidianas normales.
  **2: Moderado**: Síntomas gastrointestinales que interfieren con mis actividades cotidianas.
  **3: Grave**: Síntomas gastrointestinales que me impiden el desarrollo de mis actividades cotidianas normales.

- **Dolor de cabeza:**
  **0: Ninguno.**
  **1: Leve**: Dolor de cabeza que se tolera fácilmente.
  **2: Moderado**: Dolor de cabeza que interfiere con las actividades normales.
  **3: Grave:** Dolor de cabeza que impide el desarrollo de las actividades normales.

Versión 1, 01-Mar-2021.

14

**SÍNTOMAS GENERALES**

Avimex®

- **Definición de "Intensidad":**

- **Dolor de muscular o corporal:**
  **0: Nada de dolor muscular o corporal.**
  **1: Leve**: Dolor muscular o corporal que no interfiere ni me impide el desarrollo de mis actividades cotidianas normales.
  **2: Moderado**: Dolor muscular o corporal que interfiere con mis actividades normales.
  **3: Grave**: Dolor muscular o corporal que me impide el desarrollo de mis actividades normales.

- **Escalofríos:**
  **0: Ninguno.**
  **1: Escalofríos que se toleran fácilmente.**
  **2: Escalofríos que interfieren con mis actividades normales.**
  **3: Escalofríos que me impiden el desarrollo de mis actividades normales.**

- **Malestar:**
  **0: Ninguno.**
  **1: Malestar que se toleran fácilmente.**
  **2: Malestar que interfieren con mis actividades normales.**
  **3: Malestar que me impiden el desarrollo de mis actividades normales.**

**SÍNTOMAS GENERALES**

Avimex®

- **Definición de "Intensidad":**

- **Tos:**
  **0: Nada de tos.**
  **1: Leve**: Tos que no interfiere ni me impide el desarrollo de mis actividades cotidianas normales.
  **2: Moderado**: Tos que interfiere con mis actividades normales.
  **3: Grave**: Tos que me impide el desarrollo de mis actividades normales.

- **Falta de aire o dificultad para respirar (dificultad respiratoria) :**
  **0: Nada de falta de aire o dificultad para respirar.**
  **1: Leve**: Falta de aire o dificultad respiratoria que no interfiere ni impide el desarrollo de mis actividades cotidianas normales.
  **2: Moderado**: Falta de aire o dificultad respiratoria que interfiere con mis actividades cotidianas.
  **3: Grave**: Falta de aire o dificultad respiratoria significativa en estado de reposo que me impide desarrollo de mis actividades cotidianas normales.

- **Nueva pérdida del gusto u olfato:**
  **0: Nada de nueva pérdida del gusto u olfato.**
  **1: Leve**: Nueva pérdida del gusto u olfato que se tolera fácilmente.
  **2: Moderado**: Nueva pérdida del gusto u olfato que interfiere con las actividades normales.
  **3: Grave:** Nueva pérdida del gusto u olfato que impide el desarrollo de mis actividades cotidianas normales.

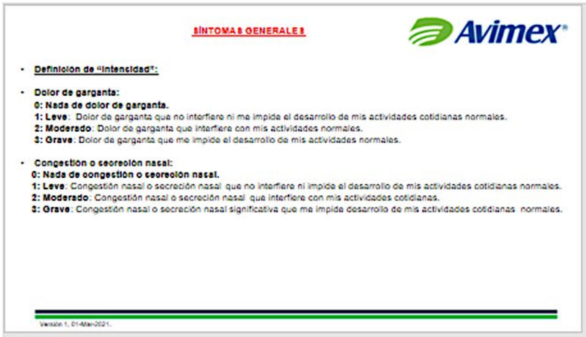
SÍNTOMAS GENERALES
Avimex
Definición de "Intencidad":
Dolor de garganta:
0: Nada de dolor de garganta.
1: Leve: Dolor de garganta que no interfiere ni me impide el desarrollo de mis actividades cotidianas normales.
2: Moderado: Dolor de garganta que interfiere con mis actividades normales.
3: Grave: Dolor de garganta que me impide el desarrollo de mis actividades normales.
Congestión o secreción nasal:
0: Nada de congestión o secreción nasal.
1: Leve: Congestión nasal o secreción nasal que no interfiere ni impide el desarrollo de mis actividades cotidianas normales.
2: Moderado: Congestión nasal o secreción nasal que interfiere con mis actividades cotidianas.
3: Grave: Congestión nasal o secreción nasal significativa que me impide desarrollo de mis actividades cotidianas normales.
Versión 1, 01-Mar-2021.

17

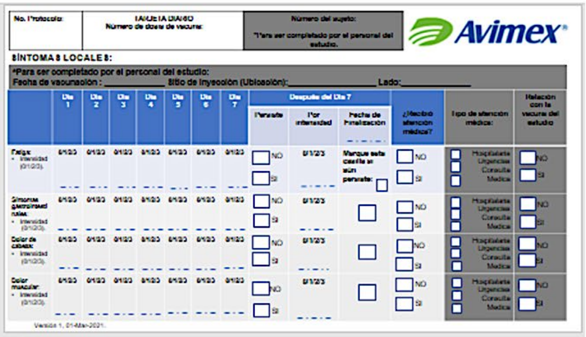
No. Protocolo:
TARJETA DIARIO
Número de dosis de vacuna:
Número del sujeto:
*Para ser completado por el personal del estudio.
Avimex
SÍNTOMAS LOCALES:
*Para ser completado por el personal del estudio:
Fecha de vacunación:
Sitio de inyección (Ubicación):
Lado:
Después del Día 7
Persiste
Por intensidad
Fecha de finalización
¿Recibió atención médica?
Tipo de atención médica:
Relación con la vacuna del estudio
Fatiga:
Síntomas gastrointestinales:
Dolor de cabeza:
Dolor muscular:
Marque esta casilla si aún persiste:
NO
SI
Hospitalización
Urgencias
Consulta Médica
Versión 1, 01-Mar-2021.

18

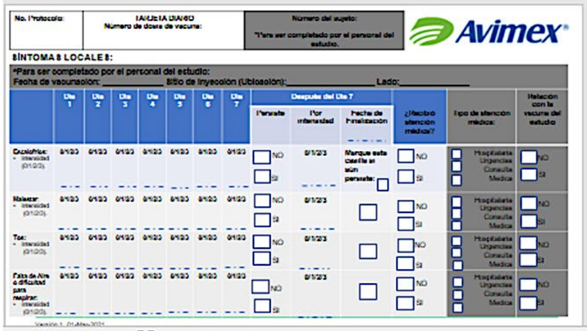
No. Protocolo:
TARJETA DIARIO
Número de dosis de vacuna:
Número del sujeto:
*Para ser completado por el personal del estudio.
Avimex
SÍNTOMAS LOCALES:
*Para ser completado por el personal del estudio:
Fecha de vacunación:
Sitio de inyección (Ubicación):
Lado:
Después del Día 7
Persiste
Por intensidad
Fecha de finalización
¿Recibió atención médica?
Tipo de atención médica:
Relación con la vacuna del estudio
Escalofríos:
Malestar:
Tos:
Falta de Aire o dificultad para respirar:
Marque esta casilla si aún persiste:
NO
SI
Hospitalización
Urgencias
Consulta Médica
Versión 1, 01-Mar-2021.

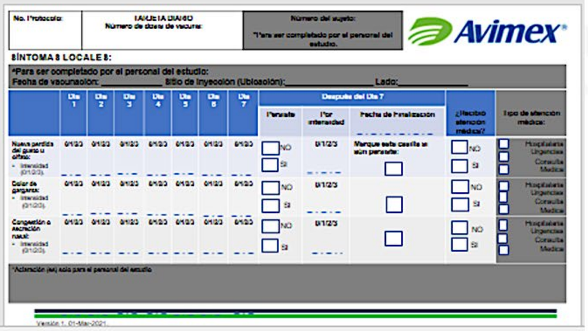
No. Protocolo:
TARJETA DIARIO
Número de dosis de vacuna:
Número del sujeto:
*Para ser completado por el personal del estudio.
Avimex
SÍNTOMAS LOCALES:
*Para ser completado por el personal del estudio:
Fecha de vacunación:
Sitio de inyección (Ubicación):
Lado:
Después del Día 7
Persiste
Por intensidad
Fecha de finalización
¿Recibió atención médica?
Tipo de atención médica:
Nueva pérdida del gusto u olfato:
Dolor de garganta:
Congestión o secreción nasal:
Marque esta casilla si aún persiste:
NO
SI
Hospitalización
Urgencias
Consulta Médica
Versión 1, 01-Mar-2021.

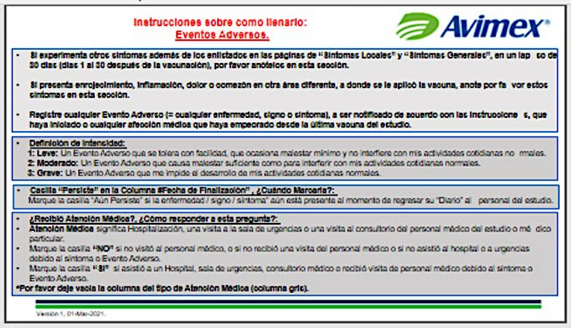
Instrucciones sobre como llenarlo:
Eventos Adversos.
Avimex
Si experimenta otros síntomas además de los enlistados en las páginas de "Síntomas Locales" y "Síntomas Generales", en un lap so de 30 días (días 1 al 30 después de la vacunación), por favor anótelos en esta sección.
Si presenta enrojecimiento, inflamación, dolor o comezón en otra área diferente, a donde se le aplicó la vacuna, anote por fa vor estos síntomas en esta sección.
Registre cualquier Evento Adverso (= cualquier enfermedad, signo o síntoma), a ser notificado de acuerdo con las instruccione s, que haya iniciado o cualquier afección médica que haya empeorado desde la última vacuna del estudio.
Definición de Intensidad:
1: Leve: Un Evento Adverso que se tolera con facilidad, que ocasiona malestar mínimo y no interfiere con mis actividades cotidianas no rmales.
2: Moderado: Un Evento Adverso que causa malestar suficiente como para interferir con mis actividades cotidianas normales.
3: Grave: Un Evento Adverso que me impide el desarrollo de mis actividades cotidianas normales.
Casilla "Persiste" en la Columna #Fecha de Finalización" , ¿Cuándo Marcarla?:
Marque la casilla "Aún Persiste" si la enfermedad / signo / síntoma" aún está presente al momento de regresar su "Diario" al personal del estudio.
¿Recibió Atención Médica?, ¿Cómo responder a esta pregunta?:
Atención Médica significa Hospitalización, una visita a la sala de urgencias o una visita al consultorio del personal médico del estudio o mé dico particular.
Marque la casilla "NO" si no visitó al personal médico, o si no recibió una visita del personal médico o si no asistió al hospital o a urgencias debido al síntoma o Evento Adverso.
Marque la casilla "SI" si asistió a un Hospital, sala de urgencias, consultorio médico o recibió visita de personal médico debido al síntoma o Evento Adverso.
*Por favor deje vacía la columna del tipo de Atención Médica (columna gris).
Versión 1, 01-Mar-2021.

21

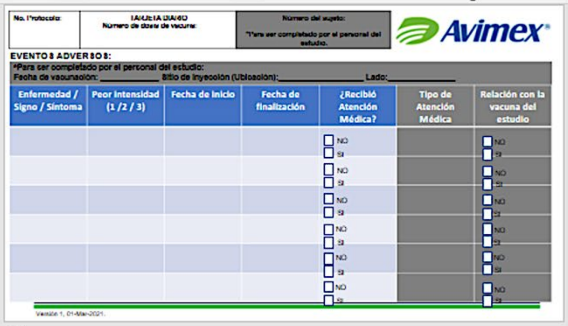
No. Protocolo:
TARJETA DIARIO
Número de dosis de vacuna:
Número del sujeto:
*Para ser completado por el personal del estudio.
Avimex
EVENTOS ADVERSOS:
*Para ser completado por el personal del estudio:
Fecha de vacunación:
Sitio de inyección (Ubicación):
Lado:
Enfermedad / Signo / Síntoma
Peor intensidad (1 /2 / 3)
Fecha de inicio
Fecha de finalización
¿Recibió Atención Médica?
Tipo de Atención Médica
Relación con la vacuna del estudio
NO
SI
Versión 1, 01-Mar-2021.

22

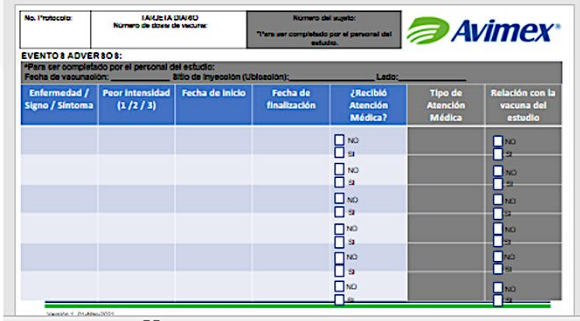
No. Protocolo:
TARJETA DIARIO
Número de dosis de vacuna:
Número del sujeto:
*Para ser completado por el personal del estudio.
Avimex
EVENTOS ADVERSOS:
*Para ser completado por el personal del estudio:
Fecha de vacunación:
Sitio de inyección (Ubicación):
Lado:
Enfermedad / Signo / Síntoma
Peor intensidad (1 /2 / 3)
Fecha de inicio
Fecha de finalización
¿Recibió Atención Médica?
Tipo de Atención Médica
Relación con la vacuna del estudio
NO
SI
Versión 1, 01-Mar-2021.

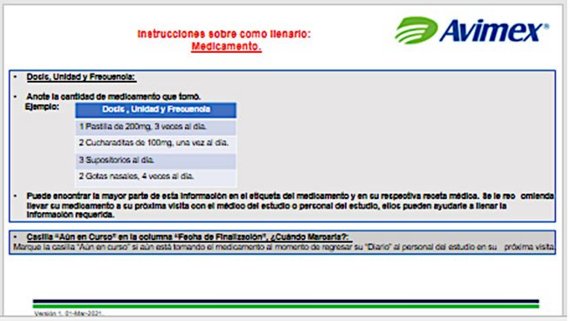
Instrucciones sobre como llenarlo:
Medicamento.
Avimex
Dosis, Unidad y Frecuencia:
Anote la cantidad de medicamento que tomó.
Ejemplo:
Dosis , Unidad y Frecuencia
1 Pastilla de 200mg, 3 veces al día.
2 Cucharaditas de 100mg, una vez al día.
3 Supositorios al día.
2 Gotas nasales, 4 veces al día.
Puede encontrar la mayor parte de esta información en el etiqueta del medicamento y en su respectiva receta médica. Se le rec omienda llevar su medicamento a su próxima visita con el médico del estudio o personal del estudio, ellos pueden ayudarle a llenar la información requerida.
Casilla "Aún en Curso" en la columna "Fecha de Finalización", ¿Cuándo Marcarla?:
Marque la casilla "Aún en curso" si aún está tomando el medicamento al momento de regresar su "Diario" al personal del estudio en su próxima visita.
Versión 1, 01-Mar-2021.

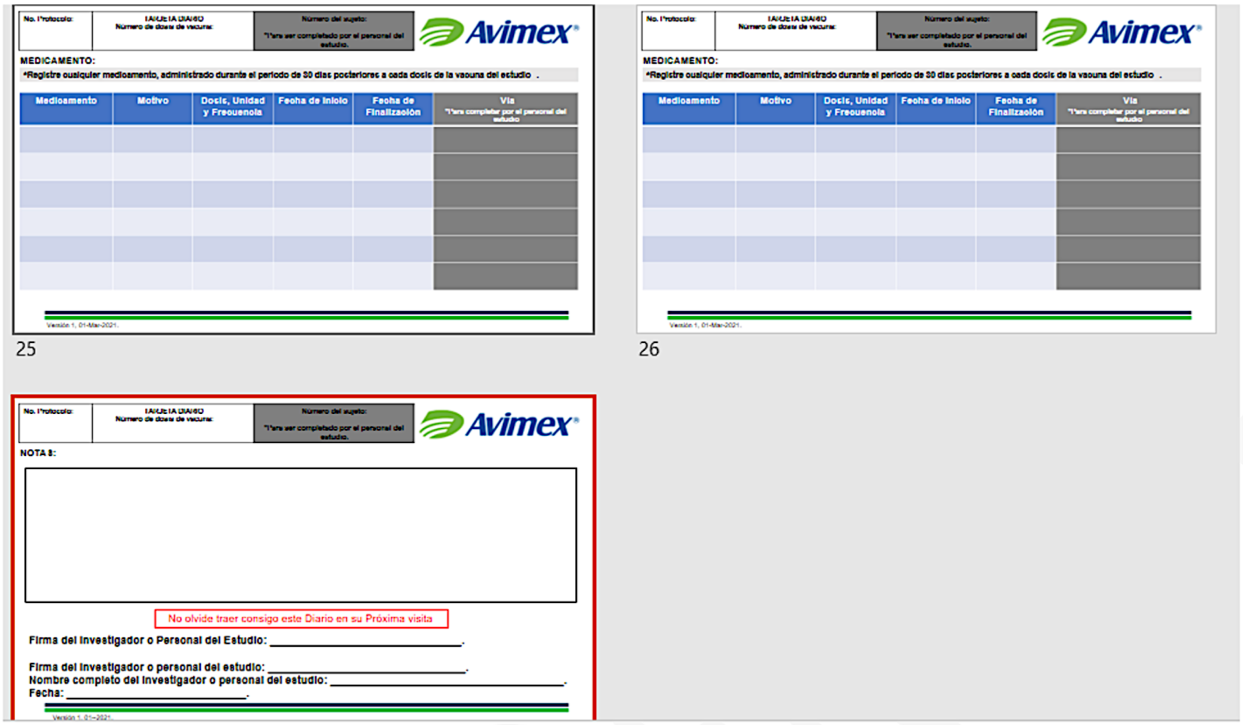

| No. Protocolo: | TARJETA DIARIO<br>Número de dosis de vacuna: | Número del sujeto:<br>*Para ser completado por el personal del estudio. | Avimex |
|---|---|---|---|

MEDICAMENTO:

*Registre cualquier medicamento, administrado durante el periodo de 30 días posteriores a cada dosis de la vacuna del estudio .

| Medicamento | Motivo | Dosis, Unidad y Frecuencia | Fecha de Inicio | Fecha de Finalización | Vía<br>*Para completar por el personal del estudio |
|---|---|---|---|---|---|
| | | | | | |
| | | | | | |
| | | | | | |
| | | | | | |
| | | | | | |
| | | | | | |

Versión 1, 01-Mar-2021.

25

| No. Protocolo: | TARJETA DIARIO<br>Número de dosis de vacuna: | Número del sujeto:<br>*Para ser completado por el personal del estudio. | Avimex |
|---|---|---|---|

MEDICAMENTO:

*Registre cualquier medicamento, administrado durante el periodo de 30 días posteriores a cada dosis de la vacuna del estudio .

| Medicamento | Motivo | Dosis, Unidad y Frecuencia | Fecha de Inicio | Fecha de Finalización | Vía<br>*Para completar por el personal del estudio |
|---|---|---|---|---|---|
| | | | | | |
| | | | | | |
| | | | | | |
| | | | | | |
| | | | | | |
| | | | | | |

Versión 1, 01-Mar-2021.

26

| No. Protocolo: | TARJETA DIARIO<br>Número de dosis de vacuna: | Número del sujeto:<br>*Para ser completado por el personal del estudio. | Avimex |
|---|---|---|---|

NOTA 8:

No olvide traer consigo este Diario en su Próxima visita

Firma del Investigador o Personal del Estudio: ______________________________.

Firma del Investigador o personal del estudio: ______________________________.
Nombre completo del Investigador o personal del estudio: ______________________________.
Fecha: ______________________.

Versión 1, 01–2021.

# ANNEX 5
# Scale of COVID-19 infection risk

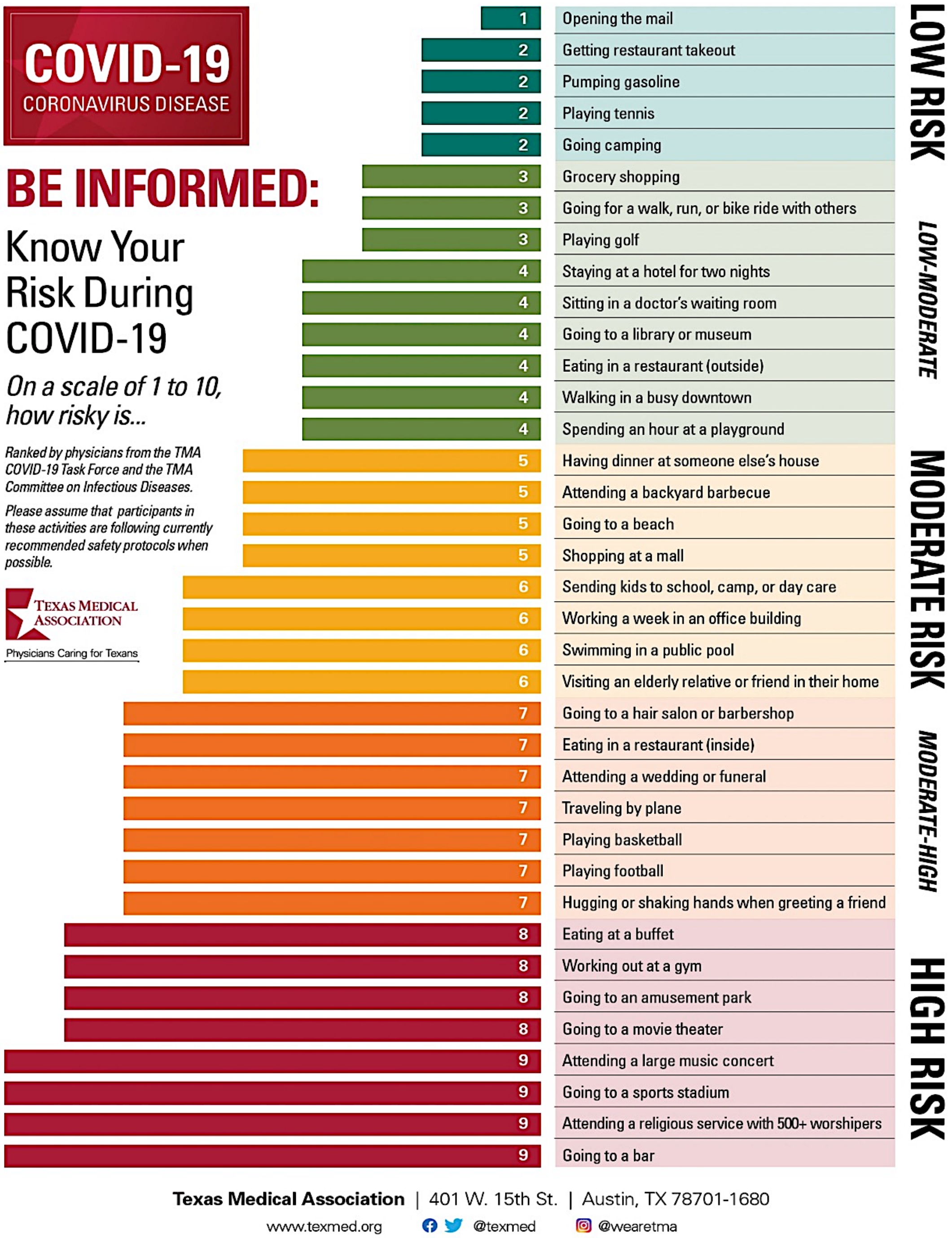
COVID-19
CORONAVIRUS DISEASE
BE INFORMED:
Know Your Risk During COVID-19
On a scale of 1 to 10, how risky is...
Ranked by physicians from the TMA COVID-19 Task Force and the TMA Committee on Infectious Diseases.
Please assume that participants in these activities are following currently recommended safety protocols when possible.
Texas Medical Association
Physicians Caring for Texans
LOW RISK
1 Opening the mail
2 Getting restaurant takeout
2 Pumping gasoline
2 Playing tennis
2 Going camping
LOW-MODERATE
3 Grocery shopping
3 Going for a walk, run, or bike ride with others
3 Playing golf
4 Staying at a hotel for two nights
4 Sitting in a doctor's waiting room
4 Going to a library or museum
4 Eating in a restaurant (outside)
4 Walking in a busy downtown
4 Spending an hour at a playground
MODERATE RISK
5 Having dinner at someone else's house
5 Attending a backyard barbecue
5 Going to a beach
5 Shopping at a mall
6 Sending kids to school, camp, or day care
6 Working a week in an office building
6 Swimming in a public pool
6 Visiting an elderly relative or friend in their home
MODERATE-HIGH
7 Going to a hair salon or barbershop
7 Eating in a restaurant (inside)
7 Attending a wedding or funeral
7 Traveling by plane
7 Playing basketball
7 Playing football
7 Hugging or shaking hands when greeting a friend
HIGH RISK
8 Eating at a buffet
8 Working out at a gym
8 Going to an amusement park
8 Going to a movie theater
9 Attending a large music concert
9 Going to a sports stadium
9 Attending a religious service with 500+ worshipers
9 Going to a bar
Texas Medical Association | 401 W. 15th St. | Austin, TX 78701-1680
www.texmed.org @texmed @wearetma
